# The genomic basis for colonizing the freezing Southern Ocean revealed by Antarctic toothfish and Patagonian robalo genomes

**DOI:** 10.1093/gigascience/giz016

**Published:** 2019-01-31

**Authors:** Liangbiao Chen, Ying Lu, Wenhao Li, Yandong Ren, Mengchao Yu, Shouwen Jiang, Yanxia Fu, Jian Wang, Sihua Peng, Kevin T Bilyk, Katherine R Murphy, Xuan Zhuang, Mathias Hune, Wanying Zhai, Wen Wang, Qianghua Xu, Chi-Hing Christina Cheng

**Affiliations:** 1 Internal Research Center for Marine Bioscience (Ministry of Science and Technology), Shanghai Ocean University, Shanghai, China; 2 Key Laboratory of Exploration and Utilization of Aquatic Genetic Resources (Ministry of Education) and International Research Center for Marine Biosciences (Ministry of Science and Technology) at Shanghai Ocean University, Shanghai, China; 3 Laboratory for Marine Biology and Biotechnology, Qingdao National Laboratory for Marine Science and Technology, Qingdao, China; 4 Kunming Institute of Zoology, Chinese Academy of Sciences, Kuming, China; 5 Department of Animal Biology, University of Illinois at Urbana-Champaign, IL, USA; 6 Fundación Ictiológica, Providencia, Santiago, Chile

**Keywords:** adaptive radiation, climate change, genome, oxidative stress, Antarctic notothenioids

## Abstract

**Background:**

The Southern Ocean is the coldest ocean on Earth but a hot spot of evolution. The bottom-dwelling Eocene ancestor of Antarctic notothenioid fishes survived polar marine glaciation and underwent adaptive radiation, forming >120 species that fill all water column niches today. Genome-wide changes enabling physiological adaptations and the rapid expansion of the Antarctic notothenioids remain poorly understood.

**Results:**

We sequenced and compared 2 notothenioid genomes—the cold-adapted and neutrally buoyant Antarctic toothfish *Dissostichus mawsoni* and the basal Patagonian robalo *Eleginops maclovinus*, representing the temperate ancestor. We detected >200 protein gene families that had expanded and thousands of genes that had evolved faster in the toothfish, with diverse cold-relevant functions including stress response, lipid metabolism, protein homeostasis, and freeze resistance. Besides antifreeze glycoprotein, an eggshell protein had functionally diversified to aid in cellular freezing resistance. Genomic and transcriptomic comparisons revealed proliferation of selcys–transfer RNA genes and broad transcriptional upregulation across anti-oxidative selenoproteins, signifying their prominent role in mitigating oxidative stress in the oxygen-rich Southern Ocean. We found expansion of transposable elements, temporally correlated to Antarctic notothenioid diversification. Additionally, the toothfish exhibited remarkable shifts in genetic programs towards enhanced fat cell differentiation and lipid storage, and promotion of chondrogenesis while inhibiting osteogenesis in bone development, collectively contributing to the achievement of neutral buoyancy and pelagicism.

**Conclusions:**

Our study revealed a comprehensive landscape of evolutionary changes essential for Antarctic notothenioid cold adaptation and ecological expansion. The 2 genomes are valuable resources for further exploration of mechanisms underlying the spectacular notothenioid radiation in the coldest marine environment.

## Introduction

The Southern Ocean (SO) surrounding Antarctica is the coldest body of water on Earth, having been isolated from other world oceans by the Antarctic circumpolar current beginning in the early Oligocene ∼32 million years ago (mya) [1]. The formation of the Antarctic circumpolar current also impeded species dispersal across the Antarctic polar front, and mass extinction of the Antarctic-sequestered fish taxa occurred upon marine glaciation [2]. The rich cosmopolitan fish fauna prior to the isolation of Antarctica is represented today by a single predominant group of related fish species—the Antarctic notothenioids. From a common temperate ancestor, likely a swim bladderless, bottom-dwelling perciform species of the Eocene age [3], the Antarctic notothenioids have evolved to become highly adapted to life in unyielding cold, spectacularly diverse in sizes and morphological innovations, and diversified into all water column habitats, epitomizing an adaptive radiation and a rare marine species flock [4]. Abundant in fish biomass (>90% of random catch) and species (≥128), they are vital in sustaining the contemporary SO food web [2, 5].

What evolutionary processes and mechanisms propelled the Antarctic notothenioid radiation replete with extraordinary trait diversification during its evolutionary history remain fascinating unanswered questions. Two conspicuous trait outcomes—the evolutionary gain of the novel antifreeze glycoprotein (*AFGP*) gene and function that averted otherwise inescapable death from freezing [6, 7], and exploitation of open niches vacated by extinction of fishes lacking freeze resistance, have been recognized as major contributors to the Antarctic notothenioid radiation [8, 9]. However, little is known of the myriad subtler adaptive changes that must also have evolved in response to challenges from freezing temperatures and the associated high oxygen concentration—the 2 foremost modalities of selection pressure from the SO environments that would pervade all levels of organismal functions, from molecules to cells to system physiology. Another prominent hallmark of notothenioid adaptive radiation is the secondary acquisition of pelagicism in some lineages, enabling their ecological expansion from bottom habitats of their negatively buoyant ancestor to upper water column niches. What evolutionary changes occurred in the cellular and developmental programs that enabled neutral buoyancy and secondary pelagicism are also unknown.

To address these fundamental, system-wide questions about Antarctic notothenioid evolution, whole-genome sequences of multiple and appropriately chosen species from the diverse Antarctic notothenioids are essential. Thus far, whole-genome sequence analysis has been reported for only 1 notothenioid species, the Antarctic rock cod *Notothenia coriiceps* [10]. A major histocompatibility complex gene locus from *Chaenocephalus aceratus* was also reported [11]. The *N. coriiceps* genome provided the key inference that the fast-evolving hemoglobin and mitochondrial proteins are adaptive in increasing efficiency of aerobic cellular respiration in the freezing environment. *N. coriiceps* is not known to occur in the high-latitude Antarctic coastal waters. Instead, it is widely distributed in the lower latitude waters of the Antarctic Peninsula archipelago and the Scotia Arc islands, reaching localities north of the polar front around sub-Antarctic islands in the Indian Ocean sector [12], a distribution pattern that suggests a considerable degree of thermal plasticity in this species. It is a heavy, bottom fish and one of the hardest boned Antarctic notothenioids [13], reminiscent of the benthic ancestor. To gain insights into evolutionary adaptations in the most cold-adapted and stenothermal Antarctic notothenioids, as well as into the evolutionary changes leading to acquisition of neutral buoyancy that enabled the transition from the ancestral benthic existence to a pelagic life history, a different and more appropriate model Antarctic notothenioid species would be required.

The Antarctic toothfish *Dissostichus mawsoni* (NCBI:txid6530, Fishbase ID:7039) that grows to giant sizes (2.0 m in length and 140 kg in mass) is an iconic species of the Antarctic notothenioid radiation, with wide distributions in freezing waters of high-latitude Antarctic coasts, as far south as 77.5 S (McMurdo Sound), the southern limit of Antarctic marine life. It thus exemplifies the stenothermal cold-adapted character state. Despite its large size, it is the only notothenioid species that achieved complete neutral buoyancy as adults [14, 15]; thus, this species serves as the best model for examining the evolutionary underpinning of secondary pelagicism in the Antarctic clade. In addition, to discern evolutionary changes from the ancestral temperate state to the derived polar state driven by selection in the cold, oxygen-rich SO environment, a closely related basal non-Antarctic notothenioid comparison species would improve the discriminating power of analyses of genome evolution. The most appropriate species for this purpose is a South American notothenioid, the Patagonian robalo *Eleginops maclovinus* (NCBI:txid56733, Fishbase ID:466) , which is the sole species in the basal family Eleginopsidae. Also known as the Patagonian blenny, the lineage diverged prior to the isolation of Antarctica, and *E. maclovinus* is phylogenetically the closest sister species to the modern Antarctic clade [3]. Thus, its genome is the best representative of the temperate character of the most recent common ancestor of the Antarctic notothenioids. We conducted genome sequencing and comparative analyses of these 2 strategically selected species, together with extensive transcriptomic characterizations to profile relevant functional outcomes of the genomic changes. Our results provide several new key insights into evolutionary adaptation and secondary pelagicism of the Antarctic notothenioids in the isolated and extremely cold SO environment.

## Materials and Methods

### Specimens, sampling, and DNA and RNA isolation

Antarctic toothfish *D. mawsoni* was collected using vertical setline through drilled hole in sea ice of McMurdo Sound, Antarctica (77 53 S, 166 34.4 E and vicinity), during austral summer field seasons (October through December). Specimens were transported to the aquarium facility in the US National Science Foundation Crary Lab at McMurdo Station and kept in ambient (−1.6°C) flow through seawater tanks, and killed at 2–4 weeks after capture for blood and tissue sampling. The temperate basal notothenioid *E. maclovinus* was collected by rod and reel in the Patagonian waters of southern Chile during austral winter (June) and transported to the National Science Foundation Research Vessel Laurence Gould at Punta Arenas in a large, aerated Styrofoam cooler of ambient water (∼8°C), where specimens were killed and sampled within a few days prior to southbound transit for winter field season. To obtain tissues from the large-sized *D. mawsoni*, live specimen was anesthetized with MS222 (tricaine methanesulfonate) inside an ambient seawater–filled floating sheet plastic tubing in the aquarium tank. The anesthetized specimen was then put on a V-shaped trough for dissection. Tissues were quickly removed and cut into small pieces on ice, and immediately immersed and shaken in ≥10 volumes of prechilled (−20°C) 90% ethanol (made with 100% pure ethanol and sterilized MilliQ Type 1 water). The ethanol was replaced with a fresh volume within 10 minutes, and again at 2–3 and 12 hours later. This preservation method serially desiccates the tissue and effectively inactivates tissue nucleases. The tissue samples were kept in a −20°C freezer throughout the serial preservation process and then stored at −20°C until use. To obtain tissues from *E. maclovinus*, MS222-anesthetized specimen was quickly dissected on ice and preserved at −20°C as described for *D. mawsoni*. The ethanol-preserved tissues were shipped back to the University of Illinois on dry ice.

RNA for transcriptome sequencing was isolated from −20°C ethanol-preserved tissues using Trizol (Invitrogen, Carlsbad, California, USA)) and quality verified by visualization on gel electrophoresis and an Agilent BioAnalyzer. Collection, handling, and sampling of the Antarctic toothfish and South American *E. maclovinus* in this study were carried out in compliance with protocol No. 12123 approved by the University of Illinois Institutional Animal Care and Use Committee. Additional juvenile specimens of *D. mawsoni* were collected by trawl from the waters of the Antarctic Peninsula during the same winter season and sampled on shipboard shortly after capture. The dissected carcasses of *E. maclovinus* and juvenile *D. mawsoni* were kept frozen at −80°C, which provided the pelvic bone samples for immunohistochemical detection for expression of candidate genes in bone development. To preserve high molecular weight DNA for genome sequencing, red blood cells of each species were washed with notothenioid saline (0.1 M sodium phosphate buffer, pH 8.0, adjusted to 420 mOsm with NaCl for *E. maclovinus*, and 540 mOsm for *D. mawsoni*), and then embedded in 1% melt agarose to provide ∼20 μg DNA per 90-μL block using BioRad plug molds (CHEF Mammalian Genomic DNA Plug Kit No. 1703591, Bio-rad, Hercules, CA, USA). The embedded cells were lysed *in situ* and the DNA in the agarose blocks was preserved following Amemiya et al (1996) [16]. To recover high molecular weight DNA, the agarose plugs were digested with β-agarase (NEB, Ipswich, MA, USA) followed by phenol extraction and dialysis, and quality verified using pulsed-field electrophoresis.

### Sequencing and genome assembly

The sequencing libraries with insert sizes of 170, 250, and 500 base pairs (bp) were prepared for sequencing of the paired-end reads, following a modified version of the manufacturer's protocol (Illumina, San Diego, CA, USA). An integrated protocol from the Mate-Pair Library v2 Sample Preparation Guide (Illumina) and the Paired-End Library Preparation Method Manual (Roche, Branford, Connecticut, USA)) was used to prepare mate-pair libraries with insert sizes of 3, 6, 10, 15, and 20 kb (Additional file 1: Table S1b, S1c). For the transcriptome sequencing, Poly(A)+ messenger RNA (mRNA) was purified using the DynaBeads mRNA Purification kit (Life Technologies, Carlsbad, CA). Paired-end complementary DNA libraries were constructed using the RNA sequencing (RNA-Seq) Next-Generation Sequencing Library Preparation Kit for Whole-Transcriptome Discovery (Gnomegen, San Diego, CA). All of the libraries are sequenced on an Illumina HiSeq 1500 sequencer. The *D. mawsoni* genome was assembled using SOAPdenovo (SOAPdenovo, RRID:SCR_010752) [17] to build the contigs and SSPACE (SSPACE, RRID:SCR_005056) [18] to scaffold the contigs. The *E. maclovinus* genome was assembled using Platanus (Platanus, RRID:SCR_015531) to build the contigs, and SSPACE to scaffold the contigs.

### Annotation of the genome

We identified repeats, protein-coding genes, and noncoding RNA in the genome assemblies of the 2 species. First, a *de novo* repeat annotation of *D. mawsoni* and *E. maclovinus* genomes was carried out by successively using RepeatModeler (RepeatModeler, RRID:SCR_015027) (version 1.0.8) and RepeatMasker (RepeatMasker, RRID:SCR_012954) (version 4.0.5). *De novo* repeat libraries of the 2 species were constructed with 2 complementary programs, RECON (RECON, RRID:SCR_006345) [19] and RepeatScout (RepeatScout, RRID:SCR_014653) [20] implemented in the RepeatModeler package. The generated consensus sequences were manually checked by aligning to the Repbase transposable element (TE) library [21] and genes from the NCBI database (nt and nr). The *D. mawsoni* and *E. maclovinus* repeat library consisted of 975 and 676 consensus sequences with classification information, respectively, which were used to run RepeatMasker on the assembled scaffolds. Secondly, protein-coding genes were predicted using a combination of homology-based and *de novo* approaches. GLEAN was used to create a consensus gene set by integrating evidence from each prediction. Then RNA-Seq data were used to rectify gene models. Generated coding genes were aligned to known protein databases, including InterPro [22], Kyoto Encyclopedia of Genes and Genomes (KEGG) [23], and Uniprot [24], and functional assignment was based on that of the best database match. Thirdly, the transfer RNA (tRNA) genes were predicted with tRNAscan-SE (tRNAscan-SE, RRID:SCR_010835) [25]. Aligning the ribosomal rNA (rRNA) template sequences from fishes using BlastN (BlastN, RRID:SCR_001598) with E-value 1e–5 identified the rRNA fragments. The microRNA and small nuclear RNA genes were predicted with INFERNAL (INFERNAL, RRID:SCR_011809) [26] software against the Rfam database (Release 12) [27].

### Phylogenetic reconstruction of 10 vertebrate genomes

Protein-coding genes of Atlantic cod (*Gadus morhua*), tetraodon (*Tetraodon nigroviridis*), Antarctic notothenioid *N. coriiceps*, stickleback (*Gasterosteus aculeatus*), tilapia (*Oreochromis niloticus*), medaka (*Oryzias latipes*), zebrafish (*Danio rerio*), and mouse (*Mus musculus*) genomes were collected from Ensembl release 84 or NCBI, and *D. mawsoni* and *E. maclovinus* genes from this study, were used to build orthologous clusters with OrthoMCL version 2.0.9 (OrthoMCL, RRID:SCR_007839) [28] with default parameters and options. A total of 2,936 one-to-one single-copy genes were identified among the 10 species. Protein-coding sequences of the orthologs were aligned using PRANK (version 140603) [29] under a protein model with default parameters. The coding sequences of the genes were concatenated to a supergene for each species. The supergene sequence data set was subjected to phylogenetic analysis using MrBayes (MrBayes, RRID:SCR_012067) [30], implementing best-fit substitution model (GTR+gamma+I) as determined by Modeltest [31]. The analysis was run 800,000 generations, sampling every 100 generations, with the first 2,000-sample set as burn-in. Branch-specific *dN* and *dS* were estimated with codeml of the PAML package [32]. The analyses of changes in gene family size were computed with CAFÉ (CAFE, RRID:SCR_005983) [33].

### Gene ontology annotation and identification of positive selection genes

Gene ontology (GO) terms of the *D. mawsoni, E. maclovinus*, and stickleback orthologs were built with InterProScan (InterProScan, RRID:SCR_005829) [34]. The orthologs of each GO term were concatenated to estimate branch-specific *dN* and *dS* using codeml of PAML (PAML, RRID:SCR_014932). A binomial test was used to identify the excess of nonsynonymous changes of GO categories in either *D. mawsoni* or *E. maclovinus* lineages referenced to the stickleback. Only the GO terms carrying more than 30 orthologs were put into this calculation. To detect genes evolving under positive selection in *D. mawsoni*, we used the branch-site model in which likelihood ratio test *P* values were computed. Fisher exact tests were used to test for overrepresented functional categories among the positive select genes. GO enrichment analyses of the genes under positive selection were performed using a hypergeometric method.

### Calling of heterozygous single-nucleotide polymorphisms

All of the paired-end reads were mapped to the assembled scaffolds with the aligner SMALT (SMALT, RRID:SCR_005498) to detect the heterozygous sequence polymorphism in the genomes. The heterozygous single-nucleotide polymorphisms (SNPs) were called with SSAHA_Pileup (version 0.8; [35]). Five thresholds were used to post-filter unreliable SNPs: (i) SSAHA_Pileup SNP score ≥20, (ii) ratio of 2 alleles between 3:17 to 17:3, (iii) the lowest sequencing depth for each allele ≥5, (iv) the minimum distance for adjacent SNPs ≥5 bp, and (v) only 1 polymorphism detected at each SNP position.

### Transcriptome analyses

RNA-Seq data derived from liver, gill, stomach, white muscle, red muscle, skin, small intestine, brain, head kidney, caudal kidney, spleen, and ovary were analyzed for variations in gene expression of *D. mawsoni* and *E. maclovinus*. RNA-Seq reads were trimmed using Trimmomatic (Trimmomatic, RRID:SCR_011848) (version 0.33) [36] with the parameter set to AVGQUAL at 20, TRAILING at 20, and MINLEN at 50. The cleaned Illumina paired-end reads of each tissue were mapped to the annotated scaffolds of *D. mawsoni* and *E. maclovinus* genome using HISAT2 (HISAT2, RRID:SCR_015530) aligner (version 2.0.4) [37]. Cufflinks (Cufflinks, RRID:SCR_014597) (version 2.2.1) [38] normalized gene expressions to the quantified transcription levels (fragments per kilobase million). Differential expressions of the genes were assessed using DEGseq (DEGseq, RRID:SCR_008480) (version 1.28.0) [39] with cutoff at *q* < 0.001 [40]. GO and KEGG enrichment analyses for the identified differentially expressed genes (DEGs) were performed using cluserProfiler packages [41] with the cutoff at *P* < 0.05.

### Construction of gene collinearity among *D. mawsoni, E. maclovinus*, and stickleback genomes

The genes of *D. mawsoni* and *E. maclovinus* were aligned to the gene model set of stickleback by Blastp (BLASTP, RRID:SCR_001010) with E-value at 1e–20. Two criteria were used to call syntenic gene blocks in the *D. mawsoni* or *E. maclovinus* scaffolds: (i) number of the gene on the syntenic block ≥3 and (ii) number of nonsyntenic genes between 2 adjacent syntenic genes ≤10. Each syntenic block was anchored on the stickleback genomes according to the orders of the reference gene.

### Western blot analysis of zona pellucida proteins

The zona pellucida proteins (ZPs) were separated on 10% sodium dodecyl sulfate polyacrylamide gel electrophoresis (SDS-PAGE) at 100V for 90 min in 193 mM glycine and 25 mM Tris (pH 8.8). The resolved proteins were electrophoretically transferred to a nitrocellulose membrane (Millipore) using a Mini-Protean Tetra Cell (BioRad) in a buffer containing 193 mM glycine, 25 mM Tris (pH 8.3), and 20% methanol. The membrane was treated with blocking agent (5% nonfat milk in 1x Tris-buffered saline with Tween 20 [TBST]) for 2 h at room temperature on a shaker. FLAG antibody or β-actin antibody (Hua An Biotechnology Co. Ltd, Hangzhou, China) was added, and the membranes were incubated at room temperature for 1 h. The membrane was then washed with 1x TBST 3 times for 15 min each. The secondary antibody (1:2000 in 1x TBST, Boston Biomedical Inc.) was then added and incubated for 1 h at room temperature. The membrane was washed with 1x TBST twice and 1x TBS once for 15 min each. Color was developed using SuperSignal West Pico Chemiluminescent Substrate (Thermo Scientific) according to the manufacturer's instructions. Images were acquired using a ChemiDoc MP Imaging System (BioRad).

### Assay of Chinese hamster ovary cell survival rate at freezing temperature

DmZPC5 exons were serially deleted using polymerase chain reaction amplification of the DmZPC5 expression vector with primers designed to eliminate desired coding sequences (Fig. 3b). The full-length sequences of 3 DmZPC5 isoforms (DmZPC5-1, DmZPC5-2, and DmZPC5-3) were engineered to contain a FLAG octapeptide and cloned into the expression vector pIRES2-EGFP (Additional file 1: Fig. S5). The 3 constructed vectors and blank control (vector pIRES2-EGFP) were transferred into the Chinese hamster ovary (CHO) cells (American Type Culture Collection CCL-61) obtained from American Type Culture Collection. The CHO cells were cultured in Dulbecco's modified Eagle's medium containing 10% fetal bovine serum (Gibco). CHO cells were incubated at 37°C for 2 days and then kept at −2°C for 8 h. The treated CHO cells were collected and washed with Dulbecco's phosphate-buffered saline (PBS) twice. The cells were stained with 10 µg/mL propidium iodide at room temperature for 5 minutes, and numbers of propidium iodide−stained cells (dead cells) were determined by flow cytometry. The survival rate was calculated with the following equation: survival rate = *S*/(*S* + *D*), where *S* is the number of surviving cells and *D* is the number the dead cells.

### Identification of the long interspersed TEs and estimation of their divergence time

The seed sequences of the long interspersed TEs (LINEs) [42] were aligned against the *D. mawsoni* and *E. maclovinus* draft genomes, respectively, using BlastN at E-value of 1e-10. According to loci of the alignments, the sequences were extracted from the genomes, which were considered as the candidates of the LINEs. If distance between 2 adjacent candidates was ≤200 bp, these 2 candidates were connected by the sequence between them. All of the candidates and their corresponding seed sequences were mutually aligned by BlastN. Those candidates with >60% identity and >100 bp of alignments to the seed sequences were collected as the LINEs of *D. mawsoni* and *E. maclovinus*, respectively.

Alignment of the LINEs was conducted by the multiple sequence aligner ClustalW (ClustalW, RRID:SCR_002909) [43] to cluster any 2 LINEs with highest sequence similarity into a LINE pair in *D. mawsoni* or *E. maclovinus*. The evolutionary distance of 2 LINES for each LINE pair was calculated by the Kimura 2-parameter method (EMBOSS distmat, version 6.6.0.0), which reflected the substitution rate per site between the 2 LINEs. According to the calculated synonymous substitution rates for 7,958 *D. mawsoni*−*E. maclovinus* orthologous pairs, the mean synonymous substitution rate is ∼0.227. The peak of the substitution rate is at 0.04, which estimated the LINE burst to be ∼6.5 mya when the species divergence time between *D. mawsoni* and *E. maclovinus* is ∼37 mya [44].

### Tissue fixation and immunohistochemistry

Pieces of pelvic bone (with muscle) and the attached fins (≤20 × 20 × 5 mm) were dissected from frozen specimens of young *D. mawsoni* and *E. maclovinus* and immersed in a fixation solution, KINFix, which contains 62.5% (v/v) ethanol, 6.71% (v/v) acetic acid, and 6% (w/v) trehalose [45], for >24 h. Tissues were decalcified in ethylenediaminetetraacetic acid solution (cat. No. 041-22031, WAKO) for ∼2 weeks. Then the specimen was dehydrated in graded ethanol (70%, 80%, 90%, 95%, 1 × 1 h each), 100% ethanol 2 × 1 h at room temperature, xylene for 2 × 1 h, and embedded in low-melting paraffin for 2 × 1 h, and kept overnight at 56°C, then embedded in paraffin. For each tissue, 5-μm thick serial sections were cut with a microtome (RM2245, Leica). Immunostaining was performed using the EnVision detection system (cat. No. K5007, Dako). Slides were deparafinized in xylene and rehydrated in a descending series of ethanol (100%, 95%, 90%, and 70%), and washed in PBS. Endogenous peroxidases were blocked with 3% H_2_O_2_ for 10 min, after which the sections were incubated with 5% bovine serum albumin for 35 min. Then, the slides were incubated overnight with the primary connective tissue growth factor (CTGF) antibody (1:400 dilution) (cat. No. ab6992, Abcam) at 4°C. Next, the sections were washed 4 times with PBS for 15 min followed by incubation with a goat anti–rabbit secondary antibody for 35 min at 37°C. After 4 washes with PBS, 3, 3’-diaminobenzidine was added to visualize the immunoreactivity. All slides were counterstained with hematoxylin-eosin. The sections were dehydrated in a mounting series of alcohol (70%, 90%, 100%, and 100%) and in xylene. Finally, slides were mounted using neutral balsam mounting medium, and analyzed under a bright-field microscope (AXIO imager M2, ZEISS).

## Results

### *D. mawsoni* and *E. maclovinus* genome sequencing and assembly

**Figure 1:**
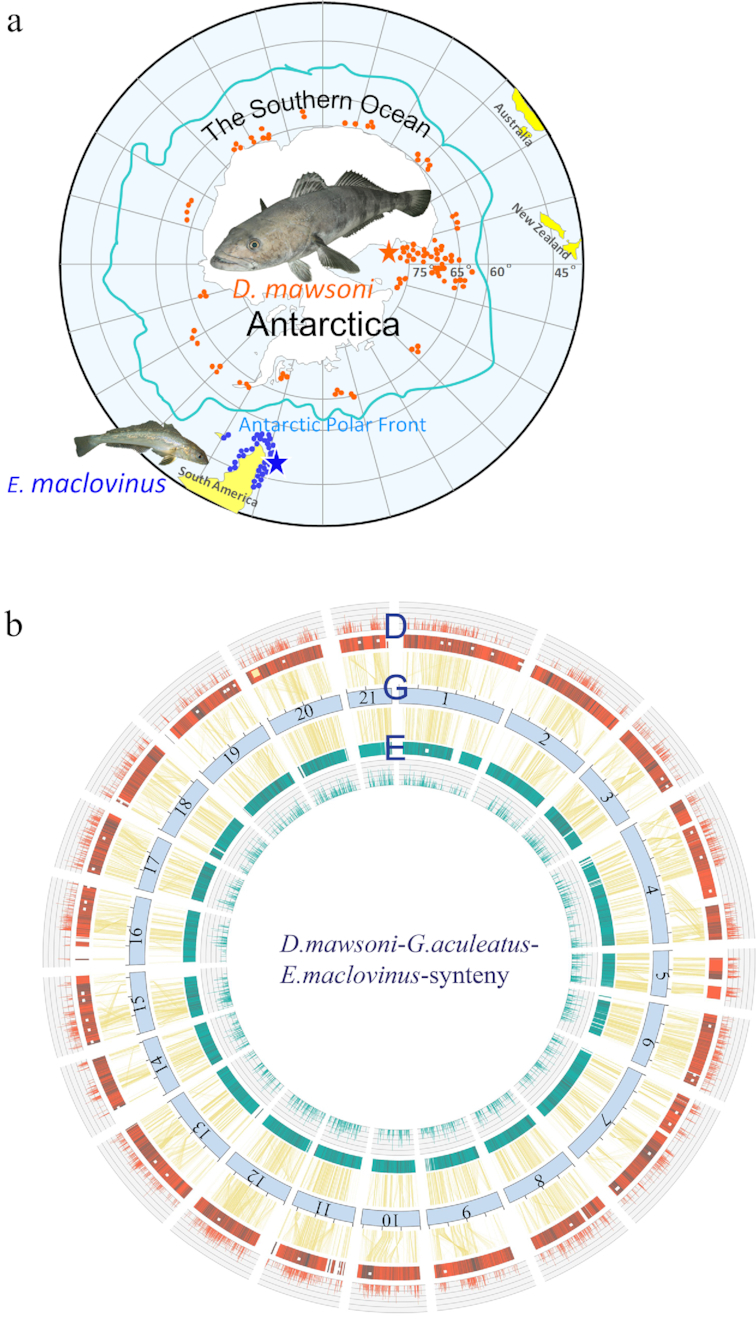
Sequenced species and genome synteny. (**A**) Sampling location and geographic distribution. The red and blue filled circles indicate the geographic distributions of *D. mawsoni* and *E. maclovinus*, respectively (www.fishbase.de version 02/2018 [50] and Hanchet et al. [51]). The red and blue stars show the respective locations where the sequenced individuals were collected. The Antarctic polar front, an approximation of the mean position of the Antarctic circumpolar current, is adopted from Barker and Thomas [52]. The image of *D. mawsoni* is a courtesy from Elliot DeVries and that of *E. maclovinus* is from Dirk Schories. (**B**) Gene collinearity among *D. mawsoni, E. maclovinus*, and *G. aculeatus* (stickleback). The scaffolds of *D. mawsoni* (the circularized red blocks labeled with “D”) and *E. maclovinus* (the circularized green blocks labeled “E”) are anchored on the 21 stickleback chromosomes (the circularized light blue blocks labeled with “G,” 1–21), according to the gene collinearity (the connecting yellow lines). The black vertical lines within the *D. mawsoni* and *E. maclovinus* scaffolds indicate occurrence of LINEs greater than 500 bp in these positions. The sequence length is indicated by the 5-Mb tick marks on the reference stickleback chromosomes. The outermost circle of red vertical lines and the innermost circle of green vertical lines indicate the quantified expression levels (fragments per kilobase million) of the genes located on the corresponding *D. mawsoni* and *E. maclovinus* scaffolds, respectively. The expression profiles are derived from the transcriptome data of white muscles (see the transcriptome section). The small white squares and rectangles scattered in the scaffolds show the locations of the selcys-tRNA genes of *D. mawsoni* and *E. maclovinus*. The single yellow square shows the location of AFGP genes in the *D. mawsoni* genome.

The geographic distributions and sampling locations of *D. mawsoni* and *E. maclovinus* are illustrated in Fig. 1a. The genome of 1 *D. mawsoni* juvenile (12 kg) of undetermined sex and 1 young adult male *E. maclovinus* (∼100 g) were *de novo* sequenced using Illumina sequencing platforms. The genomes of both species comprise 24 pairs of chromosomes (2*n* = 48) [46, 47]. Analyses of 17-mer frequency distribution indicated a genome size of approximately 842 Mb for *D. mawsoni* and 727 Mb for *E. maclovinus* (Additional file 1: Fig. S1), consistent with the mean genome sizes of 840 and 780 Mb for the toothfish and robalo, respectively, determined by flow cytometry (Additional file 1: Table S1a). The raw sequence data after cleaning and error correction (Additional file 1: Table S1b, S1c) were assembled using SOAPdenovo [17] for *D. mawsoni*, and Platanus [48] for *E. maclovinus* followed by scaffold building with SSPACE [18]. The assembled toothfish genome had a contig N50 length of 23.1 Kb and scaffold N50 length of 2.2 Mb, while those of the robalo were 10.9 Kb and 0.69 Mb, respectively. The assembled toothfish and robalo genomes are approximately 757 and 744 Mb, respectively (Table 1; Additional file 1: Table S2a, S2b), consistent with *k*-mer and flow cytometry estimates, and achieving >90% and 95% coverage of the genome size based on flow cytometry of the 2 species, respectively. The completeness of both genomes was assessed with benchmarking universal single-copy orthologs (BUSCOs) [49], referencing the lineage data set of actinopterygii_odb9 and orthologs of zebrafish, which reflected the complete BUSCOs at 97.2% for the *D. mawsoni* genome and 95.0% for the *E. maclovinus* genome (Additional file 1: Table S2c). The guanine-cytosine content of the *D. mawsoni* genome is 0.4070, nearly identical to the 0.4066 of *E. maclovinus*, and both are lower than that of a model fish, the stickleback *Gasterosteus aculeatus* (Additional file 1: Fig. S2). The accuracy of the genome assembly was assessed by alignment of the scaffolds to publicly available unigenes of *D. mawsoni* and *E. maclovinus*, and the coverage of the initial contigs was found to be approximately 98.8% and 99.1%, respectively (Additional file 1: Table S3), suggesting an acceptable quality of the genome assemblies. Alignment of the sequence reads to the assemblies estimated an overall heterozygous rate of approximately 2.58 and 2.40 per Kb for *D. mawsoni* and *E. maclovinus*, respectively (Additional file 1: Table S4).

**Table 1:** Overview of assembly and annotation

	*D. mawsoni*	*E. maclovinus*
Assembly		
Total length (Mb)	756.8	744.4
Contig N50 length (Kb)	23.1	10.9
Scaffold N50 length (Kb)	2,216.2	694.7
Scaffold N90 length (Kb)	202.7	167.2
Largest scaffold (Mb)	13.8	4.9
Quantity of scaffolds (>N90 length)	536	1,185
Annotation		
Quantity of predicted protein-coding genes	22,516	22,959
Quantity of predicted noncoding RNA genes	2,434	2,185
Content of TEs (%)	21.38	10.02
Heterozygous SNP rate (SNPs per kb)	2.58	2.40

### Genome annotation and synteny alignment between *D. mawsoni* and *E. maclovinus*

A total of 22,516 and 22,959 protein-coding genes were annotated in the *D. mawsoni* and *E. maclovinus* genome, respectively, by combining the results from homologous and *de novo* prediction methods using the gene modeler GLEAN (Additional file 1: Table S5). The protein-coding genes of the toothfish and robalo, along with the sequenced notothenioid *N. coriiceps* and model species *G. aculeatus* and zebrafish *Danio rerio*, were clustered using OrthoMCL [28]. We found 8,825 gene clusters that were common to all 5 species. Genes shared among the notothenioids are similar in quantity, 12,269 between toothfish and robalo and 12,421 between toothfish and *N. coriiceps* (Additional file 1: Fig. S3). In annotations of conserved noncoding RNA genes, we predicted 1,097 tRNA, 110 rRNA, 422 small nuclear RNA, and 295 microRNA genes in the toothfish genome (Additional file 1: Table S6a), while the robalo genome was annotated to carry 1,037 tRNA, 44 rRNA, 891 small nuclear RNA, and 286 microRNAs (Additional file 1: Table S6b). The much larger number of rRNA copies (2.5-fold) in *D. mawsoni* than *E. maclovinus* is consistent with the presence of dual chromosomal loci of recombinant DNA genes detected by *in situ* fluorescent hybridization in the giant toothfish [46], as opposed to the single recombinant DNA locus in other notothenioids [53]. The 2 species showed largely similar profiles in their microRNAome, with minor differences in the copy number of some individual microRNA (Additional file 1: Table S7). Strikingly, the toothfish genome contains many more selcys-tRNA genes than robalo (84 vs. 1; Additional file 1: Table S8). This extensive duplication of selcys-tRNA genes, accompanied by high expression of selenoproteins in *D. mawsoni* (detailed in a later section: "Transcriptomic adaptation to the cold environment"), signifies that mitigation of oxidative stress through selenoproteins, many of which are strong antioxidants, is likely an important selection force in the evolution of the Antarctic notothenioid genome in the freezing and oxygen-rich waters.

*De novo* annotation of repeat sequences revealed >2-fold increase in overall repeat content in *D. mawsoni* (21.38% of genome) over the basal *E. maclovinus* (10.02%) (Additional file 1: Table S9). TEs of the toothfish genome, including long terminal repeat retrotransposons, non–long terminal repeat retrotransposons (LINEs and short interspersed TEs [SINEs]), and DNA transposons, was more than twice that of *E. maclovinus*. Examination of the *N. coriiceps* genome exhibited similar accumulation of the TEs (23.5%). Among simple sequence repeats, dimer repeats constituted the majority in both genomes, and tetramers and pentamers showed the highest levels of increment in *D. mawsoni* (Additional file 1: Table S10a and S10b). The doubling of TE content in the *D. mawsoni* and *N. coriiceps* genomes suggests higher activity of TEs in the Antarctic species relative to the basal robalo, suggesting a likely contributing factor to the observed trend of increasing genome sizes in more derived Antarctic notothenioid lineages [54].

For global genome alignment, we anchored the *D. mawsoni* and *E. maclovinus* scaffolds to the 21 linkage groups of the well-characterized 3-spined stickleback genome according to gene collinearity (Fig. 1b; Additional file 2). Most of the notothenioid scaffolds had extensive collinearity to the corresponding stickleback chromosomes. The 84 duplicated selcys-tRNA genes are widespread throughout the toothfish genome. Inversion and translocation of genome segments have occurred in both *D. mawsoni* and *E. maclovinus* relative to stickleback, but the *D. mawsoni* genome showed more frequent rearrangements. Insertion sites for the expanded TEs of the *D. mawsoni* genome were random, thereby expanding the lengths of almost all of the linkage groups. Mapping the RNA-Seq transcript data that we obtained from white muscles for the 2 species on their respective synteny showed that the heavier insertion of TEs in the toothfish genome did not appear to adversely affect the expression of the neighboring protein genes because strong gene expression in regions of high TE content was maintained (Fig. 1b).

### Burst of LINE expansion in the cold

**Figure 2:**
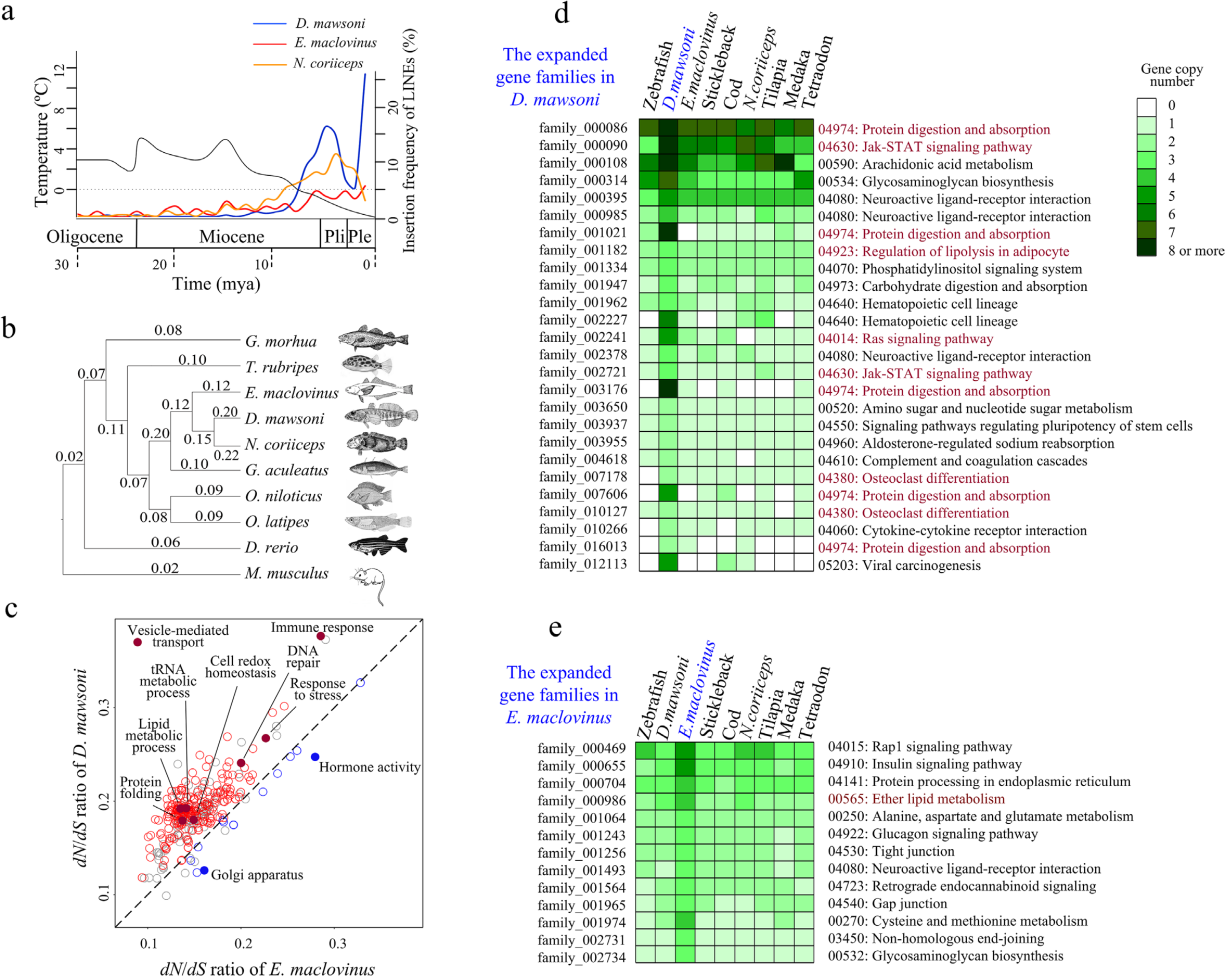
Evolution of the genomes and genes. (**A**) Timing and frequency of LINE insertion in *D. mawsoni, E. maclovinus*, and *N. coriiceps* showing correlation between onset of late Miocene deep cooling and burst LINE insertions in the Antarctic toothfish and bullhead notothen. The black trace indicates global temperature trends during the Oligocene, Miocene, Pliocene (Pli), and Pleistocene (Ple) from 30 to 0 mya, modified from Zachos et al. (2008) [58], Near et al. (2012) [9], and Favre et al. (2015) [59]. The red and blue lines indicate the insertion frequency of LINEs (the percentage of the calculated LINE pairs) in the *D. mawsoni* and *E. maclovinus* genomes, respectively, during these periods. **(B)** Reconstructed phylogeny of 9 teleost fish lineages using 2,936 orthologous genes (mouse serving as outgroup) and the calculated *dN*/*dS* ratio for each branch, showing a 2-fold faster evolutionary rate in the Antarctic notothenioids. (**C**) Comparison of adaptive evolution between *D. mawsoni* and *E. maclovinus* genomes. Data points represent the mean *dN*/*dS* value of each GO term, each of which consists of ≥30 genes. The red and blue circles show the GO terms with significantly higher *dN*/*dS* ratios (*P* < 0.05, binomial test) in *D. mawsoni* and *E. maclovinus*, respectively. The grey circles are those showing no significant difference. GO terms falling on the dashed line of linearity have the same *dN*/*dS* ratios in the 2 species. (**D**) Gene duplication in *D. mawsoni*. A subset [56] of the 202 gene families detected to contain higher gene copy numbers in the *D. mawsoni* genome relative to other species are listed on the left, with their respective KEGG pathway listed on the right. The gene copy numbers are measured by color difference. The pathways highlighted in red are especially abundant in *D. mawsoni* and might be relevant to physiological adaptation of *D. mawsoni* in the freezing environment. (**E**) A subset of duplicated gene families in *E. maclovinus*, showing different KEGG pathways between *D. mawsoni* and *E. maclovinus* in terms of gene duplication. The red highlighted pathway (ether lipid metabolism) indicates that a common duplication occurred in the 3 notothenioids.

Involvement of gain (or loss) of mobile element copies in genome size and genome restructuring affecting species differentiation has increasingly gained empirical support [55, 56]. TEs are normally under epigenetic regulation, but waves of TE proliferations could arise from environmental changes that cause physiological stress and disrupt epigenetic control [57]. We therefore examined potential linkage in timing between LINE expansion (2-fold increase in the toothfish vs. robalo) and onset of frigid SO marine conditions. A multiple sequence alignment of several LINEs (LINE/I, LINE/L2, LINE/RTE-BovB, and LINE_Rex-Babar—the most abundant types in the toothfish genome) was made, which clustered 349 *D. mawsoni* LINE pairs and 213 *E. maclovinus* LINE pairs, where the 2 LINEs within each pair share the highest sequence similarity, approximating the least divergence time. Calculation of the nucleotide substitution rates for each LINE pair identified a burst of emergence of LINEs in the *D. mawsoni* genome with the substitution rates centered at 0.04, which corresponded with a divergence time of 6.5 mya (Fig. 2a; see also Materials and Methods) based on the mean substitution rate of the LINEs. A similar trend of LINE expansion was also observed in the *N. coriiceps* genome. This estimated timing of LINE burst expansion correlated with the radiation of the majority of the modern Antarctic notothenioid clades beginning in the late Miocene when seawater temperatures steadily declined [9]. In contrast, no burst expansion of LINEs was detected in the *E. maclovinus* genome, supporting an Antarctic/cold-specific LINE burst in the toothfish, and corroborating our prior empirical evidence for an increase in retrotransposition activity of *D. mawsoni* LINE-1, resulting in more copies in the transfected cells when subjected to stress from nonphysiologically low incubation temperature [42].

### Accelerated protein evolution in the cold

Ectotherms are vulnerable to disruptive effects on protein structures and reaction rate depression at low temperatures. Antarctic notothenioids in perennially freezing high-latitude waters face the extremes of these effects, as well as an oxidative environment due to high oxygen concentrations resulting from increased gas solubility at low temperatures. We examined evidence for adaptive evolution of proteins in response to these selection pressures. The gene models of *D. mawsoni, E. maclovinus*, 7 other teleost genomes, and the mouse genome as outgroup were clustered, from which 2,936 one-to-one single-copy orthologs were obtained to reconstruct a phylogenetic tree (Fig. 2b). Stickleback is the closest species to the notothenioid clade as expected, and *E. maclovinus* is sister to the 2 Antarctic notothenioids, *D. mawsoni* and *N. coriiceps*. We found that evolutionary rates of the orthologous genes based on calculated *dN*/*dS* values (the ratio of the rate of nonsynonymous substitution to the rate of synonymous substitution) were elevated in the notothenioid lineage compared with the other fish lineages. This faster rate is more pronounced in the 2 Antarctic species, about twice that of the basal *E. maclovinus*, suggesting intensified selection pressures driving genome evolution in the Antarctic environment. To identify GO categories that were evolving faster in the toothfish or robalo, the *dN*/*dS* ratios of 7,584 orthologous genes among the 2 notothenioids and the stickleback were calculated and the mean *dN*/*dS* value of the genes associated with each GO term was calculated for each species. These orthologous genes were annotated to 411 GO terms, of which 281 showed significantly higher mean *dN*/*dS* ratios in *D. mawsoni*, while only 19 demonstrated higher mean rates in *E. maclovinus* (Fig. 2c; Additional file 2). The faster evolving GO processes in *D. mawsoni* included "gene expression," "protein folding," "tRNA metabolic process," "cell-redox homeostasis," "immune response," "response to stress," "lipid metabolic process," "DNA repair," "vesicle-mediated transport," and others. To assess which *D. mawsoni* genes experienced positive selection, we tested using the Branch-site model (in PAML) on a reconstructed phylogenetic tree of 6 fish species with the *D. mawsoni* lineage assigned as the foreground branch (Additional file 1: Fig. S4a). A total of 526 positively selected genes were identified (Additional file 1: Table S11a). The most significantly enriched KEGG pathway (Additional file 1: Table S11b) was “protein digestion and absorption” (*P* = 5.1e−5) and GO term (Additional file 1: Table S11c) was “protein binding” (*P* = 6.0e−4), which indicate that maintenance of protein homeostasis played an important role in shaping the *D. mawsoni* genome. In addition, a complement of genes (*sorbs2a, acox1, apoa1a, scp2a, tnni3k*, and perilipin-like genes; Additional file 1: Table S11a) involved in the peroxisome proliferator-activated receptor (PPAR) signaling pathway—the key pathway in adipocyte development regulation—were found to be under positive selection, suggesting occurrence of adaptive changes in lipid metabolism in *D. mawsoni*.

### Gene duplication in the freezing environment

Gene duplication plays fundamental roles in the emergence of adaptive features. In the list of predicted protein coding genes from the toothfish and robalo genomes, we identified 202 families that have increased in copy number in *D. mawsoni*, compared with the other 8 fish genomes (Additional file 3). KEGG enrichment analyses of these expanded gene families yielded enrichment in pathways involved in protein homeostasis and lipid and bone metabolism, such as “protein digestion and absorption,” “regulation of lipolysis in adipocyte,” “fat digestion and absorption,” “ether lipid metabolism,” and “osteoclast differentiation” (Fig. 2d; Additional file 2), suggesting that genomic capacity for these functional pathways had increased in *D. mawsoni* during evolution in chronic cold conditions. Corroborating this cold-specificity is that the expanded gene families found in the basal *E. maclovinus* relative to the other fish genomes, including *D. mawsoni* and *N. coriiceps*, yielded distinctly different enriched KEGG profiles (Fig. 2e); this analysis indicates that the functional traits gained through gene duplication in *E. maclovinus* were driven by different selective pressures, consistent with our previous findings [60, 61]. Due to inherent inefficiency in correctly assembling highly similar DNA sequences in the shotgun sequencing strategy, there are likely many more duplicated genes that had eluded detection. For example, many paralogs of ZPs, such as ZPAX1, ZPC1, and ZPC2 in *D. mawsoni* have been shown, through array-based genome hybridization and quantitative polymerase chain reaction, to be duplicated [60, 62]. Among the set of genes found duplicated in our previous report [42], 23 are identified in this study (Additional file 1: Fig. S4b).

Gene duplication has contributed prominently to the evolutionary gain of freezing avoidance in Antarctic notothenioids. Generally regarded as a key innovation of the Antarctic notothenioid radiation, the AFGP gene evolved from a trypsinogen-like protease ancestor followed by extensive intragenic and whole-gene duplications, generating a large gene family that would provide an abundance of this novel life-saving protein [7]. By referencing the published AFGP haplotypes that were assembled from bacterial artificial chromosome clone sequences of the same individual used in this study (Genbank accessions HQ447059 and HQ447060) [63], we identified and assembled the AFGP loci shotgun reads, recaptured the 2 AFGP haplotypes and integrated them in the draft genome, and localized them to a region syntenic with a scaffold in LG 20 of the stickleback genome (Fig. 1b).

**Figure 3:**
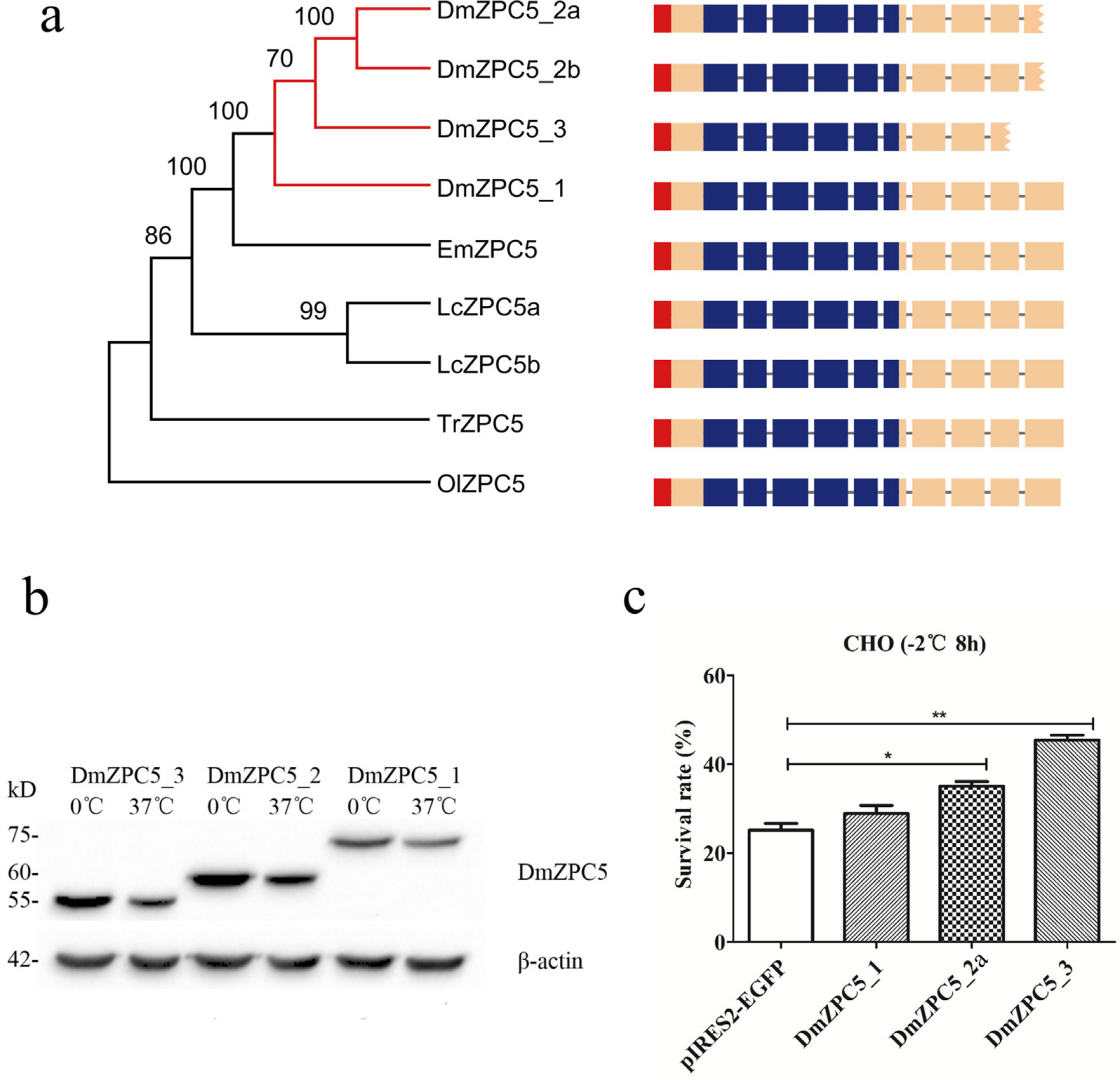
Evolutionary and functional analyses of the *DmZPC5* genes involved in cellular freezing resistance. (**A**) Duplication of *ZPC5*gene (DmZPC5) in *D. mawsoni*. Phylogenetic neighbor-joining tree of *ZPC5* genes among *D. mawsoni, E. maclovinus, Larimichthys crocea* (Lc), *T. rubripes* (Tr), and *O. latipes* (Ol). The gene structures are illustrated on the right. The different colored blocks indicate the exons encoding signal peptides (red), zp domains (blue), and the remaining exons (incarnadine). The jagged blocks contain the nonsense mutations in *DmZPC5-2a/b* and *DmZPC5-3* genes that cause premature termination of coding sequences. (**B**) Western blot analysis of the DmZPC5 isoforms indicated their sizes and temperature-sensitive accumulation. Purified proteins encoded by the 3 DmZPC5 isoforms were detected by an anti-FLAG antibody on the SDS-PAGE gels. All of these 3 DmZPC5 proteins had higher expression levels at 0°C than at 37°C. (**C**) Assays of cell survival rate under recombinant expression of different DmZPC5 isoforms in CHO cells at a freezing temperature (−2°C for 8 h). The bars represent the mean ± SD (*n* = 3, biological replicates). The sample pIRES2-EGFP is the expression vector as control. Significant differences in survival rate are indicated by the asterisk (unpaired Student t-test, *P* < 0.05) and double asterisk (*P* < 0.01).

Resistance to freezing extends beyond the AFGPs. We found gene duplication for a protein that *a priori* would not be expected to function in freeze resistance: ZP protein (or eggshell protein) additionally provided protection against cellular freezing. Products of *ZPC5* from *D. mawsoni* have been shown to enhance freezing resistance of eggs of recipient zebrafish both *in vivo* and *in vitro* [62]. In this study, we found 4 copies of *ZPC5* in the *D. mawsoni* genome that encoded 3 different sized ZPC5 proteins in the toothfish ovary—DmZPC5_1, DmZPC5_2a/DmZPC5_2b, and DmZPC_3 in decreasing order of size, corresponding to gradually shortened C-termini from the conserved ZP domain due to nonsense mutations in exons 9 and 10 (Fig. 3a; Additional file 1: Fig. S5a). A single *ZPC5* gene in the basal *E. maclovinus* was found, which corresponded to the full-length DmZPC5_1. We expressed the three DmZPC5 isoforms in CHO cells (Additional file 1: Fig. S5b and 3b) and assayed cell survival rate at freezing temperature (−2°C for 8 h). DmZPC5_3 was the most active isoform in maintaining cell viability while DmZPC5_1 was the least active one (Fig. 3c). Further analyses showed that DmZPC5_3 was more likely retained inside the cell than DmZPC5_2 and DmZPC5_1 (Additional file 1: Fig. S6), and less likely to become polymerized inside the cell compared with DmZPC5_1 (Additional file 1: Fig. S7a), corroborating our previous finding that only unpolymerized ZP proteins are active for the ice-melting promoting activity [62]. We detected DmZPC5 expression across many tissues besides ovary in *D. mawsoni* (Additional file 1: Fig. S7b), consistent with a distribution expected of a general protective function.

### Transcriptomic adaptation to the cold environment

**Figure 4:**
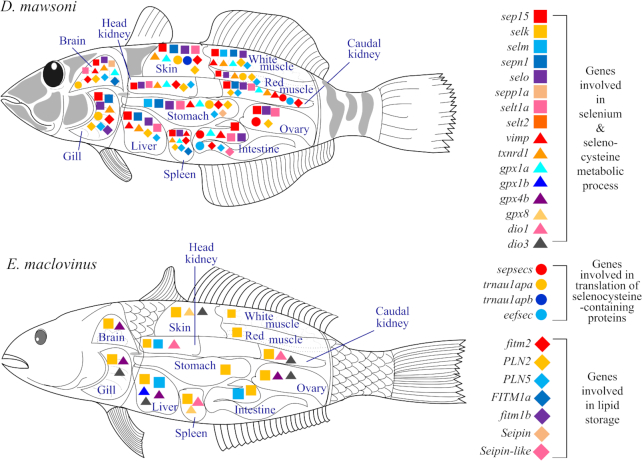
Comparison of gene expression between *D. mawsoni* and *E. maclovinus* tissues. The colored symbols represent the genes involved in 3 metabolic processes (listed on the right). The genes with significantly higher expression in *D. mawsoni* or *E. maclovinus* are labeled on the corresponding organs.

To assess the functional relevance of the detected genomic outcomes to life in freezing conditions, we characterized and compared transcriptomes of 12 tissues including brain, liver, red muscle, white muscle, gill, skin, intestine, stomach, spleen, head kidney, caudal kidney, and ovary between native specimens of *D. mawsoni* and *E. maclovinus*. We found that >10,000 genes were differentially expressed in pairwise comparisons between the 2 species, with the toothfish showing substantially more upregulated genes than the robalo. Enrichment testing on KEGG pathways yielded findings that many signaling pathways in the tissues of toothfish were significantly enriched in DEGs, including the transforming growth factor β (TGF-β), AMP-activated protein kinase, and PPAR pathways, known to play essential roles in development, metabolism, and stress responses (Additional file 1: Fig. S8a). GO enrichment analysis of the DEGs demonstrated upregulation of hundreds of GO biological processes, including translation, transferrin transport, cell redox homeostasis, cellular response to unfolded protein, ubiquitin-dependent protein catabolic process, regulation of innate immunity, MAPK cascade, positive regulation of apoptosis pathways, many of the pathways involved in lipid metabolism, and anti–reactive oxygen species pathways represented by “selenium compound metabolic process” and “selenocysteine metabolic process” (Additional file 1: Fig. S8b). These results corroborated the expression profiles in a previous study with the lower depth of sequencing available at that time [60], and additionally revealed further details of transcriptomic cold adaptation from the much deeper sequencing across a comprehensive set of tissue transcriptomes. A striking finding was the greatly increased transcriptional activities across many selenium-containing protein genes in the toothfish tissues compared with robalo (Fig. 4; Additional file 1: Fig. S9). Correspondingly, the genes involved in the translation of selenocysteine-containing proteins were also significantly upregulated in the toothfish (Fig. 4). Selenoproteins have well-known functions in coping with cellular oxidative stress; thus, the great expansion of selcys-tRNA genes (Fig. 1b, Additional file 1: Table S8) and the significantly upregulated expression of many kinds of selenoprotein mRNAs indicate that augmented anti–reactive oxygen species capacity has evolved as an important adaptation to the constantly freezing environment, where saturated levels of O_2_ and cold-depressed metabolic rates would make oxidative stress a formidable challenge for cellular life. Interestingly, the expression of glutathione peroxidase 4b (gpx4b) (Fig. 4), a selenoprotein uniquely able to reduce lipid hydroperoxides [64, 65], was lower in *D. mawsoni*, suggesting that alternative lipid metabolic programs may exist in the toothfish. Accordingly, we found that all isoforms of the major players in lipid droplet assembly (PLN2, PLN5, fitm, seipin) important for lipid storage in adipose tissue were upregulated in all toothfish tissues examined relative to *E. maclovinus*, signifying a shift of lipid distribution towards storage in *D. mawsoni*, as described in detail below.

### Altered lipid metabolism in *D. mawsoni* for neutral buoyancy

In a striking evolutionary departure from the heavy, bottom-water ancestral character (exemplified by *E. maclovinus*), a handful of Antarctic notothenioids have secondarily acquired neutral or near neutral buoyancy, enabling ecological diversification into and filling of mid-water niches—a distinctive hallmark of the Antarctic notothenioid adaptive radiation. The giant toothfish *D. mawsoni*, despite growing to massive sizes, being robustly muscled, and lacking a swim bladder, is the only notothenioid that has attained complete neutral buoyancy [14, 15]. Known morphological specializations include extensive lipid (mostly triglycerides) deposits under skin and in the musculature, and a light skeleton of mostly cartilage and little mineralized bone, adaptations that reduce overall density and provide static lift [14]. To understand the genetic basis of the large accumulation of lipids and reduced mineralization in *D. mawsoni*, we carried out transcriptome comparisons between *D. mawsoni* and several other notothenioids in which neutral buoyancy is not developed.

We compared gene expression profiles of muscles of *D. mawsoni*, and of the negatively buoyant Antarctic *N. coriiceps* and the basal *E. maclovinus* to elucidate evolutionary differences in the mechanisms of intermuscular lipid deposit. Compared with *E. maclovinus*, genes involved in triacylglycerol synthesis in the toothfish muscle were overrepresented in DEGs (*P* < 0.05) and markedly upregulated in transcription, including the key enzymes acylglycerol-3-phosphate O-acyltransferase isoforms and cytidine diphosphate-diacylglycerol synthase (Additional file 1: Fig. S10). An important regulator of this process, lipin1, was downregulated in *D. mawsoni*. Lipin1 is known to exert dual effects on lipid metabolism—it acts as a phosphatidate phosphatase enzyme to form diacylglycerol required for lipid synthesis but also serves as a transcriptional co-activator to promote fatty acid oxidation [66]. The downregulation of lipin1 was consistent with downregulation of fatty acid oxidation in *D. mawsoni* because about half of the genes involved in fatty acid oxidation were also downregulated relative to the robalo (Additional file 1: Table S12). At the same time, genes involved in regulation of lipid storage were overrepresented in the DEGs (*P* < 0.05) and all but 1 gene (*MEST*) were upregulated in the toothfish (Additional file 1: Table S13). These data strongly suggest a shift of metabolic pathways from lipid breakdown to lipid biosynthesis and lipid storage in *D. mawsoni* muscle relative to *E. maclovinus*, favoring deposit of lipids, thus contributing to neutral buoyancy. Compared with *N. coriiceps, D. mawsoni* muscle showed an overall trend of upregulation of genes involved in glycerolipid biosynthesis and lipid storage, but the differences are not as dramatic as in the *D. mawsoni/E. maclovinus* comparison. Expression levels of many genes relevant to lipid oxidation were fairly similar between the 2 species, suggesting common downregulation in lipid oxidation in the Antarctic species compared with the temperate *E. maclovinus* (Additional file 1: Fig. S10 and Tables S12 and S13). Consistent with downregulation of the lipid oxidation in the muscles of the 2 Antarctic fishes were their lower expression levels of lipid oxidation mitigating selenoenzyme gpx4 compared with the robalo. In total, the transcriptome comparisons revealed substantial genetic reprogramming in *D. mawsoni* muscle that would favor the large lipid deposition in this species.

**Figure 5:**
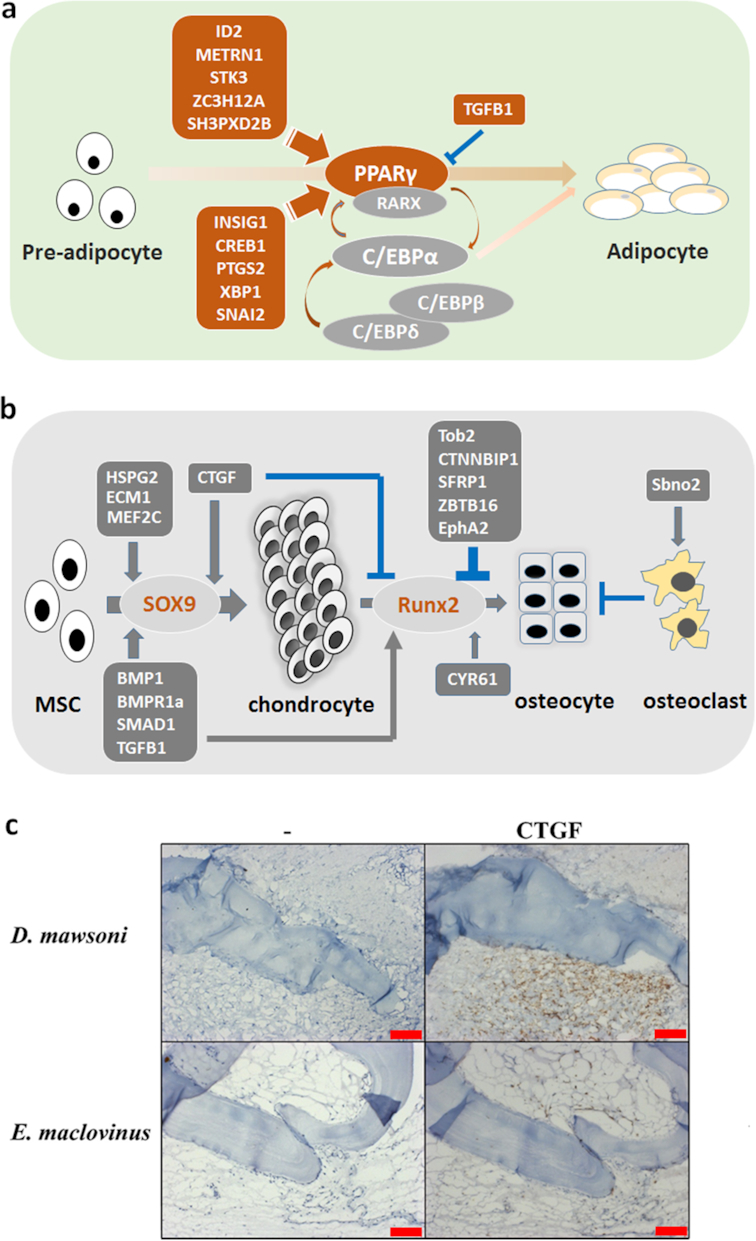
Schematic diagram showing changed regulation of buoyancy-related developmental pathways. (**A**) Enhanced adipogenetic pathways in *D. mawsoni* muscle. The genes shadowed in red were upregulated in *D. mawsoni* while those shadowed in grey were unchanged. (**B**) Changed osteogenetic regulation in *D. mawsoni* bone. Genes shadowed in dark grey were upregulated in *D. mawsoni* while those in light grey were not changed. The arrows (in dark red or dark grey) indicate a positive effect on the process while blocked (in blue) lines indicate inhibitory effect. MSC: mesenchymal stem cell. (**C**). Immunohistochemical staining to detect the abundance of CTGF in cross sections of pelvic fin of *D. mawsoni* and *E. maclovinus*. The left panels of each fish are immunohistochemical staining without the first antibody as negative control. The presence of CTGF is indicated by the brown staining in the tissues shown at right. Scale bar, 50 μm.

GO enrichment tests on the muscle DEGs also indicated regulatory change (*P* = 0.073) in fat cell differentiation between *D. mawsoni* and *E. maclovinus*. Adipogenesis in almost all animals is predominantly regulated by PPARγ [67]. Expression of PPARγ in *D. mawsoni* muscle was upregulated by more than 5.6-fold compared with *E. maclovinus* (Additional file 1: Table S14). In addition, as many as 16 known pro-adipogenetic factors were upregulated from 1- to 8-fold. Several factors in the TGF-β (TGFB1, smad3), Wnt (Sirt1, sirt2, frizzled-related protein [FRZB]), and Notch (jag1b, Hes1) pathways and Jun dimerization protein 2 (JDP2), reportedly negative regulators of adipogenesis, were also upregulated. When compared with *N. coriiceps*, about half of the DEGs in the *D. mawsoni/E. maclovinus* comparison under this GO term were statistically insignificant, but remarkably the comparison yielded significant upregulation of 10 pro-adipogenetic factors in *D. mawsoni* muscle, including the most important regulator, PPARγ (Fig. 5a), and only 1 negative regulating factor (TGFB1) [68, 69]. These results strongly suggest that regulatory promotion of adipogenesis in *D. mawsoni* muscle is a key contributing factor to fat deposition and attainment of neutral buoyancy.

### Reduction of ossification in *D. mawsoni*

To reveal the genes involved in the reduced bone ossification in *D. mawsoni*, we compared the transcriptomes of pelvic girdle bones between *D. mawsoni* (0% body weight in seawater) and the negatively buoyant Antarctic notothenioids *Trematomus bernacchii* and *Pagothenia borchgrevinki* (3.52% and 2.75% of body weight, respectively, in seawater) [14]. A total of 1,733 DEGs showing the same direction of change in the *D. mawsoni/P. borchgrevinki* and the *D. mawsoni/T. bernacchii* comparisons were identified and used for further analysis (Additional file 3). We found that 48 genes encoding various ribosomal proteins were significantly reduced in expression in the *D. mawsoni* bone, suggesting either a lower protein translation activity or fewer metabolically active cells in the pectoral girdle than in the other 2 nototheniids. The DEGs were enriched with hundreds of GO biological processes, including “extracellular matrix,” “ossification,” “response to hypoxia,” “angiogenesis,” and “lipid storage,” indicating that multiple genetic programs were distinctly regulated in the toothfish bone (Additional file 1: Fig. S11). In terms of ossification, it was noteworthy that expressions of the 2 major regulators of vertebrate bone development, *sox9* and *runx2* [70], were not significantly altered among the 3 notothenioids. However, expression of many genes of the BMP pathways, wnt pathways, and many regulatory factors known to be involved in the process were specifically altered in *D. mawsoni*, which likely shifted the developmental balance between chondrogenesis and osteogenesis (Fig. 5b; Additional file 1: Table S15). Among these highly upregulated genes (depicted in Fig. 5b), CTGF has been implicated in early events of osteogenic differentiation including proliferation and recruitment of osteoprogenitors; however, when expressed constitutively, CTGF would inhibit both Wnt-3A and BMP-9–induced osteoblast differentiation [71]. HSPG2 (prostaglandin-endoperoxide synthase 2) is required for the chondrogenic and adipogenic differentiation from synovial mesenchymal cells via its regulation of sox9 and PPARγ, but not for osteogenic differentiation via *runx2* [72], and ECM1 (extracellular matrix protein 1) interacts with HSPG2 to regulate chondrogenesis [73]. MEF2C, a transcription factor that regulates muscle and cardiovascular development, controls bone development by activating the genetic program for chondrocyte hypertrophy [74]. Some of the upregulated genes are known to inhibit osteoblastogenesis, such as Tob2 [75], CTNNBIP1 [76], secreted frizzled-related protein 1 (SRFP1) [77], and ZBTB16 [78]. Some members of the TGF-β superfamily (BMPR1a, TGF-β1, SMAD1), which were upregulated in *D. mawsoni*, are known to promote both chondrogenesis and osteoblastogenesis [79]. CYR61 and PTN specifically promote osteoblastogenesis [80, 81], but expression of PTN is drastically reduced, consistent with reduced hard bone formation. A few genes influence ossification via regulating osteoclastogenesis; e.g., Sbno2 promotes osteoclast fusion [82], and activation of the EphA2 signaling on osteoblasts led to bone reabsorption [83]. We found that genes associated with osteoclast differentiation are significantly enriched in the DEGs (*P* < 0.05) and all were upregulated (Additional file 1: Tables S16 and S17). Overall, the gene expression patterns in the toothfish bone demonstrated a genetic shift to chondrogenesis over osteoblastogenesis in bone development, which would reduce bone density and contribute to achieving neutral buoyancy.

Studies have indicated that the majority of clinical conditions associated with human bone loss are accompanied by increased marrow adiposity, possibly due to shifting of the balance between osteoblast and adipocyte differentiation in bone marrow stromal (skeletal) stem cells [84]. A few signaling pathways such as the TGF-β/BMP pathways and the Wnt pathway (represented by CTNNBIP and SFRP1 in this case) are known to participate in regulation of both bone and adipocyte development in animals. In the toothfish, we found enriched GO terms relevant to regulation of “response to lipid” and “lipid storage” (Additional file 1: Table S12), indicating possible linkage in the regulatory network that orchestrates the loss of ossification and gain of lipids in *D. mawsoni* bones.

To verify whether the elevated transcription of the regulatory factors indeed resulted in more abundant protein, we selected the factor CTGF for immunohistochemical staining in the bone and surrounding tissues of pelvic fins of *D. mawsoni* and *E. maclovinus* because it is the only factor for which an effective monoclonal antibody is currently available. Much stronger signal was detected in the *D. mawsoni* fin tissue (Fig. 5c), supporting a correlation between protein abundance and mRNA transcription in the case of CTGF. This result further supports the involvement of CTGF in the reduced ossification in *D. mawsoni*.

## Discussion

We sequenced and compared the genomes and transcriptomes of the cold-adapted high-latitude Antarctic toothfish *D. mawsoni* and the basal temperate relative *E. maclovinus* representing the ancestral character state to deduce Antarctic-specific evolutionary and adaptive changes supporting physiological activities of notothenioid fishes in freezing and oxygen-rich SO waters, as well as the gain of secondary pelagicism fundamental to Antarctic notothenioid niche expansion and adaptive radiation. The assembled genomes achieved 90% (*D. mawsoni*) and 95% (*E. maclovinus*) coverage of the respective genome size estimated by cell flow cytometry, and with greater scaffold N50 than the currently available sole Antarctic notothenioid (*N. coriiceps*) genome, greatly enhancing comprehensive, genome-wide discovery of evolutionary processes.

We found 2-fold expansion of TEs in the Antarctic toothfish over the temperate robalo *E. maclovinus* and deduced the timing of a burst of 1 major class of TEs (LINEs) to ∼6.5 mya, temporally correlating with the late Miocene onset of a steady cooling trend of the SO and diversification of the modern Antarctic notothenioid clade, suggesting a role of cold-induced TE expansion in notothenioid speciation. We found that many of the protein coding genes in the toothfish evolved rapidly and experienced positive selection, among which genes relevant to preservation of protein homeostasis were particularly prominent. Multiple gene families have undergone duplication during evolution in the cold, as exemplified by genes that confer resistance to freezing in the cold SO waters: the AFGP family that evolved *de novo* and confers extracellular freeze avoidance, and duplicated ZP ZPC5 genes that functionally diversified to aid in cellular freezing resistance. Through transcriptome comparisons, we found that functional output of the cellular apparatus for selenoprotein production in the Antarctic toothfish was greatly elevated compared with the basal temperate robalo, suggesting evolutionary mobilization of antioxidant selenoproteins in mitigating intensified oxidative stresses arising from the O_2_-rich SO environment.

The evolutionary transition from the negatively buoyant ancestral character to complete neutral buoyancy in the Antarctic toothfish entailed remarkable genetic reprograming of fat deposition and bone development. We found upregulation of processes of adipogenesis in skeletal muscle, and triacylglycerol synthesis and fat storage were favored over fatty acid oxidation. In bone development, a regulatory cascade favoring chondrogenesis over osteoblastogenesis was especially evident. The shift in fat synthesis and storage, together with reduction of ossification, is therefore key in evolutionary gain of neutral buoyancy and secondary pelagicism in *D. mawsoni*, and likely in the handful of other pelagic notothenioids, allowing them to diversify into mid-water niches, a distinctive hallmark of the Antarctic notothenioid adaptive radiation.

The remarkable diversification of Antarctic notothenioids (and several other polar fish lineages) is integral to the conclusion from a recent analysis of latitudinal diversity gradient of marine fishes that high-latitude cold water lineages exhibit exceptionally high rates of speciation compared with tropical lineages, counter to expectation based on latitudinal species richness [85]. Rates of molecular evolution based on phylogenetic tree branch lengths are not found to be slower at high latitudes [85]. We have shown more definitively in this study that in the cold-adapted Antarctic notothenioid fish, evolutionary rates in fact accelerated in thousands of protein-coding genes; extensive cold-specific gene duplication and functional diversification had occurred, such as in the ZP protein gene families; and TE mobility was remarkably elevated, which likely contributed to the observed higher frequency of chromosomal rearrangements. In mammals, ZP3 is known to function in sperm–egg recognition [86] and TE activity is positively related to rate of speciation [87]. How these genomic and functional changes elicited by selective pressures from the cold SO temperatures might have acted as intrinsic factors affecting notothenioid speciation are rich questions for further investigation.

## Conclusion

In summary, the results of this study provided robust new insights into genomic and transcriptomic alterations enabling cold adaptation and niche expansion of the predominant and ecologically vital Antarctic fish group in the SO. The genomes also serve as valuable resources for future investigations of genomic and evolutionary changes in the diverse Antarctic notothenioid families driven by paleoclimate changes in the SO, studies that may shed light on questions of why the coldest ocean has been a hot spot of species formation.

## Availability of supporting data and materials

All of the Illumina short read sequencing data of this project have been deposited at NCBI under the accession No. BioProject PRJNA401363 (http://www.ncbi.nlm.nih.gov/sra/). The assembled draft genomes and their annotations have been released at the official website of the Shanghai Ocean University (http://202.121.66.128/). The current version of the data set is the first version (v1). Antarctic toothfish genome and transcriptome [88], Patagonian robalo genome and transcriptome [89], and other supporting data are also available via the *GigaScience* GigaDB repository [90].

## Supporting information

**URLs**: KEGG, http://www.genome.jp/kegg/; KAAS, http://www.genome.jp/tools/kaas/;SSPACE, https://www.baseclear.com/genomics/bioinformatics/basetools/SSPACE; Platanus, http://platanus.bio.titech.ac.jp/; SMALT, http://www.sanger.ac.uk/resources/software/smalt/; SOAPdenovo, http://soap.genomics.org.cn; RepeatModeler, http://www.repeatmasker.org/RepeatModeler.html; RepeatMasker, http://www.repeatmasker.org; Repbase, http://www.girinst.org/repbase/; Timetree, http://www.timetree.org/;Ensembl, ftp://ftp.ensembl.org/pub/; http://evolution.genetics.washington.edu/phylip.html; PAML, http://abacus.gene.ucl.ac.uk/software/paml.html; GLEAN, https://github.com/glean/glean; Interpro, http://www.ebi.ac.uk/interpro/; Infernal, http://eddylab.org/infernal/; Rfam, http://rfam.xfam.org/; OrthoMCL, http://orthomcl.org/orthomcl/; Mrbayes, http://mrbayes.sourceforge.net/; SSAHA_Pileup, ftp://ftp.sanger.ac.uk/pub/zn1/ssaha_pileup/; HISAT2, http://ccb.jhu.edu/software/hisat2/index.shtml.

## Additional files

Additional file 1: Figs. S1–S11 and Tables S1–S17.

Additional file 2: supporting data for Fig. 1b, Fig. 2c, Fig. 2d, and Fig. 4.

Additional file 3: list of duplicated protein gene families of *D. mawsoni* and *E. maclovinus*.

Additional file 4: list of DEGs between *D. mawsoni* and 2 negatively buoyant notothenioids.

## Abbreviations

AFGP: antifreeze glycoprotein; bp: base pairs; BUSCO: benchmarking universal single-copy orthologs; CHO: Chinese hamster ovary; CTGF: connective tissue growth factor; DEG: differentially expressed genes; ECM1: extracellular matrix protein 1; GO: gene ontology; gpx4: glutathione peroxidase 4; HSPG2: prostaglandin-endoperoxide synthase 2; JDP2: Jun dimerization protein 2; KEGG: Kyoto Encyclopedia of Genes and Genomes; LINE: long interspersed transposable elements; mRNA: messenger RNA; MSC: mesenchymal stem cell; mya: million years ago; NCBI: National Center for Biotechnology Information; PBS: phosphate-buffered saline; PPAR: peroxisome proliferator-activated receptor; RNA-Seq: RNA sequencing; rRNA: ribosomal RNA; SDS-PAGE: sodium dodecyl sulfate polyacrylamide gel electrophoresis; SNP: single-nucleotide polymorphism; SO: Southern Ocean; SRFP1: secreted frizzled-related protein 1; TBST: Tris-buffered saline with Tween 20; TE: transposable element; TGF-β: transforming growth factor β; tRNA: transfer RNA; ZP: zona pellucida protein.

## Competing interests

The authors declare they have no competing financial interests.

## Funding

The work was supported by grants from the Natural Science Foundation of China (No. 41761134050, 31572611, 31572598) and the Major Science Innovation Grant (2017-01-07-00-10-E00060) from the Shanghai Education Committee, the Key Achievement Supporting Grant from Laboratory for Marine Biology and Biotechnology, Qingdao National Laboratory for Marine Science and Technology to L.C., and the USA National Science Foundation Polar Programs grant ANT1142158 to C.H.C.C.

## Author contributions

L.C. and C.H.C.C. conceived and managed the project and its components. C.H.C.C. and M.H. performed fish and tissue collections and sample preparations. K.R.M. and X.Z. contributed to DNA and RNA preparations and genome size determination. Y.L., M.Y., and W.L. performed genome annotation and RNA-Seq data analysis. Y.R. and S.P. conducted *de novo* genome assembly. K.T.B. confirmed the AFGP loci. Q.X., Y.F., and L.C. designed and performed the biological experiments. Sample preparation and genome sequencing were carried out by S.J., W.Z., and J.W. L.C., Q.X., Y.L., and C.H.C.C. analyzed the data as a whole and wrote the manuscript, and C.H.C.C. and W.W. contributed interpretation of data and edits to the manuscript.

## Supplementary Material

GIGA-D-18-00328_Original_Submission.pdf

GIGA-D-18-00328_Revision_1.pdf

Response_to_Reviewer_Comments_Original_Submission.pdf

Reviewer_1_Report_Original_Submission -- Nicholas Jeffery 10/2/2018 Reviewed

Reviewer_2_Report_Original_Submission -- Baocheng Guo 10/3/2018 Reviewed

Reviewer_3_Report_Original_Submission -- Ole K TÃ,rresen 10/22/2018 Reviewed

Supplemental Files
