# Supplementary material for: The genomic basis for colonizing the freezing Southern Ocean revealed by Antarctic toothfish and Patagonian robalo genomes
Source: Gigascience. 2019 Jan 31;8(4):giz016. doi: 10.1093/gigascience/giz016 (PMC6457430; doi:10.1093/gigascience/giz016)
Supplement: GIGA-D-18-00328_Revision_1.pdf [file giz016_giga-d-18-00328_revision_1.pdf]

GigaScience

The genomic basis for colonizing the freezing Southern Ocean revealed by Antarctic toothfish and Patagonia robalo genomes

--Manuscript Draft--

**Manuscript Number:**	GIGA-D-18-00328R1
**Full Title:**	The genomic basis for colonizing the freezing Southern Ocean revealed by Antarctic toothfish and Patagonia robalo genomes
**Article Type:**	Research
**Funding Information:**	Natural Science Foundation of China (41761134050) — Dr. liangbiao Chen<br>Natural Science Foundation of China (31572611) — Dr. liangbiao Chen<br>Natural Science Foundation of China (31572598) — Dr. Qianghua Xu<br>Major Science Innovation Grant from the Shanghai Education Committee (2017-01-07-00-10-E00060) — Dr. liangbiao Chen<br>USA NSF Polar Programs grant (ANT1142158) — Dr. Chi-Hing Christina Cheng<br>Key Achievement Supporting Grant from Laboratory for Marine Biology and Biotechnology, Qingdao National Laboratory for Marine Science and Technology — Dr. liangbiao Chen
**Abstract:**	The Southern Ocean is the coldest ocean on Earth but a hotspot of evolution. The bottom-dwelling Eocene ancestor of Antarctic notothenioid fishes survived polar marine glaciation and underwent adaptive radiation forming >120 species that fill all water column niches today. Genome-wide changes enabling physiological adaptations and rapid expansion of the Antarctic Notothenioids remain poorly understood. To advance our understanding, we sequenced and compared two notothenioid genomes - the cold-adapted and neutrally buoyant Antarctic toothfish Dissostichus mawsoni, and the basal Patagonia robalo Eleginops maclovinus representing the temperate ancestor. We detected >200 protein gene families that had expanded and thousands of genes that had evolved faster in the toothfish, with diverse cold-relevant functions including stress response, lipid metabolism, protein homeostasis and freeze resistance. Besides AFGP, an eggshell protein had functionally diversified to aid in cellular freezing resistance. Genomic and transcriptomic comparisons revealed proliferation of Selcys-tRNA genes and broad transcriptional upregulation across anti-oxidative selenoproteins, signifying their prominent role in mitigating oxidative stress in the oxygen-rich Southern Ocean. We found expansion of transposable elements, temporally correlated to Antarctic notothenioid diversification. In addition, the toothfish exhibited remarkable shifts in genetic programs towards enhanced fat cell differentiation and lipid storage, and promotion of chondrogenesis while inhibiting osteogenesis in bone development, collectively contributing to achieving neutral buoyancy and pelagicism. Our study revealed a comprehensive landscape of evolutionary changes essential for Antarctic notothenioid cold adaptation and ecological expansion. The two genomes are valuable resources for further uncovering mechanisms underlying the spectacular notothenioids radiation driven by the coldest environment.
**Corresponding Author:**	liangbiao Chen<br>Shanghai Ocean University<br>Lingang New City, CHINA
**Corresponding Author Secondary Information:**	
**Corresponding Author's Institution:**	Shanghai Ocean University
**Corresponding Author's Secondary Institution:**	
**First Author:**	liangbiao Chen

Powered by Editorial Manager® and ProduXion Manager® from Aries Systems Corporation

First Author Secondary Information:	
Order of Authors:	liangbiao Chen
	Ying Lu
	Wenhao Li
	Yandong Ren
	Mengchao Yu
	Shouwen Jiang
	Yanxia Fu
	Jian Wang
	Sihua Peng
	Kevin T. Bilyk
	Katherine R. Murphy
	Xuan Zhuang
	Mathias Hune
	Wanying Zhai
	Wen Wang
	Qianghua Xu
	Chi-Hing Christina Cheng
Order of Authors Secondary Information:	
Response to Reviewers:	Response to reviewers' comments<br><br>Reviewer #1: Review of "Genomic bases for colonizing the freezing Southern Ocean revealed by the genomes<br>of Antarctic toothfish and Patagonia robalo"<br>This manuscript sequences the genome and transcriptome of Antarctic tootfish and its closest temperate relative, the Patagonian robalo. The authors detect >200 protein gene families that have expanded with functions in stress response and freeze resistance. The amount of work conducted in this study is impressive. I applaud the authors for estimating the genome size using both the k-mer frequency distribution and flow cytometry to compare to the final assembly lengths. I am particularly interested in the expansion of transposable elements correlate to notothenioid diversification. I find no major flaws with the study, but would like to see more detail on the assembly steps (e.g. parameters tested and used in SoapDeNovo and SSPACE).<br><br>Minor comments:<br>Suggest a review for spelling and English grammar throughout the paper.<br>Thank you for your suggestion. The manuscript has been edited by two professors teach in US universities (Prof. Chi-Hing C. Cheng, University of Illinois, and Prof. George Somero, Stanford University who are native English speakers).<br><br>L69: is the >90% catch from commercial fisheries?<br>No, it is from many studies based on random sampling in the Southern Ocean. We added this information<br><br>L141: do the authors know why both the scaffold and contig N50 lengths are so much lower in robalo compared to toothfish?<br><br>For both species, collected red blood cells were embedded into agarose plugs till isolation of the genomic DNA by the same protocol. However, the Robalo DNA for some unidentified reasons is easier to degrade, which resulted in lower molecular weight of isolated genomics DNA in robalo,which is approximately 25 kb compared to 40 kb in toothfish. Accordingly, sizes of the constructed sequencing libraries ranged

from 170 to 40,000 bp in toothfish, while 170 to 20,000 bp in robalo. Thus spans of the Matepair reads used in scaffolding for the robalo assemblies are smaller than those of the toothfish so that the final scaffold N50 length of the robalo genome is lower. Althouth we considerably increased the sequencing depth of the paired-end libraries in robalo, size of the assembled contigs were still lower.
.
In Fig. 4 "kidney" is spelled incorrectly on the "caudal kidney" label
Thanks. This label in Fig.4 has been corrected in the revision.

Reviewer #2: In this study, Chen and colleagues did genome sequencing of two notothenioids to understand genetic basis of Antarctic notothenioids adaptation to the Southern Ocean. I do think that the methodology of the study is sound and findings here are solid. The genomic resources are valuable for further studying genetic basis of the notothenioids adaptive radiation as the authors state. Before recommendation for its final publishing in GigaScience, I have the following concerns that authors might consider. 1) The authors might consider to incorporate the earlier sequenced notothenioid genome. I see that the authors have included the Antarctic bullhead notothen genome in their comparison analyses but do make fair comparison among the three notothenioid genomes. For example, the authors speculated that TE might contribute to genome size increasing in derived notothenioids by comparison between Antarctic toothfish and Patagonia robalo. What about TEs in the Antarctic bullhead notothen genome? What about LINEs in Antarctic bullhead notothen genome? A through comparison among the three notothenioid genomes might give us more information about the topic the authors are trying to beat.

Thank you for suggestion. Comparison of the TEs among Antarctic toothfish, Patagonia robalo and Antarctic bullhead notothen genomes has been added to this revision. Accumulation of TEs are observed in both Antarctic toothfish and bullhead notothen genomes. We thus modified the previous statement regarding TE content and genome size as follows: “The doubling of TE content in the D. mawsoni and N. coriiceps genomes suggests higher activity of TEs in theAntarctic species in relative to the basal robalo, suggesting a likely contributing factor to the observed trend of increasing genome sizes in more derived Antarctic notothenioid lineages”. As for as the timing of LINE insertion is concerned, we calculated their insertion time in the N. coriiceps genome by the same methodology as in D. mawsoni and E. maclovinus. A similar trend of expansion is observed, in D. mawsoni and N. coriiceps. The corresponding results has been added to Fig.2a in the revision.

2) As findings in their earlier works, the authors find that gene duplication plays an important role in adaptation to the freezing Southern Ocea in notothenioids. I am wondering how many gene families experienced duplication have been identified by both this study and their 2008 PNAS study.

As we stated in the manuscript, “Due to inherent inefficiency in correctly assembling highly similar DNA sequences in the shotgun sequencing strategy, there are likely many more duplicated genes that had eluded detection”. From the set of duplicated genes we identified through comparative genome hybridization (Chen et al., 2008) previously, We found 23 protein coding genes are shown significantly duplicated in D. mawsoni genome in relative to E. maclovinus which was shown in Additional file 1: Fig. S4b. Among these genes included zona pellucida domain containing protein C5 (ZPC5), multiple banded antigen (previously a novel gene), serum lectin isoform 1 precursor (previously FBP32II) and hepcidin.
Many types of ZPs, such as ZPAX1, ZPC1, ZPC2 failed to detect as duplicated in this study, but are known to undergone substantial duplication through array-based genome hybridization and quantitative PCR (Cao et al., 2016),indicating the limitation of the shotgun genome sequencing strategy in finding gene duplications.

References
28. Chen, Z. et al. Transcriptomic and genomic evolution under constant cold in Antarctic notothenioid fish. Proc. Natl. Acad. Sci. USA 105, 12944-12949 (2008).
29 Xu, Q. et al. Adaptive evolution of hepcidin genes in antarctic notothenioid fishes.

Powered by Editorial Manager® and ProduXion Manager® from Aries Systems Corporation

Mol. Biol. Evol. 25, 1099-1112 (2008).
30 Cao, L. et al. Neofunctionalization of zona pellucida proteins enhances freeze-prevention in the eggs of Antarctic notothenioids. Nat. Commun. 7, 12987 (2016).

3) The authors used an RNA-seq method for their study of transcriptomic adaptation to the freezing environment. However, I do not see any details how they collected the tissues, as we all know that such analysis is very sensitive to the sampling strategy.

Thank you for your suggestion. We added the following information to the Materials and Methods section:

“To obtain tissues from the large-sized D. mawsoni, live specimen was anesthetized with MS222 (tricaine methanesulfonate) inside a ambient seawater filled floating sheet plastic tubing in the aquarium tank. The anesthetized specimen was then put on a V-shaped trough for dissection. Tissues were quickly removed and cut into small pieces on ice, and immediately immersed and shaken in ≥10 volumes of pre-chilled (-20℃) 90% ethanol (made with 100% pure ethanol and sterilized MilliQ Type 1 water). The ethanol was replaced with a fresh volume within 10 minutes, and again at 2-3 hours and 12 hours later. This preservation method serially desiccates the tissue and effectively inactivates tissue nucleases. The tissue samples were kept in -20℃ freezer throughout the serial preservation process and then stored at -20℃ until use. To obtain tissues from E. maclovinus, MS222 anesthetized specimen was quickly dissected on ice, and preserved in -20℃ as described for D. mawsoni. The ethanol preserved tissues were shipped back to the University of Illinois on dry ice.”

Reviewer #3: Reviewer report.

Title: Genomic bases for colonizing the freezing Southern Ocean revealed by the genomes of Antarctic toothfish and Patagonia robalo

General comments
The authors have sequenced and assembled the genomes of two notothenioids, and have done extensive comparisons with regards to expansions of gene families and differential expression of genes. They show that several genes in the D. mawsoni has undergone positive selection, highlighting the evolution of the genes of that species.

Specific comments

Abstract: An extant species is not necessary a proxy for an extinct species.
Thank you for your suggestion. The sentence that mentioned the proxy is in Introduction. We corrected it according to the reviewer's comment in revision.

Introduction:
Line 89-90: You specify "whole genome sequence analysis" as the criteria for mentioning the Antarctic rockcod as the only notothenioid reported so far, but Malmstrøm et al 2016 (https://www.nature.com/articles/ng.3645) did publish genomic sequences and the assembly of Chaenocephalus aceratus. However, they did not report any genomic/biological features of that particular species, so your phrasing is entirely correct.

Thank you for your comments. We have added this citation in the section of Introduction. The corresponding sentence was corrected as: “Thus far, whole genome sequence analysis has been reported for only one notothenioid species, the Antarctic rockcod Notothenia coriiceps (Shin et al., 2014). A major histocompatibility complex gene loci from Chaenocephalus aceratus was also reported (Malmstrøm M, et al (2016)."

Line 107-8: As you no doubt are aware of, size do not necessary have any bearing on

	buoyancy, only average density. It is not apparent to me that smaller size would mean easier to achieve neutral buoyancy.<br><br>Throughout the manuscript, we agree that neutral buoyancy is related to the average density of fish, not smaller size. Enhanced lipid storage and promotion of chondrogenesis while inhibiting osteogenesis in bone development play important roles for the D. mawsoni to achieve the neutral buoyancy.<br><br>We guess the misunderstanding of the reviewer might have resulted from our description on the evolution of smaller ZPC5 molecules in D. mawsoni, which is related to the enhanced capability of intracellular freezing-resistance in D. mawsoni, NOT related to neutral buoyancy, and nothing to do with body size of the fish.<br><br>Results:<br>Line 138: Why was two different genome assemblers used? Also, in the header for Table S2b it is stated that E. maclovinus was assembled with both SOAPdenovo and Platanus.<br><br>As we stated in answering reviewer #1's question, E. maclovinus DNA extracted from similarly prepared agarose plugs exhibited lower molecular weight than D. mawsoni for unknown reasons, which resulted in lower quality of the E. maclovinus assemblies. To increase the E. maclovinus contig length and decrease algorithm bias when a single assembler was used, we parallelly built the contigs by two assemblers SOAPdenovo and Platanus. The generated contigs were merged prior to the scaffold building.<br><br>Line 140 and other places across the manuscript: "Kb", that is, kilo base pairs, should be abbreviated "kb(p)". See: https://en.wikipedia.org/wiki/Metric_prefix<br><br>Thank you for your comment. We check the abbreviation of "Kb" and "Kbp" in several journals. "Kb", as the abbreviation of kilo base pairs, is used in most of the journals. But for the abbreviation of less than 1000 base pairs, "bp" is used. So this manuscript betters to follow the universal usage, as "Kb".<br><br><br>Line 164: The number of common genes is a bit strange. The vast majority of genes should be common between these species. I think you have written this wrong. In the referred figure, S3, it is specified that the number 8,825 is the amount of common gene clusters, and not just genes. One cluster might contain multiple genes.<br><br>Thanks. That should be 8,825 gene clusters, not genes. We have corrected it in the revision.<br><br>Lines 182-192: You stated earlier "842 Mb for D. mawsoni and 727 Mb for E. maclovinus". You could say that quite a bit of that difference in genome size could be due to differences in repeat content, and not just percentage. 161.8 Mbp TEs in D. mawsoni and 74.6 Mbp in E. maclovinus, with a difference of 86.2 Mbp. It is not apparent that the percentages differences in repeat content actually translates to those large differences in repeats, because these repeat annotations can be quite different (many repeats are not annotated properly in different genomes).<br><br>Thank you for your suggestion. Annotation of the TEs in Antarctic toothfish, Patagonia robalo and Antarctic bullhead notothen (added in the revision) are conducted with the same pipelines and criteria. We compared TE contents (%) among the three genomes. Accumulation of TEs are observed in both Antarctic toothfish and bullhead notothen genomes, which may partially contributed the enlargement of genome size in the Antarctic notothenioids. We agree that repeat in genomes may not correctly annotated. We also correct two numbers, the TEs contents of D. mawsoni (21.38%) and E. maclovinus(10.02%), in this section, where the errors occurred in the previous version due to a mistake when citing from the results from the Additional file 1: Table S9.<br><br><br>Line 613: It is InterProScan, and not InterproScan.<br>Thanks. We have corrected it in the revision.
**Additional Information:**	

Powered by Editorial Manager® and ProduXion Manager® from Aries Systems Corporation

Question	Response
Are you submitting this manuscript to a special series or article collection?	No
**Experimental design and statistics**<br><br>Full details of the experimental design and statistical methods used should be given in the Methods section, as detailed in our Minimum Standards Reporting Checklist. Information essential to interpreting the data presented should be made available in the figure legends.<br><br>Have you included all the information requested in your manuscript?	Yes
**Resources**<br><br>A description of all resources used, including antibodies, cell lines, animals and software tools, with enough information to allow them to be uniquely identified, should be included in the Methods section. Authors are strongly encouraged to cite Research Resource Identifiers (RRIDs) for antibodies, model organisms and tools, where possible.<br><br>Have you included the information requested as detailed in our Minimum Standards Reporting Checklist?	Yes
**Availability of data and materials**<br><br>All datasets and code on which the conclusions of the paper rely must be either included in your submission or deposited in publicly available repositories (where available and ethically appropriate), referencing such data using a unique identifier in the references and in the "Availability of Data and Materials" section of your manuscript.	Yes

Have you have met the above requirement as detailed in our Minimum Standards Reporting Checklist?	

Powered by Editorial Manager® and ProduXion Manager® from Aries Systems Corporation

Manuscript
Click here to view linked References
Click here to access/download;Manuscript;Dmawsoni-Emaclovinus-maintext-revised26Nov2018-SCE_Final(1).docx

The genomic basis for colonizing the freezing Southern Ocean revealed by Antarctic toothfish and Patagonia robalo genomes

Liangbiao Chen[1,2,3,*], Ying Lu[1,2,*], Wenhao Li[1,2,*], Yandong Ren[4], Mengchao Yu[1,2], Shouwen Jiang[1,2], Yanxia Fu[1,2], Jian Wang[1,2], Sihua Peng[1,2], Kevin T. Bilyk[5], Katherine R. Murphy[5], Xuan Zhuang[5], Mathias Hune[6], Wanying Zhai[1,2], Wen Wang[4], Qianghua Xu[1,2,§], Chi-Hing Christina Cheng[5,6,§]

[1]National Demonstration Center for Experimental Fisheries Science education at Shanghai Ocean University, Shanghai, China, [2]Key Laboratory of Exploration and Utilization of Aquatic Genetic Resources (Ministry of Education) and International Research Center for Marine Biosciences (Ministry of Science and Technology) at Shanghai Ocean University, Shanghai, China, [3]Laboratory for Marine Biology and Biotechnology, Qingdao National Laboratory for Marine Science and Technology, Qingdao, China, [4]Kunming Institute of Zoology, Chinese Academy of Sciences, Kuming, China, [5]Department of Animal Biology, University of Illinois at Urbana-Champaign, IL, USA, [6]Fundación Ictiológica, Providencia, Santiago, Chile.

[*] These authors contributed equally to this work.

[§]Corresponding authors：

Qianghua Xu (qhxu@shou.edu.cn).

Chi-Hing Christina Cheng (c-cheng@illinois.edu).

ORCID IDs:

Qianghua Xu, ORCID: 0000-0003-0351-1765

Chi-Hing Christina Cheng, ORCID: 0000-0001-6308-8685

Katherine R. Murphy, ORCID: 0000-0001-8898-8719

Kevin T. Bilyk, ORCID: 0000-0002-2262-2703

Abstract

The Southern Ocean is the coldest ocean on Earth but a hotspot of evolution. The bottom-dwelling Eocene ancestor of Antarctic notothenioid fishes survived polar marine glaciation and underwent adaptive radiation forming >120 species that fill all water column niches today. Genome-wide changes enabling physiological adaptations and rapid expansion of the Antarctic Notothenioids remain poorly understood. To advance our understanding, we sequenced and compared two notothenioid genomes - the cold-adapted and neutrally buoyant Antarctic toothfish *Dissostichus mawsoni*, and the basal Patagonian robalo (also known as the Patagonian blenny) *Eleginops maclovinus* representing the temperate ancestor. We detected >200 protein gene families that had expanded and thousands of genes that had evolved faster in the toothfish, with diverse cold-relevant functions including stress response, lipid metabolism, protein homeostasis and freeze resistance. Besides AFGP, an eggshell protein had functionally diversified to aid in cellular freezing resistance. Genomic and transcriptomic comparisons revealed proliferation of Selcys-tRNA genes and broad transcriptional upregulation across anti-oxidative selenoproteins, signifying their prominent role in mitigating oxidative stress in the oxygen-rich Southern Ocean. We found expansion of transposable elements, temporally correlated to Antarctic notothenioid diversification. Additionally, the toothfish exhibited remarkable shifts in genetic programs towards enhanced fat cell differentiation and lipid storage, and promotion of chondrogenesis while inhibiting osteogenesis in bone development, collectively contributing to achieving neutral buoyancy and pelagicism. Our study revealed a comprehensive landscape of evolutionary changes essential for Antarctic notothenioid cold adaptation and ecological expansion. The two genomes are valuable resources for further exploration of mechanisms underlying the spectacular notothenioid radiation in the coldest marine environment.

Keywords: adaptive radiation, climate change, genome, oxidative stress, Antarctic notothenioids

Introduction

The Southern Ocean (SO) surrounding Antarctica is the coldest body of water on Earth, having been isolated from other world oceans by the Antarctic Circumpolar Current (ACC) beginning in the early Oligocene ~32 million years ago (1). The formation of the ACC also impeded species dispersal across the Antarctic Polar Front, and mass extinction of the

Antarctic-sequestered fish taxa occurred upon marine glaciation (2). The rich cosmopolitan fish fauna prior to the isolation of Antarctica is represented today by a single predominant group of related fish species - the Antarctic notothenioids. From a common temperate ancestor, likely a swim-bladderless, bottom dwelling perciform species of the Eocene age (3), the Antarctic notothenioids have evolved to become highly adapted to life in unyielding cold, spectacularly diverse in sizes and morphological innovations, and diversified into all water column habitats, epitomizing an adaptive radiation and a rare marine species flock (4). Abundant in fish biomass (>90% of random catch) and species (≥128), they are vital in sustaining the contemporary SO food web (2, 5).

What evolutionary processes and mechanisms propelled the Antarctic notothenioid radiation replete with extraordinary trait diversification during its evolutionary history remain fascinating unanswered questions. Two conspicuous trait outcomes - the evolutionary gain of the novel antifreeze glycoprotein (AFGP) gene and function that averted otherwise inescapable death from freezing (6, 7), and exploitation of open niches vacated by extinction of fishes lacking freeze-resistance, have been recognized as major contributors to the Antarctic notothenioid radiation (8, 9). However, little is known of the myriad subtler adaptive changes that must also have evolved in response to challenges from freezing temperatures and the associated high oxygen concentration – the two foremost modalities of selection pressure from the SO environments that would pervade all levels of organismal functions, from molecules to cells to system physiology. Another prominent hallmark of notothenioid adaptive radiation is the secondary acquisition of pelagicism in some lineages, enabling their ecological expansion from bottom habitats of their negatively buoyant ancestor to upper water column niches. What evolutionary changes occurred in the cellular and developmental programs that enabled neutral buoyancy and secondary pelagicism are also unknown.

To address these fundamental, system-wide questions about Antarctic notothenioid evolution, whole genome sequences of multiple and appropriately chosen species from the diverse Antarctic notothenioids are essential. Thus far, whole genome sequence analysis has been reported for only one notothenioid species, the Antarctic rock cod *Notothenia coriiceps* (10). A major histocompatibility complex gene loci from *Chaenocephalus aceratus* was also reported (11). The *N. coriiceps* genome provided the key inference that the fast evolving hemoglobin and mitochondrial proteins are adaptive in increasing efficiency of aerobic cellular respiration in the freezing environment. *N. coriiceps* is not known to occur in the high latitude Antarctic coastal waters. Instead, it is widely distributed in the lower latitude waters

of the Antarctic Peninsula archipelago and the Scotia Arc islands, reaching localities north of the Polar Front around sub-Antarctic islands in the Indian Ocean sector (12), a distribution pattern that suggests a considerable degree of thermal plasticity in this species. It is a heavy, bottom fish and one of the hardest boned Antarctic notothenioids (13), reminiscent of the benthic ancestor. To gain insights into evolutionary adaptations in the most cold-adapted and stenothermal Antarctic notothenioids, as well as into the evolutionary changes leading to acquisition of neutral buoyancy that enabled the transition from the ancestral benthic existence to a pelagic life history, a different and more appropriate model Antarctic notothenioid species would be required.

The Antarctic toothfish *Dissostichus mawsoni* (NCBI: txid6530, Fishbase ID:7039) that grows to giant sizes (2.0 m in length and 140 kg in mass) is an iconic species of the Antarctic notothenioid radiation, with wide distributions in freezing waters of high latitude Antarctic coasts, as far south as 77.5^{o}S (McMurdo Sound), the southern limit of Antarctic marine life. It thus exemplifies the stenothermal cold-adapted character state. Despite its large size, it is the only notothenioid species that achieved complete neutral buoyancy as adults (14, 15); thus this species serves as the best model for examining the evolutionary underpinning of secondary pelagicism in the Antarctic clade. In addition, to discern evolutionary changes from the ancestral temperate state to the derived polar state driven by selection in the cold, oxygen-rich Southern Ocean environment, a closely related basal non-Antarctic notothenioid comparison species would strongly add to the discriminating power of analyses of genome evolution. The most appropriate species for this purpose is a South American notothenioid, the Patagonian robalo *Eleginops maclovinus* (NCBI: txid56733, Fishbase ID:466), which is the sole species in the basal family Eleginopsidae. The lineage diverged prior to the isolation of Antarctica, and *E. maclovinus* is phylogenetically the closest sister species to the modern Antarctic clade (3). Thus its genome is the best representative of the temperate character of the most recent common ancestor of the Antarctic notothenioids. We conducted genome sequencing and comparative analyses of these two strategically selected species accompanied with extensive transcriptomic characterizations to profile relevant functional outcomes of the genomic changes. Our results provide several new key insights into evolutionary adaptation and secondary pelagicism of the Antarctic notothenioids in the isolated and extremely cold Southern Ocean environment.

Results and Discussion

D. mawsoni and E. maclovinus genome sequencing and assembly

The geographic distributions and sampling locations of *D. mawsoni* and *E. maclovinus* are illustrated in **Fig. 1a**. The genome of one *D. mawsoni* juvenile (12 kg) of undetermined sex, and one young adult male *E. maclovinus* (~100 gm) were *de novo* sequenced using Illumina sequencing platforms. The genomes of both species comprise 24 pairs of chromosomes ($2n = 48$) (16, 17). Analyses of 17-mer frequency distribution indicated a genome size of approximately 842 Mb for *D. mawsoni* and 727 Mb for *E. maclovinus* (**Additional file 1: Fig. S1**), consistent with the mean genome sizes of 840 Mb and 780 Mb for the toothfish and robalo respectively determined by flow cytometry (**Additional file 1: Table S1a**). The raw sequence data after cleaning and error correction (**Additional file 1: Tables S1b, S1c**) were assembled using SOAPdenovo (18) for *D. mawsoni*, and Platanus (19) for *E. maclovinus* followed by scaffold building with SSPACE (20). The assembled toothfish genome had a contig N50 length of 23.1 Kb and scaffold N50 length of 2.2 Mb, while those of the robalo were 10.9 Kb and 0.69 Mb. The assembled toothfish and robalo genomes are approximately 757 Mb and 744 Mb respectively (**Table 1; Additional file 1: Tables S2a, S2b**), consistent with *k*-mer and flow cytometry estimates, and achieving over 90% and 95% coverage of the genome size based on flow cytometry of the two species respectively. The completeness of both genomes were assessed with BUSCO (Benchmarking Universal Single-Copy Orthologs) (21), referencing the lineage dataset of actinopterygii_odb9 and orthologs of zebrafish, which reflected the complete BUSCOs at 97.2% for the *D. mawsoni* genome and 95.0% for the *E. maclovinus* genome (**Additional file 1: Tables S2c**). The GC content of the *D. mawsoni* genome is 0.4070, nearly identical to 0.4066 of *E. maclovinus*, and both are lower than that of a model fish the stickleback *Gasterosteus aculeatus* (**Additional file 1: Fig. S2**). The accuracy of the genome assembly was assessed by alignment of the scaffolds to publically available unigenes of *D. mawsoni* and *E. maclovinus*, and the coverage of the initial contigs was found to be approximately 98.8% and 99.1%, respectively (**Additional file 1: Table S3**), suggesting an acceptable quality of the genome assemblies. Alignment of the sequence reads to the assemblies estimated an overall heterozygous rate of approximately 2.58 and 2.40 per Kb for *D. mawsoni* and *E. maclovinus* respectively. (**Additional file 1: Table S4**).

Genome annotation and synteny alignment between D. mawsoni and E. maclovinus

A total of 22,516 and 22,959 protein-coding genes were annotated in the *D. mawsoni* and *E. maclovinus* genome, respectively by combining the results from homologous and *de novo* prediction methods using the gene modeler GLEAN (**Additional file 1: Table S5**). The

protein coding genes of the toothfish and robalo, along with the sequenced notothenioid *N. coriiceps* and model species *G. aculeatus* and zebrafish *Danio rerio* were clustered using OrthoMCL(22). We found 8,825 gene clusters that were common to all five species. Genes shared among the notothenioids are similar in number, 12,269 between toothfish and robalo, and 12,421 between toothfish and *N. coriiceps* (**Additional file 1: Fig. S3**). In annotations of conserved non-coding RNA genes, we predicted 1,097 tRNA, 110 rRNA, 422 SnRNA, and 295 microRNA genes in the toothfish genome (**Additional file 1: Table S6a**), while the robalo genome was annotated to carry 1,037 tRNA, 44 rRNA, 891 snRNA and 286 miRNAs (**Additional file 1: Table S6b).** The much larger number of rRNA copies (2.5 fold) in *D. mawsoni* than *E. maclovinus* is consistent with the presence of dual chromosomal loci of rDNA genes detected by *in situ* fluorescent hybridization in the giant toothfish (16), as opposed to the single rDNA locus in other notothenioids (23). The two species showed largely similar profiles in their microRNAome, with minor differences in the copy number of some individual microRNA (**Additional file 1: Table S7**). Strikingly, the toothfish genome contains many more selcys-tRNA genes than robalo (84 versus 1; **Additional file 1: Table S8**). This extensive duplication of selcys-tRNA genes, accompanied with high expression of selenoproteins in *D. mawsoni* (detailed in a later section) signify that mitigation of oxidative stress through selenoproteins, many of which are strong antioxidants, is likely an important selection force in the evolution of the Antarctic notothenioid genome in the freezing and oxygen-rich waters.

De novo annotation of repeat sequences revealed >2-fold increase in overall repeat content in *D. mawsoni* (21.38% of genome) over the basal *E. maclovinus* (10.02%) (**Additional file 1: Table S9**). Transposable elements (TEs) of the toothfish genome, including LTR-retrotransposons, non-LTR-retrotransposons (long interspersed transposable elements - LINEs, and short interspersed transposable elements - SINEs), and DNA transposons , was more than twice that of *E. maclovinus*. Examination of the *N. coriiceps* genome exhibited similar accumulation of the TEs (23.5%). Among simple sequence repeats (SSRs), dimer repeats constituted the majority of the SSRs in both genomes, and tetramers and pentamers showed highest levels of increment in *D. mawsoni* (**Additional file 1: Table S10a, Table S10b**). The doubling of TE content in the *D. mawsoni* and *N. coriiceps* genomes suggests higher activity of TEs in theAntarctic species in relative to the basal robalo, suggesting a likely contributing factor to the observed trend of increasing genome sizes in more derived Antarctic notothenioid lineages (24).

For global genome alignment, we anchored the *D. mawsoni* and *E. maclovinus* scaffolds to the 21 linkage groups of the well characterized three-spined stickleback genome according to gene collinearity (**Fig. 1b; Additional file 2**). Most of the notothenioid scaffolds had extensive collinearity to the corresponding stickleback chromosomes. The 84 duplicated selcys-tRNA genes are wide-spread throughout the toothfish genome. Inversion and translocation of genome segments have occurred in both *D. mawsoni* and *E. maclovinus* relative to stickleback, but the *D. mawsoni* genome showed more frequent rearrangements. Insertion sites for the expanded TEs of the *D. mawsoni* genome were random, thereby expanding the lengths of almost all of the linkage groups. Mapping RNAseq transcript data we obtained from white muscles for the two species on their respective synteny showed that the heavier insertion of TEs in the toothfish genome did not appear to adversely affect the expression of the neighboring protein genes as strong gene expression in regions of high TE content was maintained (**Fig. 1b**).

Burst of LINE expansion in the cold

Involvement of gain (or loss) of mobile element copies in genome size and genome re-structuring affecting species differentiation has increasingly gained empirical support (25, 26). TEs are normally under epigenetic regulation, but waves of TE proliferations could arise from environmental changes that cause physiological stress and disrupt the epigenetic control (27). We therefore examined potential linkage in timing between LINE expansion (2-fold increase in the toothfish versus robalo) and onset of frigid SO marine conditions. A multiple sequence alignment of several LINEs (LINE/I, LINE/L2, LINE/RTE-BovB and LINE_Rex-Babar – the most abundant types in the toothfish genome) was made, which clustered 349 *D. mawsoni* LINE pairs and 213 *E. maclovinus* LINE pairs, where the two LINEs within each pair share the highest sequence similarity, approximating the least divergence time. Calculation of the nucleotide substitution rates for each LINE pair identified a burst of emergence of LINEs in the *D. mawsoni* genome with the substitution rates centered at 0.04, which corresponded with a divergence time of 6.5 million years ago (**Fig. 2a; see also Methods**) based on the average substitution rate of the LINEs. A similar trend of LINE expansion was also observed in the *N. coriiceps* genome. This estimated timing of LINE burst expansion correlated with the radiation of the majority of the modern Antarctic notothenioid clades beginning in the late Miocene when seawater temperatures steadily declined (9). In contrast, no burst expansion of LINEs was detected in the *E. maclovinus* genome, supporting an Antarctic/cold-specific LINE burst in the toothfish, and corroborating our prior empirical

evidence for an increase in retrotransposition activity of *D. mawsoni* LINE-1 resulting in more copies in the transfected cells when subjected to stress from non-physiologically low incubation temperature (28).

Accelerated protein evolution in the cold

Ectotherms are vulnerable to disruptive effects on protein structures and reaction rate depression at low temperatures. Antarctic notothenioids in perennially freezing high latitude waters face the extremes of these effects, as well as an oxidative environment due to high oxygen concentrations resulting from increased gas solubility at low temperatures. We examined evidence for adaptive evolution of proteins in response to these selection pressures. The gene models of *D. mawsoni*, *E. maclovinus*, seven other teleost genomes and the mouse genome as outgroup were clustered, from which 2,936 one-to-one single-copy orthologs were obtained to reconstruct a phylogenetic tree (**Fig. 2b**). Stickleback is the closest species to the notothenioid clade as expected, and *E. maclovinus* is sister to the two Antarctic notothenioids, *D. mawsoni* and *N. coriiceps*. We found evolutionary rates of the orthologous genes based on calculated *dN*/*dS* values (the ratio of the rate of non-synonymous substitution to the rate of synonymous substitution) were elevated in the notothenioid lineage compared to the other fish lineages. This faster rate is more pronounced in the two Antarctic species, about twice that of the basal *E. maclovinus*, suggesting intensified selection pressures driving genome evolution in the Antarctic environment. To identify Gene Ontology categories that were evolving faster in the toothfish or robalo, the *dN*/*dS* ratios of 7,584 orthologous genes among the two notothenioids and the stickleback were calculated and the average *dN*/*dS* value of the genes associated with each GO term was calculated for each species. These orthologous genes were annotated to 411 GO terms, of which 281 showed significantly higher average *dN*/*dS* ratios in *D. mawsoni*, while only 19 demonstrated higher average rates in *E. maclovinus* (**Fig. 2c; Additional file 2**). The faster evolving GO processes in *D. mawsoni* included “gene expression”, “protein folding”, “tRNA metabolic process”, “cell-redox homeostasis”, “immune response”, ”response to stress”, “lipid metabolic process”, “DNA repair”, “vesicle-mediated transport” and others. To assess which *D. mawsoni* genes experienced positive selection, we tested using the Branch-site model (in PAML) on a reconstructed phylogenetic tree of six fish species with the *D. mawsoni* lineage assigned as the foreground branch (**Additional file 1: Fig. S4a**). A total of 526 positively selected genes (PSGs) were identified (**Additional file 1: Table S11a**). The most significantly enriched KEGG pathway (**Additional file 1: Table S11b**) was “Protein digestion and absorption” ($p = 5.1\times10^{-5}$) and

GO term (**Additional file 1: Table S11c**) was "protein binding" ($p = 6.0\times10^{-4}$), which indicate that maintenance of protein homeostasis played an important role in shaping the *D. mawsoni* genome. In addition, a complement of genes (*sorbs2a*, *acox1*, *apoa1a*, *scp2a*, *tnni3k* and perilipin-like genes; **Additional file 1: Table S11a**) involved in the PPAR (peroxisome proliferator-activated receptors) signaling pathway - the key pathway in adipocyte development regulation - were found to be under positive selection, suggesting occurrence of adaptive changes in lipid metabolism in *D. mawsoni*.

Gene duplication in the freezing environment

Gene duplication plays fundamental roles in emergence of adaptive features. In the list of predicted protein coding genes from the toothfish and robalo genomes, we identified 202 families that have increased in copy number in *D. mawsoni*, compared to the other eight fish genomes (**Additional file 3)**. KEGG enrichment analyses of these expanded gene families yielded enrichment in pathways involved in protein homeostasis and lipid and bone metabolism, such as "Protein digestion and absorption", "Regulation of lipolysis in adipocyte", "Fat digestion and absorption", "Ether lipid metabolism" and "Osteoclast differentiation" (**Fig. 2d; Additional file 2)**, suggesting genomic capacity for these functional pathways had increased in *D. mawsoni* during evolution in chronic cold. Corroborating this cold-specificity is that the expanded gene families found in the basal *E. maclovinus* relative to the other fish genomes, including *D. mawsoni* and *N. coriiceps*, yielded distinctly different enriched KEGG profiles (**Fig. 2e**); this analysis indicates that the functional traits gained through gene duplication in *E. maclovinus* were driven by different selective pressures, consistent with our previous findings (29, 30). Due to inherent inefficiency in correctly assembling highly similar DNA sequences in the shotgun sequencing strategy, there are likely many more duplicated genes that had eluded detection. For example, many paralogs of Zona Pellucida protein (ZPs), such as ZPAX1, ZPC1, ZPC2 in *D. mawsoni* have been shown, through array-based genome hybridization and quantitative PCR, to be duplicated (29, 31). Among the set of genes found duplicated in our previous report (28), 23 are identified in this study (Additional file 1: Fig. S4b).

Gene duplication had contributed prominently to the evolutionary gain of freezing avoidance in Antarctic notothenioids. Generally regarded as a key innovation of the Antarctic notothenioid radiation, the AFGP (antifreeze glycoprotein) gene evolved from a trypsinogen-like protease ancestor followed by extensive intragenic and whole gene duplications, generating a large gene family that would provide an abundance of this novel

life-saving protein (7). By referencing to the published AFGP haplotypes that were assembled from BAC clone sequences of the same individual used in this study (Genbank accessions HQ447059 and HQ447060) (32), we identified and assembled the AFGP loci shotgun reads, recaptured the two AFGP haplotypes and integrated them in the draft genome, and localized them to a region syntenic with a scaffold in LG 20 of the stickleback genome (**Fig.1b**).

Resistance to freezing extends beyond the AFGPs. We found gene duplication for a protein that *a priori* would not be expected to function in freeze-resistance, zona pellucida protein (or eggshell protein), additionally provided protection against cellular freezing. Products of *ZPC5* from *D. mawsoni* have been shown to enhance freezing resistance of eggs of recipient zebrafish both *in vivo* and *in vitro* (31). In this study, we found four copies of *ZPC5* in the *D. mawsoni* genome that encoded three different sized ZPC5 proteins in the toothfish ovary - DmZPC5_1, DmZPC5_2a/DmZPC5_2b, and DmZPC_3 in decreasing order of size, corresponding to gradually shortened C-termini from the conserved ZP domain due to nonsense mutations in exon 9 and exon 10 (**Fig. 3a; Additional file 1: Fig. S5a).** A single ZCP5 gene in the basal *E. maclovinus* was found, which corresponded to the full-length DmZPC5_1. We expressed the three DmZPC5 isoforms in Chinese Hamster Ovary (CHO) cells (**Additional file 1: Fig. S5b, Fig. 3b**) and assayed cell survival rate at freezing temperature (-2ºC for 8 hrs). DmZPC5_3 was the most active isoform in maintaining cell viability while DmZPC5_1 was the least active one (**Fig. 3c**). Further analyses showed that DmZPC5_3 was more likely retained inside the cell than DmZPC5_2 andDmZPC5_1 (**Additional file 1: Fig. S6**), and less likely to become polymerized inside the cell compared with DmZPC5_1 (**Additional file 1: Fig. S7a**), corroborating our previous finding that only unpolymerized ZP proteins are active for the ice-melting promoting activity (31). We detected DmZPC5 expression across many tissues besides ovary in *D. mawsoni* (**Additional file 1: Fig. S7b**), consistent with a distribution expected of a general protective function.

Transcriptomic adaptation to the cold environment

To assess the functional relevance of the detected genomic outcomes to life in freezing condition, we characterized and compared transcriptomes of 12 tissues including brain, liver, red muscle, white muscle, gill, skin, intestine, stomach, spleen, head kidney, caudal kidney and ovary between native specimens of *D. mawsoni* and *E. maclovinus*. We found over ten thousand genes were differentially expressed in pairwise comparisons between the two species, with the toothfish showing substantially more up-regulated genes than the robalo. Enrichment test on KEGG pathways yielded many signaling pathways in the tissues of

toothfish being significantly enriched in differentially expressed genes (DEGs), including the TGF-beta, AMPK and PPAR pathways, known to play essential roles in development, metabolism and stress responses (**Additional file 1: Fig. S8a**). GO enrichment analysis of the DEGs demonstrated up-regulation of hundreds of GO biological processes, including translation, transferrin transport, cell redox homeostasis, cellular response to unfolded protein, ubiquitin-dependent protein catabolic process, regulation of innate immunity, MAPK cascade, positive regulation of apoptosis pathways, many of the pathways involved in lipid metabolism, and anti-ROS pathways represented by "selenium compound metabolic process" and "selenocysteine metabolic process" (**Additional file 1: Fig. S8b**). These results corroborated the expression profiles in a previous study with the lower depth of sequencing available at that time (29), and additionally revealed further details of transcriptomic cold adaptation from the much deeper sequencing across a comprehensive set of tissue transcriptomes. A striking finding was the greatly increased transcriptional activities across many selenium-containing protein genes in the toothfish tissues compared with robalo (**Fig. 4; Additional file 1: Fig. S9**). Correspondingly, the genes involved in the translation of selenocysteine-containing proteins were also significantly up-regulated in the toothfish (**Fig. 4**). Selenoproteins have well-known functions in coping with cellular oxidative stress, thus the great expansion of selcys-tRNA genes (**Fig. 1b**, **Additional file 1: Table S8**), and the significantly upregulated expression of many kinds of selenoprotein mRNAs indicate augmented anti-ROS capacity has evolved as an important adaptation to the constantly freezing environment, where saturated levels of O_2 and cold-depressed metabolic rates would make oxidative stress a formidable challenge for cellular life. Interestingly, the expression of glutathione peroxidase 4b (gpx4b) (**Fig. 4**), a selenoprotein uniquely able to reduce lipid hydroperoxides (33, 34), was lower in *D. mawsoni*, suggesting alternative lipid metabolic programs may exist in the toothfish. Accordingly, we found all isoforms of the major players in lipid droplet assembly (PLN2, PLN5, fitm, seipin) important for lipid storage in adipose tissue, were upregulated in all toothfish tissues examined relative to *E. maclovinus*, signifying a shift of lipid distribution towards storage in *D. mawsoni*, as described in detail below.

Altered lipid metabolism in *D. mawsoni* for neutral buoyancy

In a striking evolutionary departure from the heavy, bottom-water ancestral character (exemplified by *E. maclovinus*), a handful of Antarctic notothenioids have secondarily acquired neutral or near neutral buoyancy, enabling ecological diversification into and filling of mid-water niches – a distinctive hall mark of the Antarctic notothenioid adaptive radiation.

The giant toothfish *D. mawsoni*, despite growing to massive sizes, being robustly muscled, and lacking a swim bladder, is the only notothenioid that has attained complete neutrally buoyancy (14, 15). Known morphological specializations include extensive lipid (mostly triglycerides) deposits under skin and in the musculature, and a light skeleton of mostly cartilage and little mineralized bone, adaptations that reduce overall density and provide static lift (14). To understand the genetic basis of the large accumulation of lipids and reduced mineralization in *D. mawsoni*, we carried out transcriptome comparisons between *D. mawsoni* and several other notothenioids in which neutral buoyancy is not developed.

We compared gene expression profiles of muscles of *D. mawsoni*, and of the negatively buoyant Antarctic *N. coriiceps* and the basal *E. maclovinus* to elucidate evolutionary differences in the mechanisms of intermuscular lipid deposit. Compared with *E. maclovinus*, genes involved in triacylglycerol synthesis in the toothfish muscle were overrepresented in DEGs ($p < 0.05$) and markedly upregulated in transcription, including the key enzymes acylglycerol-3-phosphate O-acyltransferase (AGPAT) isoforms and CDP-diacylglycerol synthase (CDS) (**Additional file 1: Fig. S10**). An important regulator of this process, lipin1, was downregulated in *D. mawsoni*. Lipin1 is known to exert dual effects on lipid metabolism - it acts as a phosphatidate phosphatase enzyme to form diacylglycerol required for lipid synthesis, but also serves as a transcriptional co-activator to promote fatty acid oxidation (35). The down-regulation of lipin1 was consistent with down-regulation of fatty acid oxidation in *D. mawsoni*, as about half of the genes involved in fatty acid oxidation were also down-regulated relative to the robalo (**Additional file 1: Table S12**). At the same time, genes involved in regulation of lipid storage were overrepresented in the DEGs ($p < 0.05$) and all but one gene (MEST) were upregulated in the toothfish (**Additional file 1: Table S13**). These data strongly suggest a shift of metabolic pathways from lipid breakdown to lipid biosynthesis and lipid storage in *D. mawsoni* muscle relative to *E. maclovinus*, favoring deposit of lipids, thus contributing to neutral buoyancy. Compared with *N. coriiceps*, *D. mawsoni* muscle showed an overall trend of upregulation of genes involved in glycerolipid biosynthesis and lipid storage, but the differences are not as dramatic as in the *D. mawsoni/E. maclovinus* comparison. Expression levels of many genes relevant to lipid oxidation were fairly similar between the two species, suggesting common downregulation in lipid oxidation in the Antarctic species compared to the temperate *E. maclovinus* (**Additional file 1: Fig. S10; Additional file 1: Tables S12, S13**). Consistent with downregulation of the lipid oxidation in the muscles of the two Antarctic fishes were their lower expression levels of lipid oxidation mitigating selenoenzyme gpx4 compared to the robalo. In total, the transcriptome

comparisons revealed substantial genetic reprogramming in *D. mawsoni* muscle that would favor the large lipid deposition in this species.

GO enrichment tests on the muscle DEGs also indicated regulatory change ($p = 0.073$) in fat cell differentiation between *D. mawsoni* and *E. maclovinus*. Adipogenesis in almost all animals is predominately regulated by peroxisome proliferator activated receptor gamma (PPARγ)(36). Expression of PPARγ in *D. mawsoni* muscle was upregulated by more than 5.6-fold compared to *E. maclovinus* (**Additional file 1: Table S14**). In addition, as many as 16 known pro-adipogenetic factors were upregulated from 1 to 8 fold. Several factors in the TGF-beta (TGFB1, smad3), Wnt (Sirt1, sirt2, frizzled-related protein (FRZB)) and Notch (jag1b, Hes1) pathways and Jun dimerization protein 2 (JDP2), reportedly negative regulators of adipogenesis were also upregulated. When compared with *N. coriiceps*, about half of the DEGs in the *D. mawsoni/E. maclovinus* comparison under this GO term were statistically insignificant, but remarkably the comparison yielded significant upregulation of 10 pro-adipogenetic factors in *D. mawsoni* muscle, including the most important regulator, PPARγ (**Fig. 5a**), and only one negative regulating factor (TGFB1) (37, 38). These results strongly suggest that regulatory promotion of adipogenesis in *D. mawsoni* muscle is a key contributing factor to fat deposition and attainment of neutral buoyancy.

Reduction of ossification in *D. mawsoni*

To reveal the genes involved in the reduced bone ossification in *D. mawsoni*, we compared the transcriptomes of pelvic girdle bones between *D. mawsoni* (0% body weight in seawater) and the negatively buoyant Antarctic notothenioids *Trematomus bernacchii* and *Pagothenia borchgrevinki*, (3.52% and 2.75% of body weight, respectively, in seawater) (14). A total of 1,733 DEGs showing the same direction of change in the *D. mawsoni/P. borchgrevinki* and the *D. mawsoni/T. bernacchii* comparisons were identified and used for further analysis (**Additional file 3**). We found that 48 genes encoding various ribosomal proteins were significantly reduced in expression in the *D. mawsoni* bone, suggesting either a lower protein translation activity or fewer metabolically active cells in the pectoral girdle than in the other two nototheniids. The DEGs were enriched with hundreds of GO biological processes, including “extracellular matrix”, “ossification”, “response to hypoxia”, “angiogenesis” and “lipid storage”, indicating multiple genetic programs were distinctly regulated in the toothfish bone (**Additional file 1: Fig. S11**). In terms of ossification, it was noteworthy that expressions of the two major regulators of vertebrate bone development, *sox9* and *runx2* (39) were not significantly altered among the three notothenioids. However, expression of many

genes of the BMP pathways, wnt pathways and many regulatory factors known to be involved in the process were specifically altered in *D. mawsoni*, which likely shifted the developmental balance between chondrogenesis and osteogenesis (**Fig. 5b; Additional file 1: Table S15**). Among these highly upregulated genes (depicted in **Fig. 5b**), CTGF (Connective Tissue Growth Factor) has been implicated in early events of osteogenic differentiation including proliferation and recruitment of osteoprogenitors, however, when expressed constitutively, CTGF would inhibit both Wnt-3A and BMP-9 induced osteoblast differentiation (40). HSPG2 (prostaglandin-endoperoxide synthase 2) is required for the chondrogenic and adipogenic differentiation from synovial mesenchymal cells via its regulation of sox9 and PPARγ, but not for osteogenic differentiation via *runx2* (41), and ECM1 (extracellular matrix protein 1) interacts with HSPG2 to regulate chondrogenesis (42). MEF2C, a transcription factor that regulates muscle and cardiovascular development, controls bone development by activating the genetic program for chondrocyte hypertrophy (43). Some of the upregulated genes are known to inhibit osteoblastogenesis, such as Tob2 (44), CTNNBIP1 (45), secreted frizzled-related protein 1 (SRFP1) (46) and ZBTB16 (47). Some members of the TGF-beta superfamily (BMPR1a, TGF-beta1, SMAD1), which were upregulated in *D. mawsoni* are known to promote both chondrogenesis and osteoblastogenesis (48). CYR61 and PTN specifically promote osteoblastogenesis (49, 50), but expression of PTN is drastically reduced, consistent with reduced hard bone formation. A few genes influence ossification via regulating osteoclastogenesis, for example, Sbno2-promotes osteoclast fusion (51) and activation of the EphA2 signaling on osteoblasts led to bone reabsorption(52). We found genes associated with osteoclast differentiation are significantly enriched in the DEGs (p <0.05) and all were upregulated (**Additional file 1: Tables S16, S17**). Overall, the gene expression patterns in the toothfish bone demonstrated a genetic shift to chondrogenesis over osteoblastogenesis in bone development, which would reduce bone density and contribute to achieving neutral buoyancy.

Studies have indicated that the majority of clinical conditions associated with human bone loss are accompanied by increased marrow adiposity possibly due to shifting of the balance between osteoblast and adipocyte differentiation in bone marrow stromal (skeletal) stem cells (53). A few signaling pathways such as the TGF-beta/BMP pathways and the Wnt pathway (represented by CTNNBIP and SFRP1 in this case) are known to participate in regulation of both bone and adipocyte development in animals. In the toothfish, we found enriched GO terms relevant to regulation of "response to lipid" and "lipid storage"

(**Additional file 1: Table S12**) indicating possible linkage in the regulatory network that orchestrates the loss of ossification and gain of lipids in *D. mawsoni* bones.

To verify whether the elevated transcription of the regulatory factors indeed resulted in more abundant protein, we selected the factor CTGF for immunohistochemical staining in the bone and surrounding tissues of pelvic fins of *D. mawsoni* and *E. maclovinus* since it is the only factor for which an effective monoclonal antibody is currently available. Much stronger signal was detected in the *D. mawsoni* fin tissue (Fig. 5c), supporting a correlation between protein abundance and mRNA transcription in the case of CTGF. This result further supports the involvement of CTGF in the reduced ossification in *D. mawsoni*.

Conclusions

We sequenced and compared the genomes and transcriptomes of the cold-adapted high-latitude Antarctic toothfish *D. mawsoni* and the basal temperate relative *E. maclovinus* representing the ancestral character state to deduce Antarctic-specific evolutionary and adaptive changes supporting physiological activities of notothenioid fishes in freezing and oxygen rich Southern Ocean waters, as well as the gain of secondary pelagicism fundamental to Antarctic notothenioid niche expansion and adaptive radiation. The assembled genomes achieved 90% (*D. mawsoni*) and 95% (*E. maclovinus*) coverage of the respective genome size estimated by cell flow cytometry, and with greater scaffold N50 than the currently available sole Antarctic notothenioid (*N. coriiceps*) genome, greatly enhancing comprehensive, genome-wide discovery of evolutionary processes.

We found two-fold expansion of TEs in the Antarctic toothfish over the temperate robalo *E. maclovinus* and deduced the timing of a burst of one major class of TEs (LINEs) to about 6.5 mya, temporally correlating with the late Miocene onset of steady cooling trend of the Southern Ocean (SO) and diversification of the modern Antarctic notothenioid clade, suggesting a role of cold-induced TE expansion in notothenioid speciation. We found many of the protein coding genes in the toothfish evolved rapidly and experienced positive selection, among which genes relevant to preservation of protein homeostasis were particularly prominent. Multiple gene families have undergone duplication during evolution in the cold, as exemplified by genes that confer resistance to freezing in the cold SO waters: the AFGP family that evolved *de novo* and confers extracellular freeze avoidance, and duplicated zona pellucida ZPC5 genes that functionally diversified to aid in cellular freezing resistance. Through transcriptome comparisons, we found functional output of the cellular apparatus for selenoprotein production in the Antarctic toothfish was greatly elevated compared to the basal

temperate robalo, suggesting evolutionary mobilization of antioxidant selenoproteins in mitigating intensified oxidative stresses arising from the O_2-rich SO environment.

The evolutionary transition from the negatively buoyant ancestral character to complete neutral buoyancy in the Antarctic toothfish entailed remarkable genetic reprograming of fat deposition and bone development. We found upregulation of processes of adipogenesis in skeletal muscle, and triacylglycerol synthesis and fat storage were favored over fatty acid oxidation. In bone development, a regulatory cascade favoring chondrogenesis over osteoblastogenesis was especially evident. The shift in fat synthesis and storage, together with reduction of ossification are therefore key in evolutionary gain of neutral buoyancy and secondary pelagicism in *D. mawsoni*, and likely in the handful of other pelagic notothenioids, allowing them to diversify into mid-water niches, a distinctive hallmark of the Antarctic notothenioid adaptive radiation.

The remarkable diversification of Antarctic notothenioids (and several other polar fish lineages) is integral to the conclusion from a recent analysis of latitudinal diversity gradient of marine fishes that high-latitude cold water lineages exhibit exceptionally high rates of speciation compared to tropical lineages, counter to expectation based on latitudinal species richness (54). Rates of molecular evolution based on phylogenetic tree branch lengths are not found to be slower at high latitudes (54). We have shown more definitively in this study that in the cold adapted Antarctic notothenioid fish, evolutionary rates in fact accelerated in thousands of protein coding genes, extensive cold-specific gene duplication and functional diversification had occurred, such as the ZP protein gene families, and TE mobility was remarkably elevated which likely contributing to the observed higher frequency of chromosomal rearrangements. In mammals, ZP3 is known to function in sperm-egg recognition (55) and TE activity is positively related to rate of speciation (56). How these genomic and functional changes elicited by selective pressures from the cold SO temperatures might have acted as intrinsic factors affecting notothenioid speciation are rich questions for further investigation.

In summary, the results of this study provided robust new insights into genomic and transcriptomic alterations enabling cold adaptation and niche expansion of the predominant and ecologically vital Antarctic fish group in the SO. The genomes also serve as valuable resources for future investigations of genomic and evolutionary changes in the diverse Antarctic notothenioid families driven by paleoclimate changes in the SO, studies that may shed light on questions of why the coldest ocean has been a hotspot of species formation.

MATERIALS AND METHODS

Specimens, sampling, and DNA and RNA isolation.

Antarctic toothfish *D. mawsoni* was collected using vertical setline through drilled hole in sea ice of McMurdo Sound, Antarctica (77° 53'S, 166° 34.4'E and vicinity) during austral summer field seasons (Oct.-Dec). Specimens were transported to the aquarium facility in the US National Science Foundation Crary Lab at McMurdo Station and kept in ambient (-1.6^{o}C) flow through seawater tanks, and sacrificed at 2-4 weeks post capture for blood and tissue sampling. The temperate basal notothenioid *E. maclovinus* was collected by rod and reel in the Patagonia waters of southern Chile during austral winter (June) and transported to the NSF R/V Laurence Gould at Punta Arenas in a large, aerated Styrofoam cooler of ambient water (~8°C), where they were sacrificed and sampled within a few days prior to southbound transit for winter field season. To obtain tissues from the large-sized *D. mawsoni*, live specimen was anesthetized with MS222 (tricaine methanesulfonate) inside a ambient seawater filled floating sheet plastic tubing in the aquarium tank. The anesthetized specimen was then put on a V-shaped trough for dissection. Tissues were quickly removed and cut into small pieces on ice, and immediately immersed and shaken in ≥10 volumes of pre-chilled (-20°C) 90% ethanol (made with 100% pure ethanol and sterilized MilliQ Type 1 water). The ethanol was replaced with a fresh volume within 10 minutes, and again at 2-3 hours and 12 hours later. This preservation method serially desiccates the tissue and effectively inactivates tissue nucleases. The tissue samples were kept in -20°C freezer throughout the serial preservation process and then stored at -20°C until use. To obtain tissues from *E. maclovinus*, MS222 anesthetized specimen was quickly dissected on ice, and preserved in -20°C as described for *D. mawsoni*. The ethanol preserved tissues were shipped back to the University of Illinois on dry ice.

RNA for transcriptome sequencing was isolated from -20°C ethanol preserved tissues using Trizol (Invitrogen) and quality verified by visualization on gel electrophoresis and an Agilent BioAnalyzer. Collection, handling and sampling of the Antarctic toothfish and S. American *E. maclovinus* in this study were carried out in compliance with protocol # 12123 approved by the University of Illinois Institutional Animal Care and Use Committee (IACUC). Additional juvenile specimens of *D, mawsoni* were collected by trawl from the waters of Antarctic Peninsula during the same winter season, and sampled on ship board shortly after capture. The dissected carcasses of *E. maclovinus* and juvenile *D. mawsoni* were kept frozen at -80°C, which provided the pelvic bone samples for immunohistochemical detection for expression of candidate

genes in bone development. To preserve high molecular weight (HMW) DNA for genome sequencing, red blood cells of each species were washed with notothenioid saline (0.1M sodium phosphate buffer, pH8.0, adjusted to 420 mOsm with NaCl for *E. maclovinus*, and 540 mOsm for *D. mawsoni*), and then embedded in 1% melt agarose to provide about 20ug DNA per 90uL block using BioRad plug molds (CHEF Mammalian Genomic DNA Plug Kit #1703591, Bio-rad, USA). The embedded cells were lysed *in situ* and the DNA in the agarose blocks was preserved following Amemiya *et al* (1996) (57). To recover HMW DNA, the agarose plugs were digested with β-agarase (NEB, USA) followed by phenol extraction and dialysis, and quality verified using pulsed field electrophoresis.

Sequencing and genome assembly

The sequencing libraries with insert sizes of 170, 250, 500 bp were prepared for sequence of the paired-end reads, following a modified version of the manufacturer's protocol (Illumina). An integrated protocol from the Mate-Pair Library v2 Sample Preparation Guide (Illumina) and the Paired-End Library Preparation Method Manual (Roche) was used to prepare mate-pair libraries with insert sizes of 3, 6, 10, 15 and 20 kb (**Additional file 1: Tables S1b, S1c**). For the transcriptome sequencing, Poly(A)+ mRNA was purified using the DynaBeads mRNA Purification kit (Life Technologies). Paired-end cDNA libraries were constructed using the RNA-Seq NGS Library Preparation Kit for Whole-Transcriptome Discovery (Gnomegen). All of the libraries are sequenced on an Illumina HiSeq 1500 sequencer. The *D. mawsoni* genome was assembled using SOAPdenovo (SOAPdenovo, RRID:SCR_010752) (18) to build the contigs and SSPACE (SSPACE, RRID:SCR_005056) (20) to scaffold the contigs. The *E. maclovinus* genome was assembled using Platanus[16] (Platanus, RRID:SCR_015531) to build the contigs, and SSPACE to scaffold the contigs.

Annotation of the genomes

We identified repeats, protein-coding genes and non-coding RNA in the genome assemblies of the two species. First, a *de novo* repeat annotation of *D. mawsoni* and *E. maclovinus* genomes was carried out by successively using RepeatModeler (RepeatModeler, RRID:SCR_015027) (version 1.0.8) and RepeatMasker (RepeatMasker, RRID:SCR_012954) (version 4.0.5). D*e novo* repeat libraries of the two species were constructed with two complementary programs, RECON (RECON, RRID:SCR_006345) (58) and RepeatScout (RepeatScout, RRID:SCR_014653) (59) implemented in the RepeatModeler package. The generated consensus sequences were manually checked by aligning to the Repbase

transposable element library (http://www.girinst.org/repbase/) and genes from the NCBI database (nt and nr). The *D. mawsoni* and *E. maclovinus* repeat library consisted of 975 and 676 consensus sequences with classification information respectively, which were used to run RepeatMasker on the assembled scaffolds. Secondly, protein-coding genes were predicted using a combination of homology-based and *de novo* approaches. GLEAN was used to create consensus gene set by integrating evidence from each prediction. Then RNA-Seq data were used to rectify gene models. Generated coding genes were aligned to known protein databases, including InterPro (60), KEGG (61) and Uniprot (62), and functional assignment was based on that of the best database match. Thirdly, the tRNA genes were predicted with tRNAscan-SE (tRNAscan-SE, RRID:SCR_010835) (63). Aligning the rRNA template sequences from fishes using BlastN (BlastN, RRID:SCR_001598) with E-value 1e-5 identified the rRNA fragments. The miRNA and snRNA genes were predicted with INFERNAL (INFERNAL, RRID:SCR_011809) (64) software against the Rfam database (Release 12) (65).

Phylogenetic reconstruction of 10 vertebrate genomes

Protein coding genes of Atlantic cod (*Gadus morhua*), tetraodon (*Tetraodon nigroviridis*), Antarctic notothenioid *N. coriiceps*, stickleback (*Gasterosteus aculeatus*), tilapia (*Oreochromis niloticus*), medaka (*Oryzias latipes*), zebrafish (*Danio rerio*) and mouse (*Mus musculus*) genomes were collected from Ensembl release 84 or NCBI, and *D. mawsoni* and *E. maclovinus* genes from this study, were used to build orthologous clusters with OrthoMCL Ver. 2.0.9 (OrthoMCL, RRID:SCR_007839)(22) with default parameters and options. A total of 2,936 one-to-one single-copy genes were identified among the ten species. Protein-coding sequences of the orthologs were aligned using PRANK (Ver.140603) (66) under a protein model with default parameters. The coding sequences of the genes were concatenated to a supergene for each species. The supergene sequence dataset was subjected to phylogenetic analysis using MrBayes (MrBayes, RRID:SCR_012067) (67), implementing best-fit substitution model (GTR+gamma+I) as determined by Modeltest (68). The analysis was run 800,000 generations, sampling every 100 generations, with the first 2,000 sample set as burn-in. Branch-specific *dN* and *dS* were estimated with codeml of the PAML package (69). The analysis of changes in gene family size were computed with CAFÉ (CAFE, RRID:SCR_005983) (70).

GO annotation and identification of positive selection genes

GO terms of the *D. mawsoni*, *E. maclovinus* and stickleback orthologs were built with InterProScan (InterProScan, RRID:SCR_005829) (71). The orthologs of each GO terms were concatenated to estimate branch-specific *dN* and *dS* using codeml of PAML (PAML, RRID:SCR_014932). A binomial test was used to identify the excess of nonsynonymous changes of GO categories in either *D. mawsoni* or *E. maclovinus* lineages referenced to the stickleback. Only the GO terms carrying more than 30 orthologs were put into this calculation. To detect genes evolving under positive selection in *D. mawsoni*, we used the branch-site model in which likelihood ratio test (LRT) *p* values were computed. Fisher's exact tests were used to test for over-represented functional categories among the positive select genes. GO enrichment analyses of the genes under positive selection were performed using a hypergeometric method.

Calling of heterozygous SNPs

All of the paired-end reads were mapped to the assembled scaffolds with the aligner SMALT (SMALT, RRID:SCR_005498) to detect the heterozygous sequence polymorphism in the genomes. The heterozygous SNPs were called with SSAHA_Pileup (version 0.8; ftp://ftp.sanger.ac.uk/pub/zn1/ssaha_pileup/). Five thresholds were used to post-filter unreliable SNPs: (1) SSAHA_Pileup SNP score >= 20; (2) ratio of two alleles between 3:17 to 17:3; (3) the lowest sequencing depth for each allele >= 5; (4) the minimum distance for adjacent SNPs >= 5 bp; (5) only one polymorphism detected at each SNP position.

Transcriptome analyses

RNA-seq data derived from liver, gill, stomach, white muscle, red muscle, skin, small intestine, brain, head kidney, caudal kidney, spleen, and ovary were analyzed for variations in gene expression of *D. mawsoni* and *E. maclovinus*. RNA-seq reads were trimmed using Trimmomatic (Trimmomatic, RRID:SCR_011848) (Ver. 0.33) (72)with the parameter set to AVGQUAL at 20, TRAILING at 20 and MINLEN at 50. The cleaned Illumina paired-end reads of each tissue were mapped to the annotated scaffolds of *D. mawsoni* and *E. maclovinus* genome using HISAT2 (HISAT2, RRID:SCR_015530) aligner (Ver. 2.0.4)(73). Cufflinks (Cufflinks, RRID:SCR_014597) (Ver. 2.2.1) (74) normalized gene expressions to the quantified transcription levels (FPKM). Differentially expressions of the genes were assessed using DEGseq (DEGseq, RRID:SCR_008480) (Ver. 1.28.0) (75)with cutoff at $q < 0.001$(76). GO and KEGG enrichment analyses for the identified differentially expressed genes were performed using cluserProfiler packages (77) with the cutoff at $p < 0.05$.

Construction of gene collinearity among *D. mawsoni*, *E. maclovinus* and stickleback genomes.

The genes of *D. mawsoni* and *E. maclovinus* were aligned to the gene model set of stickleback by Blastp (BLASTP, RRID:SCR_001010) with E-value at 1e-20. Two criteria were used to call syntenic gene blocks in the *D. mawsoni* or *E. maclovinus* scaffolds: (1) Number of the gene on the syntenic block >=3; (2) number of non-syntenic genes between two adjacent syntenic genes <= 10. Each syntenic block was anchored on the stickleback genomes according to the orders of the reference gene.

Western-blot analysis of ZP proteins

The proteins were separated on 10% SDS–PAGE at 100V for 90 min in 193 mM glycine and 25 mM Tris (pH 8.8). The resolved proteins were electrophoretically transferred to a nitrocellulose membrane (Millipore) using a Mini-Protean Tetra Cell (BioRad) in a buffer containing 193mM glycine, 25mM Tris (pH 8.3) and 20% methanol. The membrane was treated with blocking agent (5% nonfat milk in 1x TBST) for 2 h at room temperature on a shaker. FLAG antibody or β-actin antibody (Hua An Biotechnology Co. Ltd, Hangzhou, China) was added, and the membranes were incubated at room temperature for 1 h. The membrane was then washed with 1x TBST three times for 15 min each. The secondary antibody (1:2,000 in 1x TBST, Boston Biomedical Inc.) was then added and incubated for 1 h at room temperature. The membrane was washed with 1x TBST twice and 1x TBS once for 15 min each. Color was developed using SuperSignal West Pico Chemiluminescent Substrate (Thermo Scientific) according to the manufacturer's instructions. Images were acquired using a ChemiDoc MP Imaging System (BioRad).

Assay of CHO cell survival rate at freezing temperature.

DmZPC5 exons were serially deleted using PCR amplification of the DmZPC5 expression vector with primers designed to eliminate desired coding sequences (Fig.3b). The full-length sequences of three DmZPC5 isoforms (DmZPC5-1, DmZPC5-2 and DmZPC5-3) were engineered to contain a FLAG octapeptide and cloned into the expression vector pIRES2-EGFP (**Additional file 1: Fig. S5**). The three constructed vectors and blank control (vector pIRES2-EGFP) were transferred into the CHO cells (American Type Culture Collection CCL-61) obtained from American Type Culture Collection. The CHO cells were cultured in Dulbecco's modified Eagle's medium containing 10% fetal bovine serum (Gibco).

CHO cells were incubated at 37ºC for 2 days and then kept at -2 ºC for 8 hours. The treated CHO cells were collected and washed by DPBS (Dulbecco's Phosphate-Buffered Saline) twice. The cells were stained with 10 µg/mL propidium iodide at room temperature for 5 minutes, and numbers of PI-stained cells (dead cells) were determined by flow cytometry. The survival rate is calculated with the equation: survival rate = S / (S + D), where S is the number of surviving cells and D is the number the dead cells.

Identification of the LINEs and estimation of their divergence time.

The seed sequences of the LINEs (28) were aligned against the *D. mawsoni* and *E. maclovinus* draft genomes, respectively, using BlastN at E-value of 1e-10. According to loci of the alignments, the sequences were extracted from the genomes, which were considered as the candidates of the LINEs. If distance of two adjacent candidates was 200 bp or less, these two candidates were connected by the sequence between them. All of the candidates and their corresponding seed sequences were mutually aligned by BlastN. Those candidates with over 60% identity and over 100 bp of alignments to the seed sequences were collected as the LINEs of *D. mawsoni* and *E. maclovinus*, respectively.

Alignment of the LINEs was conducted by a multiple sequence aligner ClustalW (ClustalW, RRID:SCR_002909) (78) to cluster any two LINEs with highest sequence similarity into a LINE pair in *D. mawsoni* or *E. maclovinus*. The evolutionary distance of two LINES for each LINE pair was calculated by the Kimura two-parameter method (EMBOSS distmat, version 6.6.0.0), which reflected the substitution rate per site between the two LINEs. According to the calculated synonymous substitution rates for 7,958 *D. mawsoni*-*E. maclovinus* orthologous pairs, the mean synonymous substitution rate is around 0.227. The peak of substitution rate is at 0.04, which estimated the LINE burst to be about 6.5 million years ago when the species divergence time between *D. mawsoni* and *E. maclovinus* is around 37 million years ago (79).

Tissue fixation and immunohistochemistry

Pieces of pelvic bone (with muscle) and the attached fins (no larger than 20 × 20 × 5 mm) were dissected from frozen specimens of young *D. mawsoni* and *E. maclovinus* and immersed in a fixation solution, KINFix which contains (62.5% (v/v) ethanol, 6.71% (v/v) acetic acid, and 6% (w/v) trehalose (80) for over 24hrs. Tissue are decalcified in EDTA solution（cat. no.041-22031, WAKO）for about 2 weeks. Then the specimen was dehydrated in graded

ethanol（70%, 80%, 90%,95%, 1×1h each), 100% ethanol 2 ×1h at room temperature, xylene for 2×1h, and embedded in low-melting paraffin for 2 × 1h, and kept overnight at 56°C, then embedded in paraffin. For each tissue, 5 µm thick serial sections were cut with a microtome (RM2245, Leica). Immunostaining was performed using the EnVision detection system (cat. no.K5007, Dako). Slides were deparafinized in xylene and rehydrated in a descending series of ethanol (100%, 95%, 90% and 70%), and washed in phosphate buffered saline (PBS). Endogenous peroxidases were blocked with 3% H_2O_2 for 10 min, after which the sections were incubated with 5% BSA for 35min. Then, the slides are incubated overnight with the primary CTGF antibody (1:400 dilution) (cat. no.ab6992, Abcam) at 4°C. Next, the sections were washed four times with PBS for 15 min followed by incubation with a goat anti-rabbit secondary antibody for 35min at 37°C. After four washes with PBS, 3, 3'-diaminobenzidine (DAB) was added to visualize the immunoreactivity. All slides were counterstained with haematoxylin. The sections were dehydrated in a mounting series of alcohol (70%, 90%, 100% and 100%) and in xylene. Finally, slides are mounted using neutral balsam mounting medium, and analyzed under a bright field microscope (AXIO imager. M2, ZEISS).

Availability of data and material

All of the Illumina short read sequencing data of this project have been deposited at NCBI under the accession no. BioProject PRJNA401363 (http://www.ncbi.nlm.nih.gov/sra/). The assembled draft genomes and their annotations have been released at the official website of the Shanghai Ocean University (http://202.121.66.128/). The current version of the data set is the first version (v1). Antarctic toothfish genome and transcriptome (86), Patagonian robalo genome and transcriptome (87) and other supporting data are also available via the *GigaScience* GigaDB repository(88).

Abbreviations

ACC: Antarctic Circumpolar Current; AFGP: antifreeze glycoprotein; AGPAT: acylglycerol-3-phosphate O-acyltransferase; BUSCO: Benchmarking Universal Single-Copy Orthologs; CDS: CDP-diacylglycerol synthase; CHO: Chinese Hamster Ovary; CTGF: Connective Tissue Growth Factor; DEG: differentially expressed genes; DPBS: Dulbecco's Phosphate-Buffered Saline; ECM1: extracellular matrix protein 1; GO: Gene Ontology; HMW: high molecular weight; HSPG2: prostaglandin-endoperoxide synthase 2; IACUC:

Institutional Animal Care and Use Committee; JDP2: Jun dimerization protein 2; LINE: long interspersed transposable elements; LRT: likelihood ratio test; PBS: phosphate buffered saline; PPAR:peroxisome proliferator-activated receptors; PPARγ: peroxisome proliferator activated receptor gamma; PSG: positively selected genes; SINE: short interspersed transposable elements; SO: Southern Ocean; SRFP1: secreted frizzled-related protein 1; SSR: simple sequence repeats; TE: Transposable elements; ZP: Zona Pellucida protein.

Additional files

Additional file 1 : Figs. S1 to S11 and Tables S1 to S17.

Additional file 2 : supporting data for Fig. 1b, Fig.2c, Fig. 2d, and Fig. 4.

Additional file 3 : list of duplicated protein gene families of *D. mawsoni* and *E. maclovinus.*

Additional file 4: list of DEGs between *D.mawson*i and two negatively buoyant notothenioids.

Acknowledgements

We thank BGI-Shenzhen for their technical support in sequencing of *D. mawsoni* genome, Mr. Liping Shu of Wuhan Ice-harbor Biological Technological Co. Ltd. for his help in the annotation of the *E. maclovinus* genome.

Funds

The work was supported by grants from the Natural Science Foundation of China (No. 41761134050, 31572611, 31572598) and the Major Science Innovation Grant (2017-01-07-00-10-E00060) from the Shanghai Education Committee, the Key Achievement Supporting Grant from Laboratory for Marine Biology and Biotechnology, Qingdao National Laboratory for Marine Science and Technology to LC, and the USA NSF Polar Programs grant ANT1142158 to CHCC.

Authors' contribution

LC and CHCC conceived and managed the project and its components. CHCC and MH performed fish and tissue collections and sample preparations. KRM and XZ contributed to DNA and RNA preparations and genome size determination. YL, MY, and WL performed genome annotation and RNA-seq data analysis. YR and SP conducted *de novo* genome assembly. KTB confirmed the AFGP loci. QX, YF and LC designed and performed the biological experiments. Sample preparation and genome sequencing were carried out by SJ,

WZ and JW. LC, QX, YL and CHCC analyzed the data as a whole and wrote the manuscript, and CHCC and WW contributed interpretation of data and edits to the manuscript.

Competing interests

The authors declare they have no competing financial interests.

URLs KEGG, http://www.genome.jp/kegg/; KAAS, http://www.genome.jp/tools/kaas/;SSPACE, https://www.baseclear.com/genomics/bioinformatics/basetools/SSPACE; Platanus, http://platanus.bio.titech.ac.jp/; SMALT, http://www.sanger.ac.uk/resources/software/smalt/; SOAPdenovo, http://soap.genomics.org.cn; RepeatModeler, http://www.repeatmasker.org/RepeatModeler.html; RepeatMasker, http://www.repeatmasker.org; Repbase, http://www.girinst.org/repbase/; Timetree, http://www.timetree.org/;Ensembl, ftp://ftp.ensembl.org/pub/; http://evolution.genetics.washington.edu/phylip.html; PAML, http://abacus.gene.ucl.ac.uk/software/paml.html; GLEAN, https://github.com/glean/glean; Interpro, http://www.ebi.ac.uk/interpro/; Infernal, http://eddylab.org/infernal/; Rfam, http://rfam.xfam.org/; OrthoMCL, http://orthomcl.org/orthomcl/; Mrbayes, http://mrbayes.sourceforge.net/; SSAHA_Pileup, ftp://ftp.sanger.ac.uk/pub/zn1/ssaha_pileup/; HISAT2, http://ccb.jhu.edu/software/hisat2/index.shtml.

Legends

Fig. 1 Sequenced species and genome synteny. (a) Sampling location and geographic distribution. The red and blue filled circles are the geographic distributions of *D. mawsoni* and *E. maclovinus*, respectively (www.fishbase.org, version 02/2018 (81) and Hanchet et al.(82). The red and blue stars show the respective locations where the sequenced individuals were collected. The Antarctic Polar Front, an approximation of the mean position of the Antarctic Circumpolar Current, is adopted from Barker and Thomas(83). The image of *D. mawsoni* is a courtesy from Elliot DeVries and that of *E. maclovinus* is from Dirk Schories. (**b**) Gene collinearity among *D. mawsoni*, *E. maclovinus* and *G. aculeatus* (Stickleback). The scaffolds of *D. mawsoni* (the

circularized red blocks labelled with "D") and *E. maclovinus* (the circularized green blocks labelled with "E") are anchored on the twenty-one Stickleback chromosomes (the circularized light blue blocks labelled with "G", 1 to 21), according to the gene collinearity (the connecting yellow lines). The black vertical lines within the *D. mawsoni* and *E. maclovinus* scaffolds indicate occurrence of LINEs greater than 500 bps in these positions. The sequence length is indicated by the 5-Mb tick marks on the reference Stickleback chromosomes. The outermost circle of red vertical lines and the innermost circle of green vertical lines indicated the quantified expression levels (FPKM) of the genes located on the corresponding *D. mawsoni* and *E. maclovinus* scaffolds, respectively. The expression profiles are derived from the transcriptome data of white muscles (see the transcriptome section). The small white squares and rectangles scattered in the scaffolds show the locations of the Selcys-tRNA genes of *D. mawsoni* and *E. maclovinus*. The single yellow square shows the location of AFGP genes in the *D. mawsoni* genome.

Fig. 2 Evolution of the genomes and genes. (**a**) Timing and frequency of LINE insertion in *D. mawsoni*, *E. maclovinus* and *N. coriiceps* showing correlation between onset of late Miocene deep cooling and burst LINE insertions in the Antarctic toothfish and bullhead notothen. The black trace indicates global temperature trends during Oligocene, Miocene, Pliocene (Pli) and Pleistocene (Ple) from 30 to 0 million years ago (mya), modified from Zachos *et al.* (2008)(84), Near *et al.* (2012) (9)and Favre *et al.* (2015) (85). The red and blue line indicate the insertion frequency of LINEs (the percentage of the calculated LINE pairs) in the *D. mawsoni* and *E. maclovinus* genomes respectively during these periods. **(b)** Reconstructed phylogeny of nine teleost fish lineages using 2,936 orthologous genes (mouse serving as outgroup) and the calculated *dN*/*dS* ratio for each branch, showing a 2-fold faster evolutionary rate in the Antarctic notothenioids. (**c**) Comparison of adaptive evolution between *D. mawsoni* and *E. maclovinus* genomes. Data points represent average *dN*/*dS* value of each GO term, each of which consists of at least 30 genes. The red and blue circles show the GO terms with significantly higher *dN*/*dS* ratios ($p < 0.05$, binomial test) in *D. mawsoni* and *E. maclovinus*, respectively. The grey circles are those showing no significant difference. GO terms falling on the dashed line of linearity have the same *dN*/*dS* ratios in the two species. (**d**) Gene duplication in *D. mawsoni*. A subset (26) of the 202 gene families detected to contain higher gene copy numbers in the *D. mawsoni* genome relative to other species are listed on the left, with their respective KEGG pathway listed on the right. The gene copy numbers are measured by color difference. The pathways highlighted in red are especially abundant in *D. mawsoni*

and might be relevant to physiological adaptation of *D. mawsoni* in the freezing environment. (**e**) A subset of duplicated gene families in *E. maclovinus*, showing different KEGG pathways between *D. mawsoni* and *E. maclovinus* in terms of gene duplication. The red highlighted pathway (ether lipid metabolism) indicates a common duplication occurred in the three Notothenioids.

Fig. 3 Evolutionary and functional analyses of the DmZPC5 genes involved in cellular freezing resistance. (**a**) Duplication of ZPC5 gene (DmZPC5) in *D. mawsoni*. Phylogenetic neighbor-joining tree of ZPC5 genes among *D. mawsoni*, *E. maclovinus*, *Larimichthys crocea* (Lc), *T. rubripes* (Tr) and *O. latipes* (Ol). The gene structures are illustrated on the right. The different colored blocks indicate the exons encoding signal peptides (red), zp domains (blue) and the remaining exons (incarnadine). The jagged blocks contain the nonsense mutations in DmZPC5-2a/b and DmZPC5-3 genes that cause premature termination of coding sequences. (**b**) Western-blot analysis of the DmZPC5 isoforms indicated their sizes and temperature sensitive accumulation. Purified proteins encoded by the three DmZPC5 isoforms were detected by an anti-FLAG antibody on the SDS-PAGE gels. All of these three DmZPC5 protein had higher expression levels at 0°C than at 37 $^{\circ}$C. (**c**) Assays of cell survival rate under recombinant expression of different DmZPC5 isoforms in CHO cells at a freezing temperature (-2°C for 8 hrs). The bars represent the mean ± s.d (n =3, biological replicates). The sample pIRES2-EGFP is the expression vector as control. Significances of different survival rate are indicated by * (unpaired Student's t-test, $p < 0.05$) and ** ($p < 0.01$).

Fig. 4. Comparison of gene expression between *D. mawsoni* and *E. maclovinus* tissues. The squares/triangles, circles and diamonds filled in different colors represented the genes involved in three metabolic processes (listed on the right). The genes with significantly higher expression in *D. mawsoni* or *E. maclovinus* are labeled on the corresponding organs.

Fig. 5 Schematic diagram showing changed regulation of buoyancy related developmental pathways**.** (**a**). Enhanced adipogenetic pathways in *D. mawsoni* muscle. The genes shadowed with the dark red color were upregulated in *D. mawsoni* while those shadowed in light grey were unchanged. (**b**). Changed osteogenetic regulation in *D. mawsoni* bone. Genes shadowed with the dark grey color were upregulated in *D. mawsoni* while those in light grey were not

changed. The arrows (in dark red or dark grey) indicate a positive effect on the process while blocked (in blue) lines indicate inhibitory effect. MSC: Mesenchymal Stem Cell. (**c**). Immunohistochemical (IHC) staining to detect the abundance of Connective Tissue Growth Factor (CTGF) in cross sections of pelvic fin of *D. mawsoni* and *E. maclovinus*. The left panels of each fish are IHC staining without the first antibody as negative control. The presence of CTGF is indicated by the brown signals in the right tissues. Scale bar, 50μm.

References

1. Livermore R, Nankivell A, Eagles G, & Morris P (2005) Paleogene opening of Drake Passage. *Earth Planet Sc Lett* 236(1-2):459-470.
2. Eastman JT (2005) The nature of the diversity of Antarctic fishes. *Polar Biol* 28(2):93-107.
3. Near TJ*, et al.* (2015) Identification of the notothenioid sister lineage illuminates the biogeographic history of an Antarctic adaptive radiation. *Bmc Evol Biol* 15:109.
4. Eastman JT (2000) Antarctic notothenioid fishes as subjects for research in evolutionary biology. *Antarct Sci* 12(3):276-287.
5. La Mesa M, Eastman JT, & Vacchi M (2004) The role of notothenioid fish in the food web of the Ross Sea shelf waters: a review. *Polar Biol* 27(6):321-338.
6. Devries AL & Eastman JT (1978) Lipid sacs as a buoyancy adaptation in an Antarctic fish. *Nature* 271(5643):352-353.
7. Chen LB, DeVries AL, & Cheng CHC (1997) Evolution of antifreeze glycoprotein gene from a trypsinogen gene in Antarctic notothenioid fish. *P Natl Acad Sci USA* 94(8):3811-3816.
8. Matschiner M, Hanel R, & Salzburger W (2011) On the Origin and Trigger of the Notothenioid Adaptive Radiation. *Plos One* 6(4).
9. Near TJ*, et al.* (2012) Ancient climate change, antifreeze, and the evolutionary diversification of Antarctic fishes. *P Natl Acad Sci USA* 109(9):3434-3439.
10. Shin SC*, et al.* (2014) The genome sequence of the Antarctic bullhead notothen reveals evolutionary adaptations to a cold environment. *Genome Biol* 15(9).
11. Malmstrom M*, et al.* (2016) Evolution of the immune system influences speciation rates in teleost fishes. *Nat Genet* 48(10):1204-1210.
12. Gon O & Heemstra P (1990) *Fishes of the Southern Ocean* (J.L.B. Smith Institute of Ichthyology) p 305.
13. Eastman JT, Witmer LM, Ridgely RC, & Kuhn KL (2014) Divergence in Skeletal Mass and Bone Morphology in Antarctic Notothenioid Fishes. *J Morphol* 275(8):841-861.
14. Eastman JT & Devries AL (2010) Buoyancy adaptations in a swim ‐ bladderless Antarctic fish. *J Morphol* 167(1):91-102.
15. Eastman JT & Devries AL (1982) Buoyancy Studies of Notothenioid Fishes in McMurdo Sound, Antarctica. *Copeia* 1982(2):385-393.
16. Ghigliotti L*, et al.* (2007) The two giant sister species of the Southern Ocean, Dissostichus eleginoides and Dissostichus mawsoni, differ in karyotype and chromosomal pattern of ribosomal RNA genes. *Polar Biol* 30(5):625-634.
17. Mazzei F*, et al.* (2008) Chromosomal characteristics of the temperate notothenioid fish Eleginops maclovinus (Cuvier). *Polar Biol* 31(5):629-634.
18. Li RQ, Li YR, Kristiansen K, & Wang J (2008) SOAP: short oligonucleotide alignment program. *Bioinformatics* 24(5):713-714.
19. Kajitani R*, et al.* (2014) Efficient de novo assembly of highly heterozygous genomes from whole-genome shotgun short reads. *Genome Res* 24(8):1384-1395.
20. Boetzer M, Henkel CV, Jansen HJ, Butler D, & Pirovano W (2011) Scaffolding pre-assembled contigs using SSPACE. *Bioinformatics* 27(4):578-579.
21. Simã£O FA, Waterhouse RM, Ioannidis P, Kriventseva EV, & Zdobnov EM (2015) BUSCO: assessing genome assembly and annotation completeness with single-copy orthologs. *Bioinformatics* 31(19):3210-3212.

22. Li L, Stoeckert CJ, & Roos DS (2003) OrthoMCL: Identification of ortholog groups for eukaryotic genomes. *Genome Res* 13(9):2178-2189.

23. Pisano E & Ozouf-Costaz C (2003) Cytogenetics and evolution in extreme environment: the case of Antarctic fishes. *Science Publishers Inc, Enfield (NH) USA*:309-330.

24. Detrich HW*, et al.* (2010) Genome Enablement of the Notothenioidei: Genome Size Estimates from 11 Species and BAC Libraries from 2 Representative Taxa. *J Exp Zool Part B* 314b(5):369-381.

25. Rebollo R, Horard B, Hubert B, & Vieira C (2010) Jumping genes and epigenetics: Towards new species. *Gene* 454(1-2):1-7.

26. Auvinet J*, et al.* (2018) Mobilization of retrotransposons as a cause of chromosomal diversification and rapid speciation: the case for the Antarctic teleost genus Trematomus. *Bmc Genomics* 19.

27. Chenais B, Caruso A, Hiard S, & Casse N (2012) The impact of transposable elements on eukaryotic genomes: From genome size increase to genetic adaptation to stressful environments. *Gene* 509(1):7-15.

28. Chen S*, et al.* (2017) Cold-induced retrotransposition of fish LINEs. *Journal of genetics and genomics = Yi chuan xue bao* 44(8):385-394.

29. Chen ZZ*, et al.* (2008) Transcrintomic and genomic evolution under constant cold in Antarctic notothenioid fish. *P Natl Acad Sci USA* 105(35):12944-12949.

30. Xu QH*, et al.* (2008) Adaptive evolution of hepcidin genes in antarctic notothenioid fishes. *Mol Biol Evol* 25(6):1099-1112.

31. Cao LX*, et al.* (2016) Neofunctionalization of zona pellucida proteins enhances freeze-prevention in the eggs of Antarctic notothenioids. *Nat Commun* 7.

32. Nicodemus-Johnson J, Silic S, Ghigliotti L, Pisano E, & Cheng CHC (2011) Assembly of the antifreeze glycoprotein/trypsinogen-like protease genomic locus in the Antarctic toothfish Dissostichus mawsoni (Norman). *Genomics* 98(3):194-201.

33. Brigelius-Flohe R & Maiorino M (2013) Glutathione peroxidases. *Bba-Gen Subjects* 1830(5):3289-3303.

34. Angeli JPF*, et al.* (2014) Inactivation of the ferroptosis regulator Gpx4 triggers acute renal failure in mice. *Nat Cell Biol* 16(12):1180-U1120.

35. Chen Y, Rui BB, Tang LY, & Hu CM (2015) Lipin Family Proteins - Key Regulators in Lipid Metabolism. *Ann Nutr Metab* 66(1):10-18.

36. Farmer SR (2006) Transcriptional control of adipocyte formation. *Cell Metab* 4(4):263-273.

37. Ignotz RA & Massagué J (1985) Type beta transforming growth factor controls the adipogenic differentiation of 3T3 fibroblasts. *P Natl Acad Sci USA* 82(24):8530-8534.

38. Petruschke T, Röhrig K, & Hauner H (1994) Transforming growth factor beta (TGF-beta) inhibits the differentiation of human adipocyte precursor cells in primary culture. *Int J Obes Relat Metab Disord* 18(8):532-536.

39. Long FX & Ornitz DM (2013) Development of the Endochondral Skeleton. *Csh Perspect Biol* 5(1).

40. Luo Q*, et al.* (2004) Connective tissue growth factor (CTGF) is regulated by Wnt and bone morphogenetic proteins signaling in osteoblast differentiation of mesenchymal stem cells. *J Biol Chem* 279(53):55958-55968.

41. Sadatsuki R*, et al.* (2017) Perlecan is required for the chondrogenic differentiation of synovial

mesenchymal cells through regulation of Sox9 gene expression. *J Orthop Res* 35(4):837-846.

42. Mongiat M*, et al.* (2003) Perlecan protein core interacts with extracellular matrix protein 1 (ECM1), a glycoprotein involved in bone formation and angiogenesis. *J Biol Chem* 278(19):17491-17499.

43. Arnold MA*, et al.* (2007) MEF2C transcription factor controls chondrocyte hypertrophy and bone development. *Dev Cell* 12(3):377-389.

44. Gamez B, Rodriguez-Carballo E, Bartrons R, Rosa JL, & Ventura F (2013) MicroRNA-322 (miR-322) and Its Target Protein Tob2 Modulate Osterix (Osx) mRNA Stability. *J Biol Chem* 288(20):14264-14275.

45. Tago K*, et al.* (2000) Inhibition of Wnt signaling by ICAT, a novel beta-catenin-interacting protein. *Gene Dev* 14(14):1741-1749.

46. Yao W*, et al.* (2010) Overexpression of Secreted Frizzled-Related Protein 1 Inhibits Bone Formation and Attenuates Parathyroid Hormone Bone Anabolic Effects. *J Bone Miner Res* 25(2):190-199.

47. Onizuka S*, et al.* (2016) ZBTB16 as a Downstream Target Gene of Osterix Regulates Osteoblastogenesis of Human Multipotent Mesenchymal Stromal Cells. *J Cell Biochem* 117(10):2423-2434.

48. Dexheimer V*, et al.* (2016) Differential expression of TGF-beta superfamily members and role of Smad1/5/9-signalling in chondral versus endochondral chondrocyte differentiation. *Sci Rep-Uk* 6.

49. Tare RS, Oreffo ROC, Clarke NMP, & Roach HI (2002) Pleiotrophin/osteoblast-stimulating factor 1: Dissecting its diverse functions in bone formation. *J Bone Miner Res* 17(11):2009-2020.

50. Su JL*, et al.* (2010) CYR61 Regulates BMP-2-dependent Osteoblast Differentiation through the alpha(v)beta(3) Integrin/Integrin-linked Kinase/ERK Pathway. *J Biol Chem* 285(41):31325-31336.

51. Maruyama K*, et al.* (2013) Strawberry notch homologue 2 regulates osteoclast fusion by enhancing the expression of DC-STAMP. *J Exp Med* 210(10):1947-1960.

52. Diercke K, Sen S, Kohl A, Lux CJ, & Erber R (2011) Compression-dependent Up-regulation of Ephrin-A2 in PDL Fibroblasts Attenuates Osteogenesis. *J Dent Res* 90(9):1108-1115.

53. Abdallah BM & Kassem M (2012) New factors controlling the balance between osteoblastogenesis and adipogenesis. *Bone* 50(2):540-545.

54. Rabosky DL*, et al.* (2018) An inverse latitudinal gradient in speciation rate for marine fishes. *Nature* 559(7714):392-395.

55. Litscher ES, Williams Z, & Wassarman PM (2009) Zona Pellucida Glycoprotein ZP3 and Fertilization in Mammals. *Mol Reprod Dev* 76(10):933-941.

56. Ricci M, Peona V, Guichard E, Taccioli C, & Boattini A (2018) Transposable Elements Activity is Positively Related to Rate of Speciation in Mammals. *Journal of Molecular Evolution* 86(5):303-310.

57. Amemiya CT, Ota T, & Litman GW (1996) Construction of P1 Artificial Chromosome (PAC) Libraries from Lower Vertebrates. *Nonmammalian Genomic Analysis*, (San Diego: Academic Press), pp 223-256.

58. Bao ZR & Eddy SR (2002) Automated de novo identification of repeat sequence families in sequenced genomes. *Genome Res* 12(8):1269-1276.

59. Price AL, Jones NC, & Pevzner PA (2005) De novo identification of repeat families in large genomes. *Bioinformatics* 21:I351-I358.
60. Apweiler R*, et al.* (2001) The InterPro database, an integrated documentation resource for protein families, domains and functional sites. *Nucleic Acids Res* 29(1):37-40.
61. Kanehisa M & Goto S (2000) KEGG: Kyoto Encyclopedia of Genes and Genomes. *Nucleic Acids Res* 28(1):27-30.
62. Bairoch A*, et al.* (2005) The universal protein resource (UniProt). *Nucleic Acids Res* 33:D154-D159.
63. Lowe TM & Eddy SR (1997) tRNAscan-SE: A program for improved detection of transfer RNA genes in genomic sequence. *Nucleic Acids Res* 25(5):955-964.
64. Nawrocki EP, Kolbe DL, & Eddy SR (2009) Infernal 1.0: inference of RNA alignments. *Bioinformatics* 25(10):1335-1337.
65. Nawrocki EP*, et al.* (2015) Rfam 12.0: updates to the RNA families database. *Nucleic Acids Res* 43(D1):D130-D137.
66. Loytynoja A & Goldman N (2010) webPRANK: a phylogeny-aware multiple sequence aligner with interactive alignment browser. *Bmc Bioinformatics* 11.
67. Huelsenbeck JP & Ronquist F (2001) MRBAYES: Bayesian inference of phylogenetic trees. *Bioinformatics* 17(8):754-755.
68. Posada D & Crandall KA (1998) MODELTEST: testing the model of DNA substitution. *Bioinformatics* 14(9):817-818.
69. Yang ZH (2007) PAML 4: Phylogenetic analysis by maximum likelihood. *Mol Biol Evol* 24(8):1586-1591.
70. De Bie T, Cristianini N, Demuth JP, & Hahn MW (2006) CAFE: a computational tool for the study of gene family evolution. *Bioinformatics* 22(10):1269-1271.
71. Zdobnov EM & Apweiler R (2001) InterProScan - an integration platform for the signature-recognition methods in InterPro. *Bioinformatics* 17(9):847-848.
72. Bolger AM, Lohse M, & Usadel B (2014) Trimmomatic: a flexible trimmer for Illumina sequence data. *Bioinformatics* 30(15):2114-2120.
73. Kim D, Landmead B, & Salzberg SL (2015) HISAT: a fast spliced aligner with low memory requirements. *Nat Methods* 12(4):357-U121.
74. Trapnell C*, et al.* (2012) Differential gene and transcript expression analysis of RNA-seq experiments with TopHat and Cufflinks. *Nat Protoc* 7(3):562-578.
75. Wang LK, Feng ZX, Wang X, Wang XW, & Zhang XG (2010) DEGseq: an R package for identifying differentially expressed genes from RNA-seq data. *Bioinformatics* 26(1):136-138.
76. Benjamini Y & Hochberg Y (1995) Controlling the False Discovery Rate - a Practical and Powerful Approach to Multiple Testing. *J Roy Stat Soc B Met* 57(1):289-300.
77. Yu GC, Wang LG, Han YY, & He QY (2012) clusterProfiler: an R Package for Comparing Biological Themes Among Gene Clusters. *Omics* 16(5):284-287.
78. Thompson JD, Higgins DG, & Gibson TJ (1994) CLUSTAL W: improving the sensitivity of progressive multiple sequence alignment through sequence weighting, position-specific gap penalties and weight matrix choice. *Nucleic Acids Res*:4673–4680.
79. Hedges SB, Dudley J, & Kumar S (2006) TimeTree: a public knowledge-base of divergence times among organisms. *Bioinformatics* 22(23):2971-2972.
80. Stefanits H*, et al.* (2016) KINFix - A formalin-free non-commercial fixative optimized for

histological, immunohistochemical and molecular analyses of neurosurgical tissue specimens. *Clin Neuropathol* 35(1):3-12.

81. Pauly FRaD (2018) FishBase. in *World Wide Web electronic publication*.
82. Hanchet S*, et al.* (2015) The Antarctic toothfish (Dissostichus mawsoni): biology, ecology, and life history in the Ross Sea region. *Hydrobiologia* 761(1):397-414.
83. Barker PF & Thomas E (2004) Origin, signature and palaeoclimatic influence of the Antarctic Circumpolar Current. *Earth-Sci Rev* 66(1-2):143-162.
84. Zachos JC, Dickens GR, & Zeebe RE (2008) An early Cenozoic perspective on greenhouse warming and carbon-cycle dynamics. *Nature* 451(7176):279-283.
85. Favre A*, et al.* (2015) The role of the uplift of the Qinghai-Tibetan Plateau for the evolution of Tibetan biotas. *Biol Rev* 90(1):236-253.
86. Chen L; Lu Y; Li W; Ren Y; Yu M; Jiang S; Fu Y; Wang J; Peng S; Bilyk KT; Murphy KR; Zhuang X; Hune M; Zhai W; Wang W; Xu Q; Cheng CC (2019): The genome and transcriptome of the Antarctic toothfish *Dissostichus mawsoni* GigaScience Database. http://dx.doi.org/10.5524/102162
87. Chen L; Lu Y; Li W; Ren Y; Yu M; Jiang S; Fu Y; Wang J; Peng S; Bilyk KT; Murphy KR; Zhuang X; Hune M; Zhai W; Wang W; Xu Q; Cheng CC (2019): The genome and transcriptome of the Patagonia robalo *Eleginops maclovinus* GigaScience Database. http://dx.doi.org/10.5524/102163
88. Chen L; Lu Y; Li W; Ren Y; Yu M; Jiang S; Fu Y; Wang J; Peng S; Bilyk KT; Murphy KR; Zhuang X; Hune M; Zhai W; Wang W; Xu Q; Cheng CC (2019): Supporting data for "Genomic bases for colonizing the freezing Southern Ocean revealed by the genomes of Antarctic toothfish and Patagonia robalo" GigaScience Database. http://dx.doi.org/10.5524/100545

Table 1 Overview of assembly and annotation

	D. mawsoni	*E. maclovinus*
Assembly		
Total length (Mb)	756.8	744.4
Contig N50 length (Kb)	23.1	10.9
Scaffold N50 length (Kb)	2,216.2	694.7
Scaffold N90 length (Kb)	202.7	167.2
Largest scaffold (Mb)	13.8	4.9
Quantity of scaffolds (>N90 length)	536	1,185
Annotation		
Quantity of predicted protein-coding genes	22,516	22,959
Quantity of predicted non-coding RNA genes	2,434	2,185
Content of transposable elements	21.38%	10.02%
Heterozygous SNP rate (SNPs per kb)	2.58	2.40

Fig.1

Click here to access/download;Figure;Fig.1.tif

a

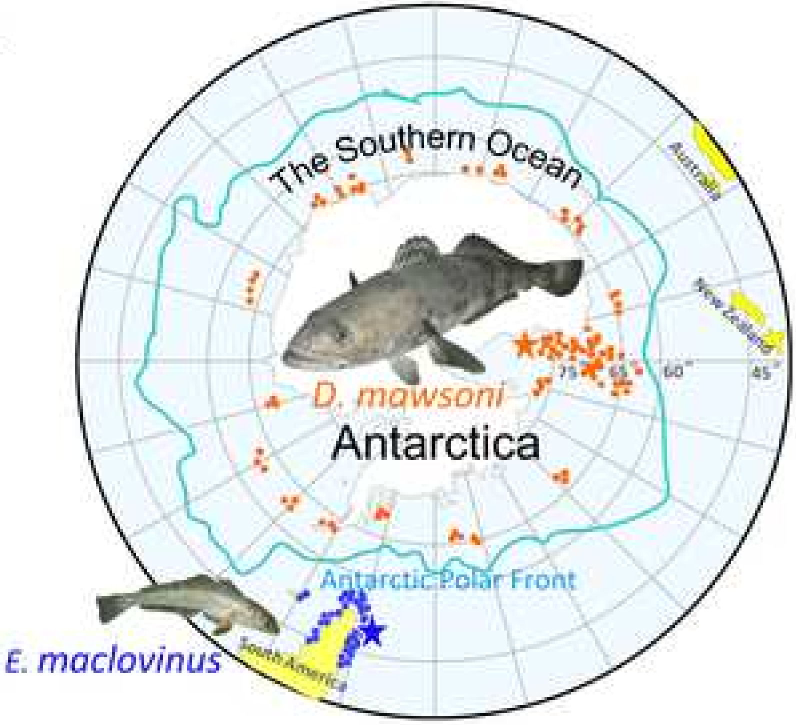

b

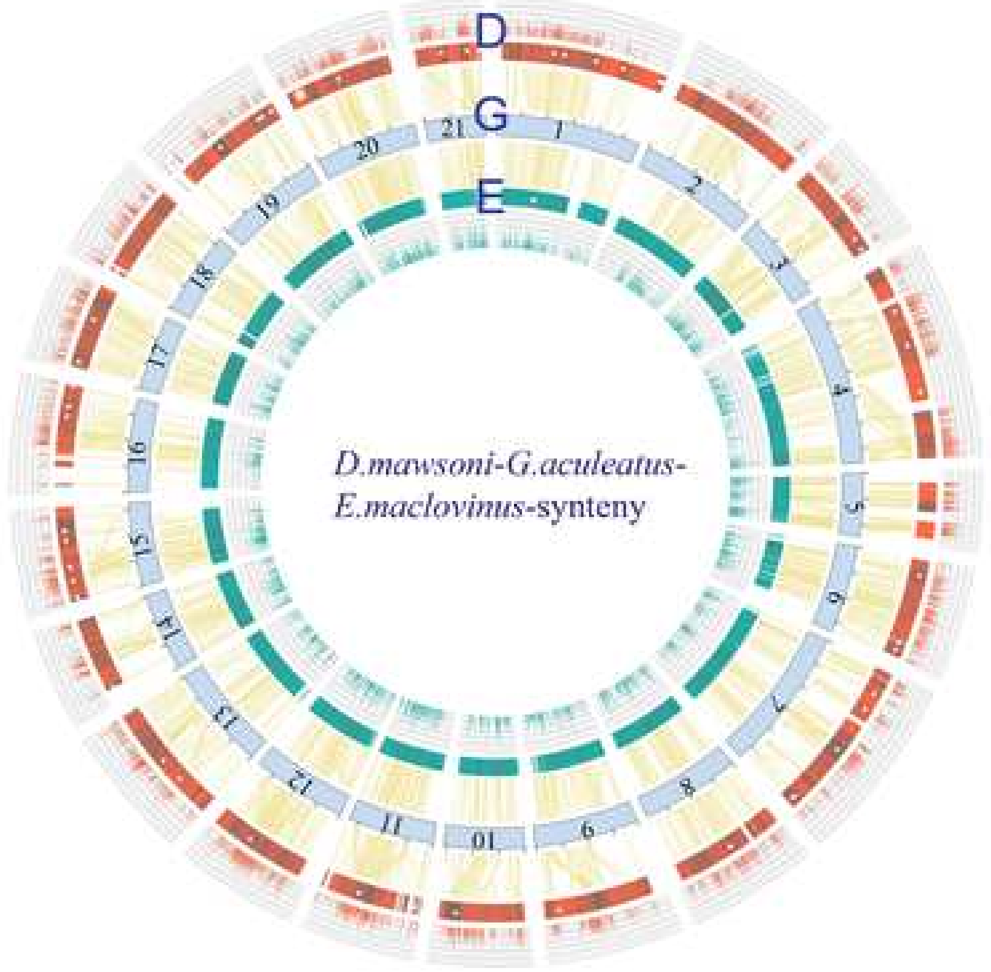

Click here to access/download;Figure;Fig.2_revised26Nov2018.tif

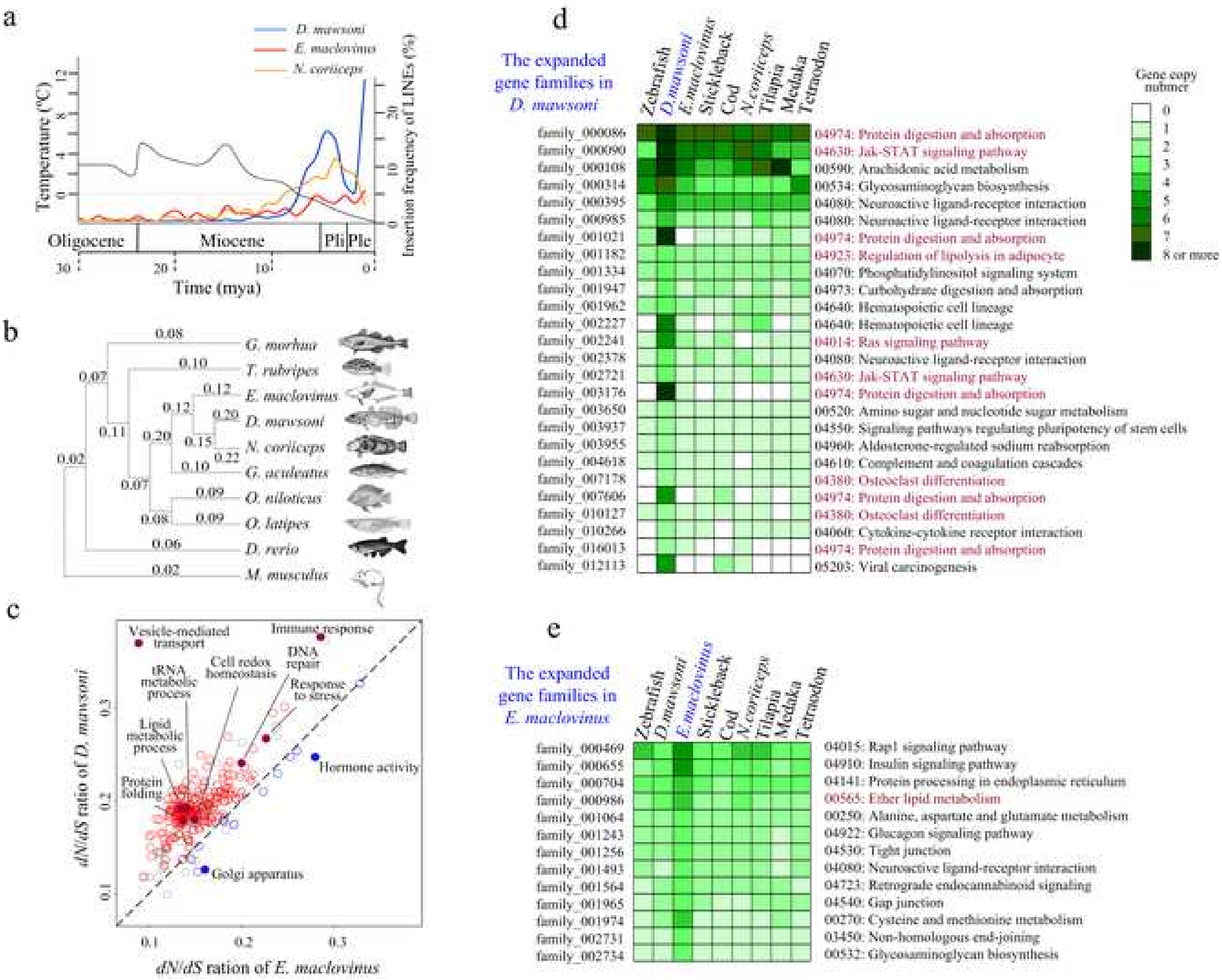

Click here to access/download;Figure;Fig.3.tif

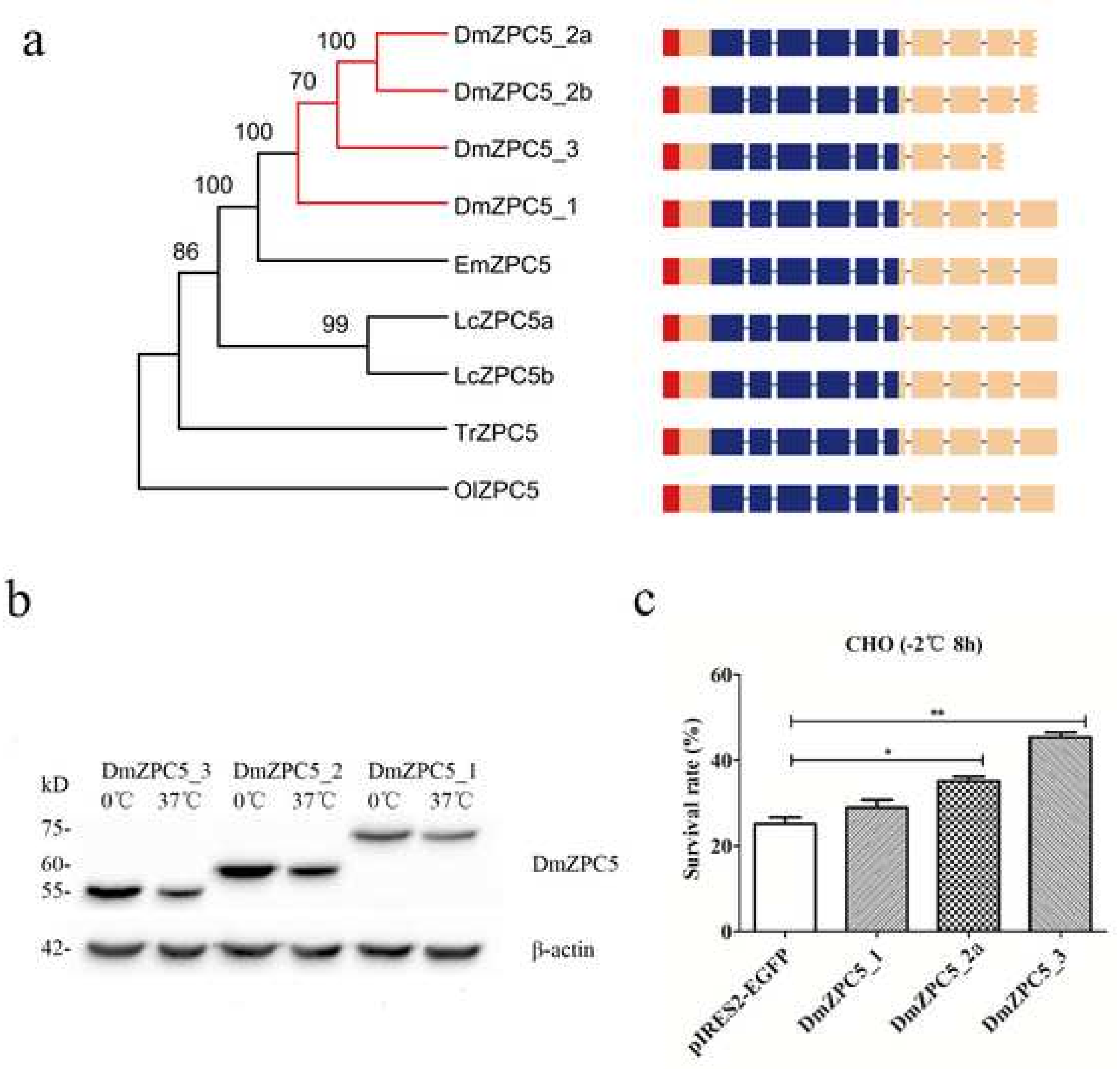

Fig.4

Click here to access/download;Figure;Fig.4_revised26Nov2018.tif

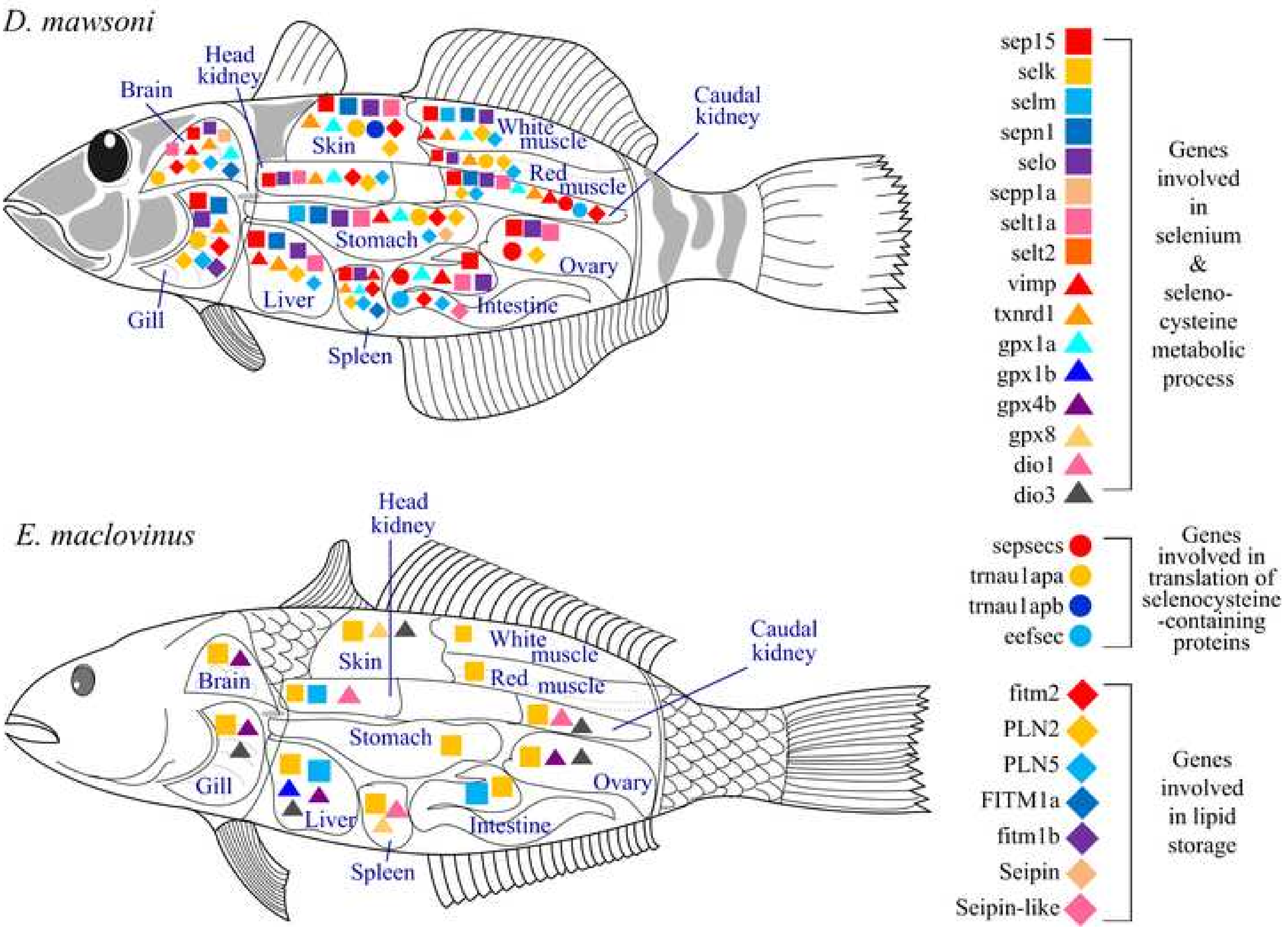

Fig.5

Click here to access/download;Figure;Fig.5.tif

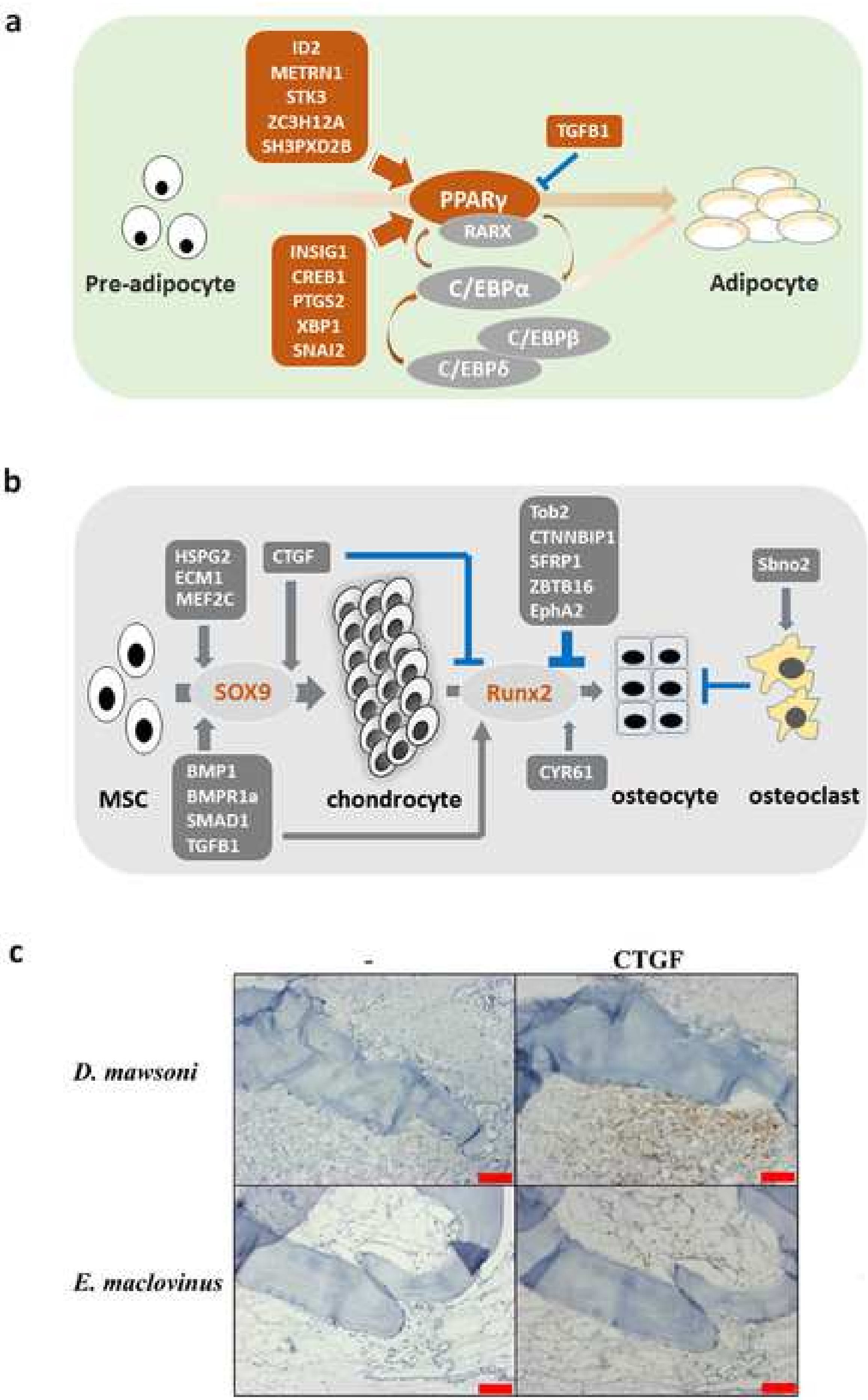

Additional file 1

Click here to access/download

Supplementary Material

Additional file 1 revised26Nov2018.docx

Additional file 2

Click here to access/download
Supplementary Material
Additional file 2.xlsx

Additional file 3

Click here to access/download
Supplementary Material
Additional file 3.xlsx

Additional file 4

Click here to access/download
Supplementary Material
Additional file 4.xlsx

Cover letter Click here to access/download;Personal Cover;CoverLetter26Nov2018.docx

Dear Gigascience editors,

We responded to the reviewers' comments point-to-point and made corresponding revisions to the manuscript entitled "Genomic bases for colonizing the freezing Southern Ocean revealed by the genomes of Antarctic toothfish and Patagonia robalo". The important revisions are outline as below:

1. Paragraph 3 of Introduction: we added a citation of the paper that reports the MHC loci of the Antarctic icefish *C. aceratus* to be more accurately reflecting the status of the genomic studies in the Antarctic fishes.
2. Paragraph 2 of Section "Genome annotation and synteny alignment between *D. mawsoni* and *E. maclovinus*". We added the information of transposable elements of the published *Notothenia coriiceps* genome to comparison, and found that both D. mawsoni and N.coriiceps contained higher TE contents in relative to E. maclovinus. We also corrected two numbers: the TEs content of D. mawsoni (21.38%) and E. maclovinus(10.02%), in this section. The errors were resulted from mistakes made during citing the Additional file 1: Table S9. The statistics of *N. coriiceps* repeats is also added to **Additional file 1: Table S9**.
3. Paragraph 1 of Section "Burst of LINE expansion in the cold": information of LINE expansion in *N. coriiceps* is added. The estimated timing of *N. coriiceps* LINE expansion is also added to **Fig. 2a**.
4. Paragraph 1 of Section " Gene duplication in the freezing environment": the identities of the duplicated genes detected in this study which are consistent with those previously reported in (Chen et al., 2008) were added and shown in **Additional file 1: Fig. S4b**.
5. Section "Specimens, sampling, and DNA and RNA isolation": Sample collection and DNA/RNA preparation are described in more detail.

We believe these revisions have thoroughly addressed the reviewer's concerns. We also edited the manuscript to meet the standard for publication in Gigascience. We look forward to a favorable decision from you.

Sincerely

Liangbiao Chen and Chi-Hing C. Cheng
