# Supplementary material for: The genomic basis for colonizing the freezing Southern Ocean revealed by Antarctic toothfish and Patagonian robalo genomes
Source: Gigascience. 2019 Jan 31;8(4):giz016. doi: 10.1093/gigascience/giz016 (PMC6457430; doi:10.1093/gigascience/giz016)
Supplement: Supplemental Files [file giz016_supplemental_files.zip › Additional file 1 revised26Nov2018.docx]

Additional file 1

***Genomic bases for colonizing the freezing Southern Ocean revealed by the genomes of Antarctic toothfish and Patagonia robalo***

**
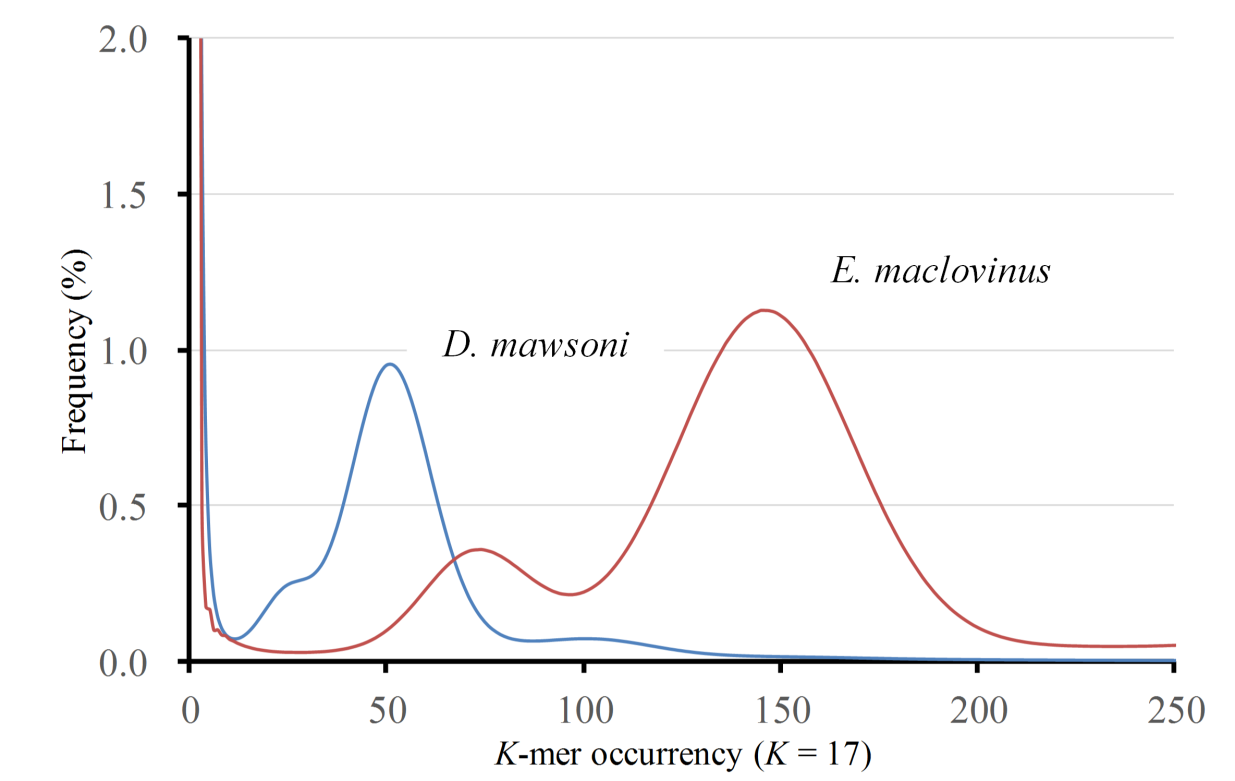
**

**Fig. S1. Distribution of 17-mer frequency**. Values of 17-mers plotted against the frequency at their occurrency. An estimate of genome size (Gs) is the total number of effective *K*-mer words (Kn) divided by the *K*-mer depth or the K-mer occurrence number at the peak *K*-mer frequency (Dp), *i.e.* Gs = Kn /Dp (Li et al., 2009; Murchison et al., 2012). The 17-mer occurrency of the *D. mawsoni* genome peaks at 51. The *D. mawsoni* genome size is thus estimated to be 42,941,246,052 / 51 = 0.842 Gb. Similarly, the *E. maclovinus* genome size is estimated to 104,744,458,034 / 144 = 0.727 Gb.

**Statistical information 17-mer frequency for Figure S1.**

|  | ***K*-mer_num** | **Peak depth** | **Genome_size** | **Used_base** | **Used_read** | **Seq. depth** |
| --- | --- | --- | --- | --- | --- | --- |
| *D. maw* | 42,941,246,052 | 51 | 0.842 GB | 50,537,028,100 | 474,736,378 | 60.02X |
| *E. mac* | 104,744,458,034 | 144 | 0.727 GB | 125,153,450,626 | 1,275,562,037 | 172.15x |

**
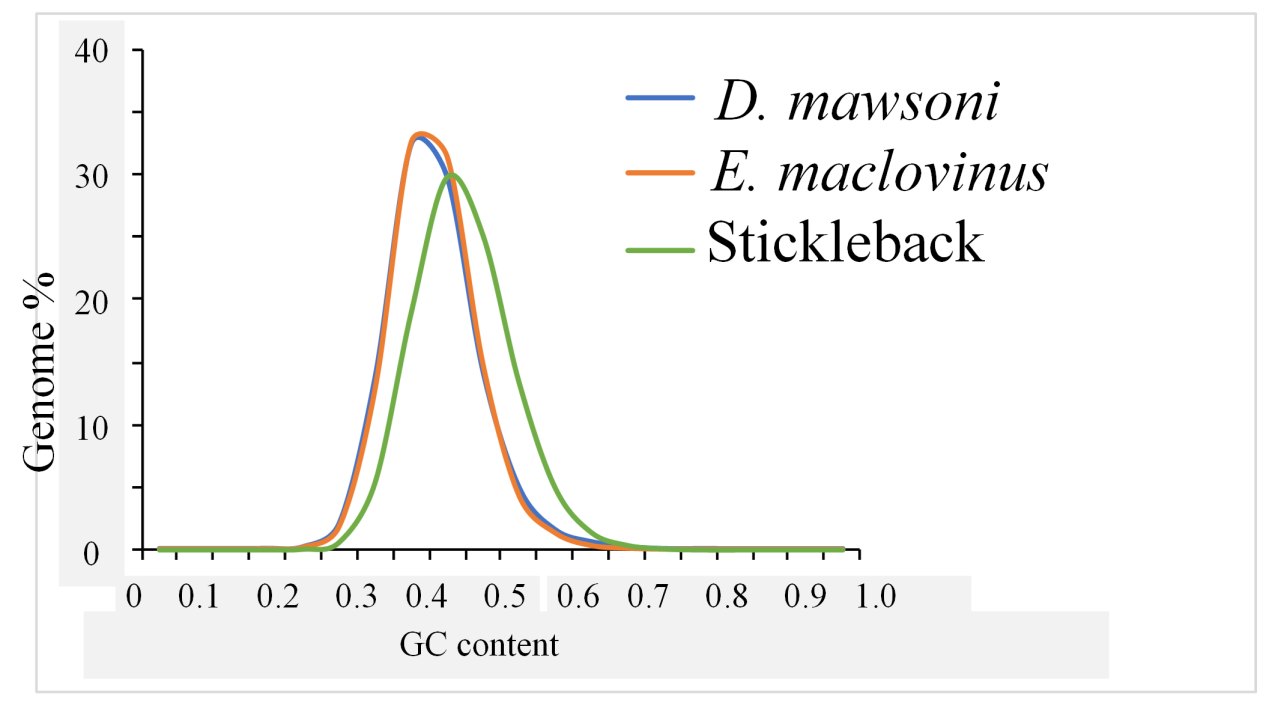
**

**Fig. S2.** **Comparison of GC content of *D. mawsoniE.*** *maclovinus* and stickleback (*Gasterosteus aculeatus*). The two notothenioids share similar GC content, which is lower than the temperate stickleback.


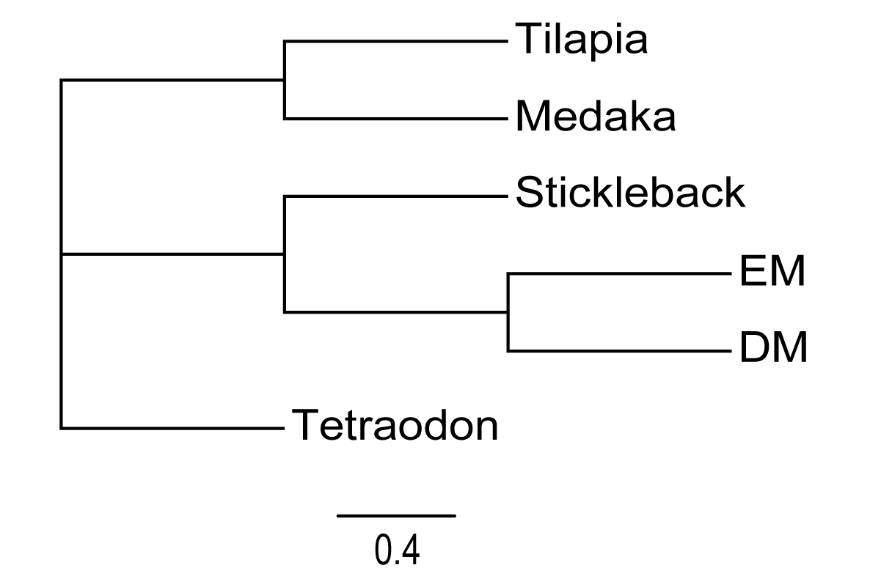


E. maclovinus

D. mawsoni


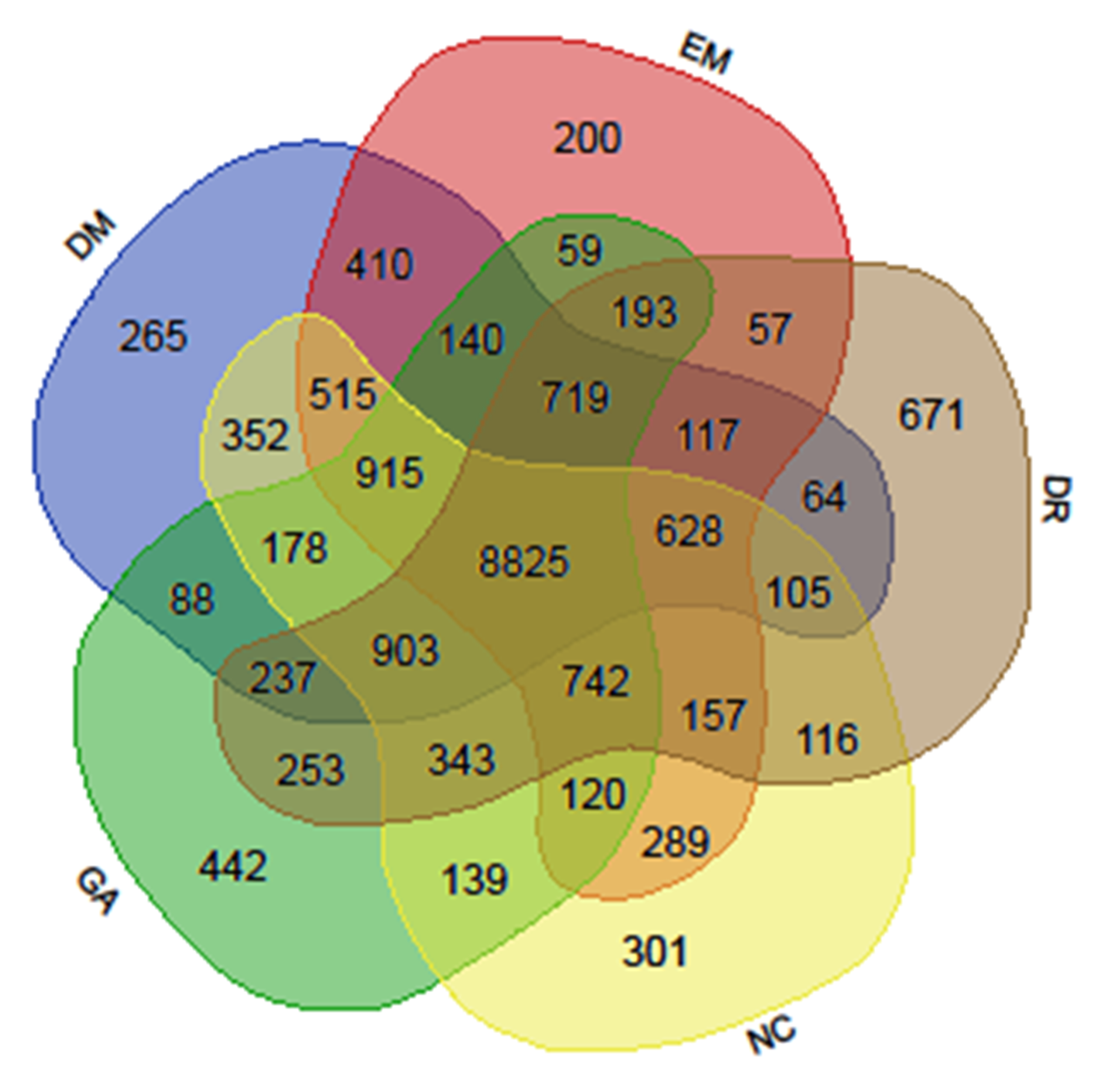


**Fig. S3. Venn diagram of gene clusters for five selected fish genomes.** Each number represents the number of orthologous gene families shared by the indicated genomes. DM - *D. mawsoni*; NC - *N. coriiceps*; EM - *E. maclovinus*; GA - *G. aculeatus*; and DR - zebrafish *Danio rerio*.

**4a**

**4b**

**
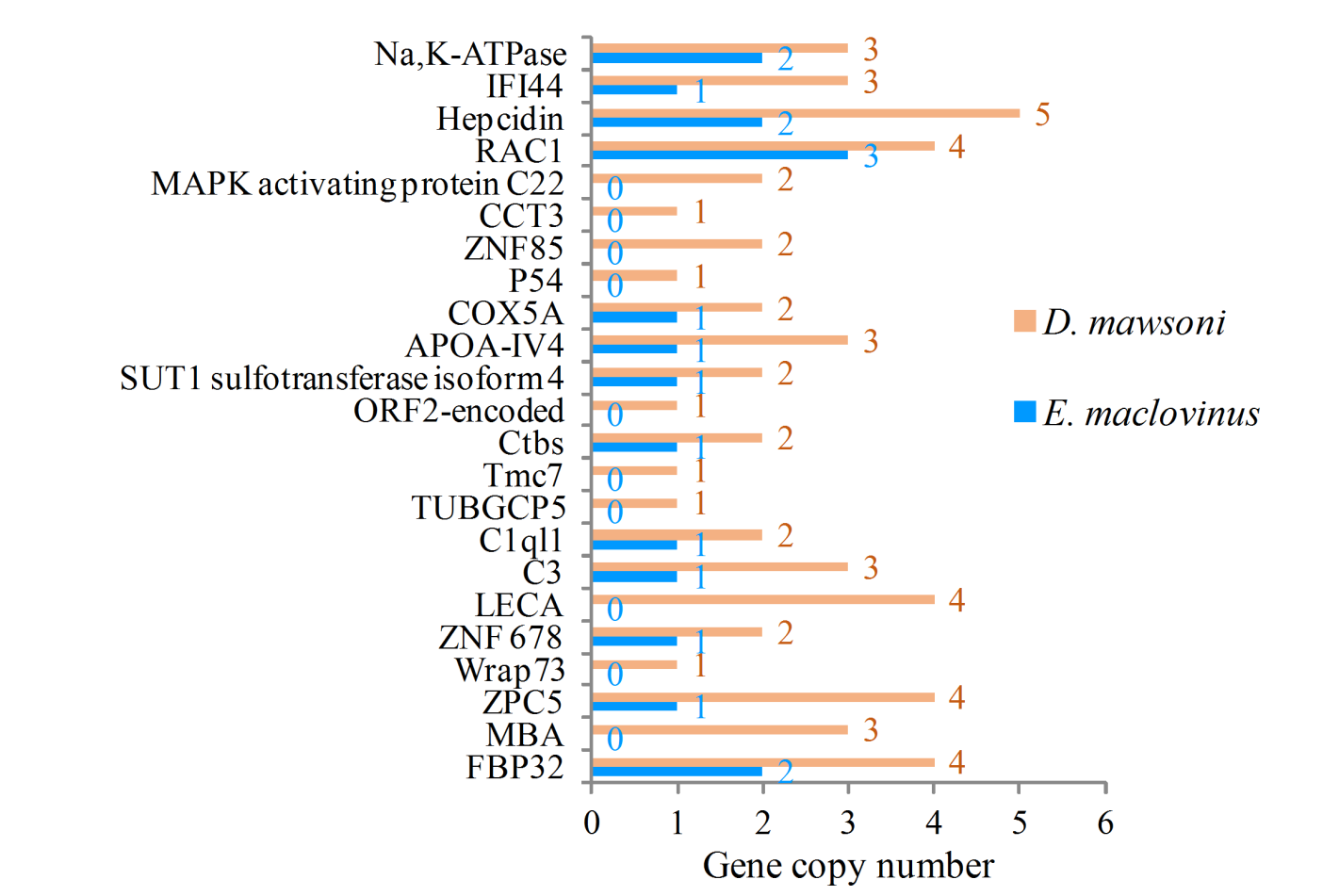
**

**Fig. S4. Gene evolution. (a)** Phylogenetic tree used in testing for genes under positive selection in Antarctic toothfish *D. mawsoni* using branch-site model implemented in PAML (Yang, 2007). The tree is derived by using the supergene containing the homologous protein coding genes between the six species. (**b**) Genes that have been duplicated in *D. mawsoni*. The copy numbers of the *D. mawsoni* and *E. maclovinus* genes are indicated in orange and blue numbers, respectively. (The antifreeze glycoprotein gene that evolved *de novo* and had extensively duplicated are not included in the list).

**5a**

LcZPC5a      METVYFWVNLLVGLLLSGFCVRSSLAFPPKHYTQHALLQRPQLTRRTVQE

LcZPC5b      METVYFWVNLLVGLLLSGFCVRSSLAFPPKHYTQHALLQRPQLTRRTVQE

DmZPC5_2b    -----MEAFNFQVILLVGLCVSSSFAFPPTRYTQDASFQSLANTGRSEIG

DmZPC5_2a    -----MEAFNFQVILLVGLCVSSSFAFPPTRYTQDASFQSLANTGRSEIV

DmZPC5_1     -----MEAFNFQVILLVGLCVSSSFAFPPSRYTQDASFQSLANTSRSEIV

DmZPC5_3     -----MEAFNFQVILLVGLCVSSSFAFPPTPYTQDASFQSLANTSRSEIV

EmZPC5       -----MEAFYFQVVLLIGLCVSSSFAFPPTRYTQDASFQSPAITGRSKIS

TrZPC5       --------------------------------------------------

OlZPC5       -MAPSRLKISCLFGHLTAFCLQLTLAFPPLHYVPPVSLKSQSSLPSVVQQ

LcZPC5a      EQ---------KAPAEGREQVNTVGVTCHPDSLEIVIKADMFGVGAPVNA

LcZPC5b      QQ---------KAPAEGREQVNTVGVTCHPDSLEIVIKADMFGVGAPVNA

DmZPC5_2b    QQQQQQQQQQQKSPAEEPQQVNTIRVTCHPDSLEIVIKADMFAVGAPVDA

DmZPC5_2a    QQ------QQQKSPAEEPQQVNTIRVTCHPDSLEIVIKADMFAVGAPVDA

DmZPC5_1     QQ--------QKSKAEEPQQVNTIRVTCHPDSLEIVIKADMFAVGAPVDA

DmZPC5_3     QQ---------KSKAEEPQQVNTIRVTCHPDSLEIVIKADMFAVGAPVDA

EmZPC5       QQ--------QKFPAEERPQVNTIRVTCHPDSLEIVIKADMFAVGAPVNG

TrZPC5       --------------------VKTVRVNCHPNSLEIVVKADMFEIGAPVYS

OlZPC5       PE--------------EPVPVNTVGVLCHPDSMELSINADLFEVGAPVDV

                                 *:*: * ***:*:*: ::**:* :****

LcZPC5a      DELRLGVEHG-DFCRATSSSG---DEYRIIAGLLDCGTKHWMTQDSLVYT

LcZPC5b      DELRLGVEHS-DFCRATSSLG---DEYRIIAGLLDCGTKHWMTQDSLVYT

DmZPC5_2b    DEIRLGVETNNQYCRATASSA---DEYSISVGLVECGTRHWVTEDSLIYT

DmZPC5_2a    DEIRLGVETNNQYCRATASSA---DEYSISVGLVECGTRHWVTEDSLIYT

DmZPC5_1     DEIRLGVETNNQYCRATASSA---DEYSISVGLVECGTRHWVTEDSLIYT

DmZPC5_3     DEIRLGVETNNQYCRATASSA---DEYSISVGLVECGTRHWVTEDSLIYT

EmZPC5       DEIRLGVEYN-DYCRATASSG---DEYSIIVGLMQCGTRHWVTEDSLIYT

TrZPC5       DELRLGVEQR-DHCRAHASSEGGEEEYTILVGLADCGTKHWVSEDALIYT

OlZPC5       RELRLGVEHS-DYCSATASSD---SEYRILVGLEDCGTKHWMTEDSLVYT

              *:*****   :.* * :*     .** * .** :***:**:::*:*:**

LcZPC5a      NLLIYSPVASPDGVIRMDEAVIPIECHYERKYSLSSSPIAPTWIPFMSTQ

LcZPC5b      NLLIYSPVASPDGVIRMDEAVIPIECHYERKYSLSSSSIAPTWIPFMSTQ

DmZPC5_2b    NLLIYSPEASPYGVVRMDEAVIPIECHYERKYSVSSSSLMPTWIPFMSTQ

DmZPC5_2a    NLLIYSPEASPYGVVRMDEAVIPIECHYERKYSVSSSSLMPTWIPFMSTQ

DmZPC5_1     NLLIYSPEASPYGVVRMDEAVIPIECHYERKYSVSSSSLMPTWIPFMSTQ

DmZPC5_3     NLLIYSPEASPYGVVRMDEAVIPIECHYERKYSVSSSSLMPTWIPFMSTQ

EmZPC5       NLLIYSPEPSPYGVVRMDEAVIPIECHYQRKYSLSSSSLTPTWIPFKSTQ

TrZPC5       NLLIFSPRVTPDGLIRMDEAVIPIECQYERKYSLSSSSLTPTWVPFMATQ

OlZPC5       NLLIYTPLPALNGITRMEEAVIPIECQYKRKYSLSSSSLVPTWVPFTSTQ

             ****::*  :  *: **:********:*:****:***.: ***:** :**

LcZPC5a      AAVETLEFDLRIMTD-------------------------DWLYERSSNV

LcZPC5b      AAVETLEFDLRIMTD-------------------------DWLYERSSNV

DmZPC5_2b    AAVEMLQFNLRIMTS-------------------------DWQYKRSSNV

DmZPC5_2a    AAVEMLQFNLRIMTS-------------------------DWQYKRSSNV

DmZPC5_1     AAVEMLQFNLRIMTS-------------------------DWQYKRSSNV

DmZPC5_3     AAVEMLQFNLRIMTS-------------------------DWQYKRSSNV

EmZPC5       AAVEMLEFNLRIMTGRFFIFSCCVILSLFTLSTLFYLCPGDWQYKRSSNV

TrZPC5       AAVETLAFDLRLVTD-------------------------DWLYDRRSHV

OlZPC5       AAVETLQFNLRLMTN-------------------------DWLHERGANT

             **** * *:**::*.                         ** :.* ::.

LcZPC5a      FYLGEPISIEASLRIGHHMGLRVFMNSCVATLYPDIYSVPRYVFIENGCL

LcZPC5b      FYLGEPISIEASLRIGHHMGLRVFMNSCVATLYPDTYSVPRYVFIENGCL

DmZPC5_2b    FHLGEPISIEASVRIGHHMGLRVFVSSCVATLSPDMNSSPRHAFIENGCF

DmZPC5_2a    FHLGEPISIEASVRIGHHMGLRVFVSSCVATLSPDMNSSPRHAFIENGCF

DmZPC5_1     FDLGEPISIEASVRIGHHMGLRVFVSSCVATLSPDMNSSPRHAFIENGCF

DmZPC5_3     FHLGEPISIEASVRIGHHMGLRVFVSSCVATLSPDMNSSPRHAFIENGCF

EmZPC5       FHLGEPISIEASVRIGHHMGLRVFVSSCVATLNPDMHSVPRHAFIENGCL

TrZPC5       FHLGDLIPVEASVHVGHHLGLRVFVNNCVATLSPDVLSQPRHVFIENGCF

OlZPC5       FFLGEPINIEASVRVDHHMGLRLFLNSCVATLEPNIKSDPKYVFIENGCL

             * **: * :***:::.**:***:*:..***** *:  * *::.******:

LcZPC5a      VDSHIPGSRSHFLSRTQDDKLHLVIDAFRFYNEDRGQLYITCHLNAVPVN

LcZPC5b      VDSHIPGSRSHFLSRTQDDKLQLVIDAFRFHNEDRGQLYITCHLNAVPVN

DmZPC5_2b    VDSQLPGSRSQFLARTQDDKLHMSIDAFRFYNEDRGELYVTCHLHAEPIN

DmZPC5_2a    VDSQLPGSRSQFLARTQDDKLHMSIDAFRFYNEDRGELYVTCHLHAEPIN

DmZPC5_1     VDSQLPGSRSQFLARTQDDKLHMSIDAFRFYNEDRGELYITCHLNAEPIN

DmZPC5_3     FDSQLPGSRSQFLARTQDDKLHMSIDAFRFYNEDRGELYITCHLHAVPIN

EmZPC5       VDSQLPGSRSHFLTRTQDDKLHISIDAFRFYNEDRGELYITCHLNAVPIN

TrZPC5       IDSQLPGSKSQFLPRTQDEKLRVLIDAFRFHNQDGGELYITCQLNAVPAN

OlZPC5       LDSQLPGSKGHFLPRTKDNILQMTIDSFKFHNDERGQLYITCHLNAVPVD

             .**::***:.:**.**:*: *:: **:*:*:*:: *:**:**:*:* * :

LcZPC5a      NAEAPSKACTFLNGRWR----------SADGNDYLCGHCQSQNEIDQTRS

LcZPC5b      NAEAPSKACTFLNGRWR----------SADGNDYLCGHCQSQNEIDQTRS

DmZPC5_2b    DTVATNKACTFVNGRWR----------SADGNDYLCGQCKRPIEVEQTPS

DmZPC5_2a    DTVATNKACTFVNGRWR----------SADGNDYLCGQCKRPIEVEQTPS

DmZPC5_1     DADAT-------NKAWR----------SADGNDYLCGQCKRPIEVEQTPS

DmZPC5_3     DTVATDKACTFVNGRWR----------SADGNDYLCGQCKRSIEVAQTP-

EmZPC5       DAEAPNKACTFVNGRQVRSNAKYVKWRSADGNDYLCGHCKRQNEIAQTPS

TrZPC5       DPEAPQKACTFVNGRWK----------SANSNDYLCASCQSHG--KPGHG

OlZPC5       DAEAQSKACTYVNGRSIED-------------------------------

             :. *        *

LcZPC5a      KPSKTDQFSPRGFGKPDKPETFWRSGLETNKVWEQEATVGPMMVLPAMKK

LcZPC5b      KPSKTDQFSPRGFGKPDKPETFWRSGLETNKVWEQEATVGPMVVLPAMKK

DmZPC5_2b    KPSSPSKFRPRGFVKPEEREPLWRSGLKTSTVWEHQARVGPLMVLPTKQK

DmZPC5_2a    KPSSPSKFRPRGFVKPEEREPLWRSGLKTSTVWEHQARVGPLMVLPTKQK

DmZPC5_1     KPSSPSKFRPRGFVKPEEREPLWRSGLKTSTVWEHQARVGPLMVLPAKQK

DmZPC5_3     --SSPSKFRPRGFVKPEEREPLWRSGLKTSTVCEHQARVGPLMVLPAKQK

EmZPC5       KPSSPSKYGPRGFVKPEEREPLWRSGLKTNTVWEQDARVGPMMVLPAKQK

TrZPC5       KPSAPGKFGPRGFQKPESK---WRSGTLTKPVLEQEARMGPLVVLPAPR-

OlZPC5       FFFKFTFFFXXXXGKFADMEPMWRSGLKTNKVWEHEARLGPVTILPSWKS

                    :      *  .    ****  *. * *::* :**: :**: :

LcZPC5a      SGPIPDEELPPVLKKISR-PLYGSKWRS---GVNEKSVDLEKGLLP-GPS

LcZPC5b      SGPIPDEELPPVLKKISR-PLYGSQWRS---GVNEKSVDLEKGLLP-GPS

DmZPC5_2b    SRPIPAEERSSILDKISRSTMYGSQWRS---GIN--RVDQRKGLLPDSSS

DmZPC5_2a    SRPIPAEERSSILDKISRSTMYGSQWRS---GIN--RVDQRKGLLPDSSS

DmZPC5_1     SRPIPAKERSSILDKISRSTMYGSQWRS---GIN--RVDQRKGLLPDSSS

DmZPC5_3     SRPIPAEERSSILDQISRFPMYGSQWRS---GIN--GVDQRKGLLPDSLS

EmZPC5       SRPIPVEALPSTLNKLSRSTMYGSQWRS---GIN--RVDPRKGLLPDSSS

TrZPC5       -SPLPAQELPPVLSNILKPRLYGSQWRSGPR-------AEPKALVPDTPD

OlZPC5       -GALAAHELPPVLHKIHKTALYGSHWRSG-------LNTIEKSLVPEPSS

               .:. .  .. * :: :  :***:***             *.*:*   .

LcZPC5a      APDQVEVQTVAPEENKDVKSGTDLTDGDAESEVEAPLEESAVTLKSKDVA

LcZPC5b      APDQVEVQTVAPEENKDVKSGTDLTDGDAESEVEAPLEESAVSLKSKDVT

DmZPC5_2b    TQNQVDILTLASEQNKDREDKNGTEKDEDAEEVHELLEKNLS--------

DmZPC5_2a    TQNQVDILTLASEQNKDREDKNGTEKDEDAEEVHELLEKNLS--------

DmZPC5_1     TQNQVDVLTLASEENKD---KNGTEKDEDAEEVHELLEKTSPEAHLQSKA

DmZPC5_3     TQNQVDVLTLASEQNKD---------------------------------

EmZPC5       TQHQVAVPTLASENKEEEEKSGMDE--EDAEEVPELLEKASPETHLQSKA

TrZPC5       PDQEETSEDASGEELKSEGSEGAEIPAVSGAPLRSVAEQQTSAMSSSRRA

OlZPC5       SELEEDDSDDYDESDSDLAFVMKSLEIAHTNTTFPIVTYDLEPSKMNATA

             .  :        *. ..

LcZPC5a      VLDTNGTSALSDVGPAAQLDVTTLSNANATDLSHTDDPKR------

LcZPC5b      VLNTNGTSALSDVGPSAQLDVTTLSNANATDLSHTDDPKR------

DmZPC5_2b    ----------------------------------------------

DmZPC5_2a    ----------------------------------------------

DmZPC5_1     AVLNGTDKAALDEVFPTAAVNVAVPPLSNTTATVSDLSETMDPKRK

DmZPC5_3     ----------------------------------------------

EmZPC5       AVLGSNDTATLEVLPATPVTVDVTSLSNTTSTDSKDPKR-------

TrZPC5       PTA-------------------------------------------

OlZPC5       ANPDLSDKHDPKK---------------------------------

**5b**


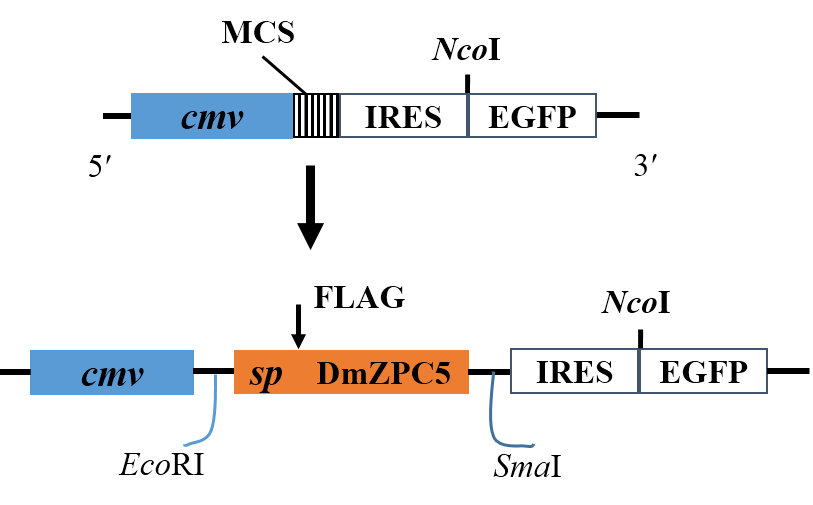


**Fig. S5. Cloning of DmZPC5 into the expression vector pIRES2-EGFP.** **(a)** Amino acid sequence alignment of three DmZPC5 size isoforms from *D. mawsoni*, and ZPC5 orthologs from *E. maclovinus* (Em), *Larimichthys crocea* (Lc), *Tetraodon rubripes* (Tr), and Oryzias latipes (Ol). Asterisks and colons indicated the conserved and partially conserved amino acids, respectively. Accession numbers are XP_019117429.1 (LcZPC5a), XP_019120575.1 (LcZPC5b), ENSTNIG00000005275.1 (TrZPC5), ENSORLG00000009853.1 (OlZPC5), and sequence IDs from our database evm.model.scaffold555.4 (EmZPC5), Dissostichus_mawsoni_GLEAN_1051387 (DmZPC5_1), Dissostichus_mawsoni_ GLEAN_10018489 (DmZPC5_2a), Dissostichus_mawsoni_GLEAN_10015797 (DmZPC5_2b), Dissostichus_mawsoni_GLEAN_10017685 (DmZPC5_3). **(b)** Vector information for DmZPC5 expression. The FLAG tag sequence (FLAG) was incorporated into the DmZPC5 cDNA sequence immediately downstream from its native signal peptide (*sp*) and cloned into the *Eco*RI and *Sma*I restriction sites in the multiple cloning sites (MCS). The expression is driven by the *cmv* promoter. EGFP is translated in parallel under the control of IRES to facilitate the measuring of transfection efficiency.

6

**Fig. S6. Western blot analysis for the location of DmZPC5 isoform products from transfected CHO cells.** Proteins were separated on a 10% reducing/denaturing SDS-PAGE gel. **(A)** The distribution of the DmZPC5 products as secreted form (labelled “SQ”) or maintained inside the cell (labelled “cell”). While DmZPC5_1 has product located both inside the cell and in secreted form, DmZPC5_2a and DmZPC5_3 were hardly detected in secretion. All the lanes are loaded with product extracted from approximately same amount of transfected cells (for cell) or same volume of the culture medium (for SQ). **(B)** The time course of DmZPC5 isoform secretion from 1 to 3 days. DmZPC5_1 demonstrated the highest secretory activity, while DmZPC5_2a were less activity, and DmZPC5_3 did not secret during the entire test time period.

**7a**

**7b**


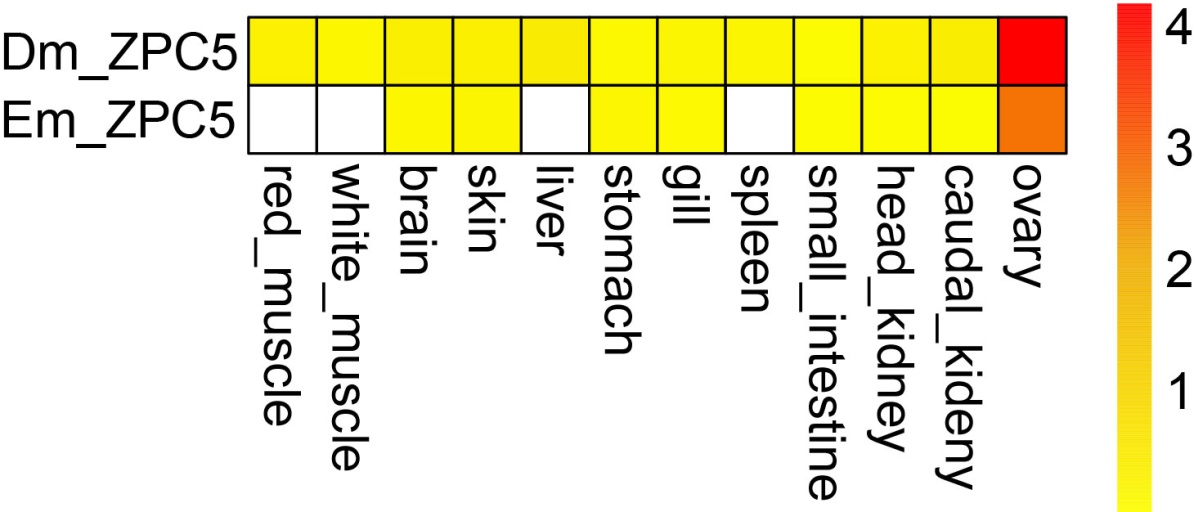


**Fig. S7.** (**a**) Western blot analysis for the DmZPC5 isoforms expressed in CHO cells at different temperatures 0℃ and 37℃ on native PAGE gel. The proteins were separated on native PAGE gel, namely, neither SDS and nor other reducing agents such as DTT were used in the gel and loading buffer, such that the proteins were presumably maintained at the native state (see reference Cao et al., 2016 in the main text for detail). Results indicated that DmZPC5_1 was more likely present as polymers than DmZPC5_2a and DmZPC5_3. **(b)** Qualified expression levels of ZPC5 genes across various tissues in *D. mwasoni* (Dm_ZPC5) and *E. maclovinus* (Em_ZPC5). Expression levels of each gene (log2(FPKM)) is shown by color.

**8a**


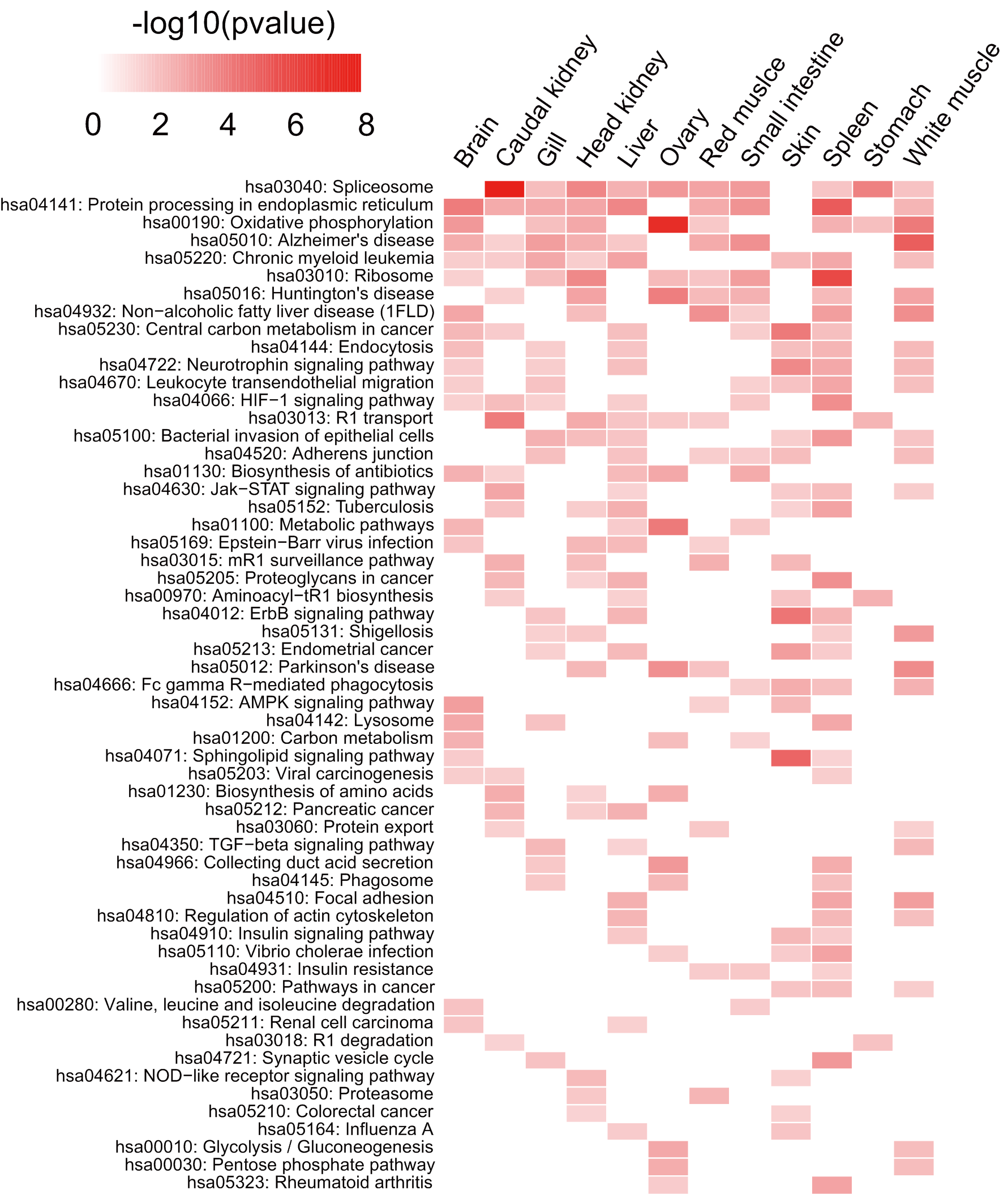


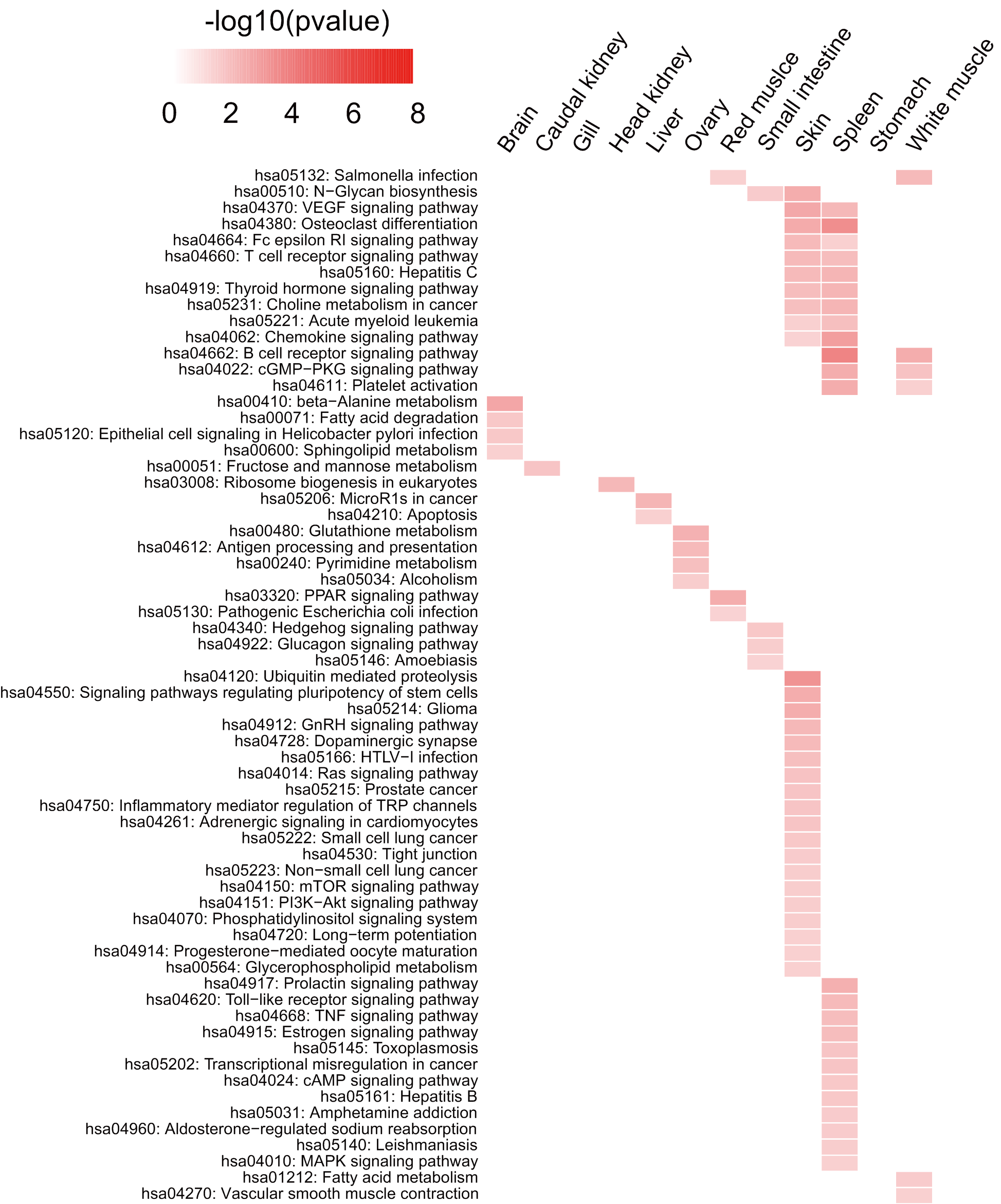


**8b**


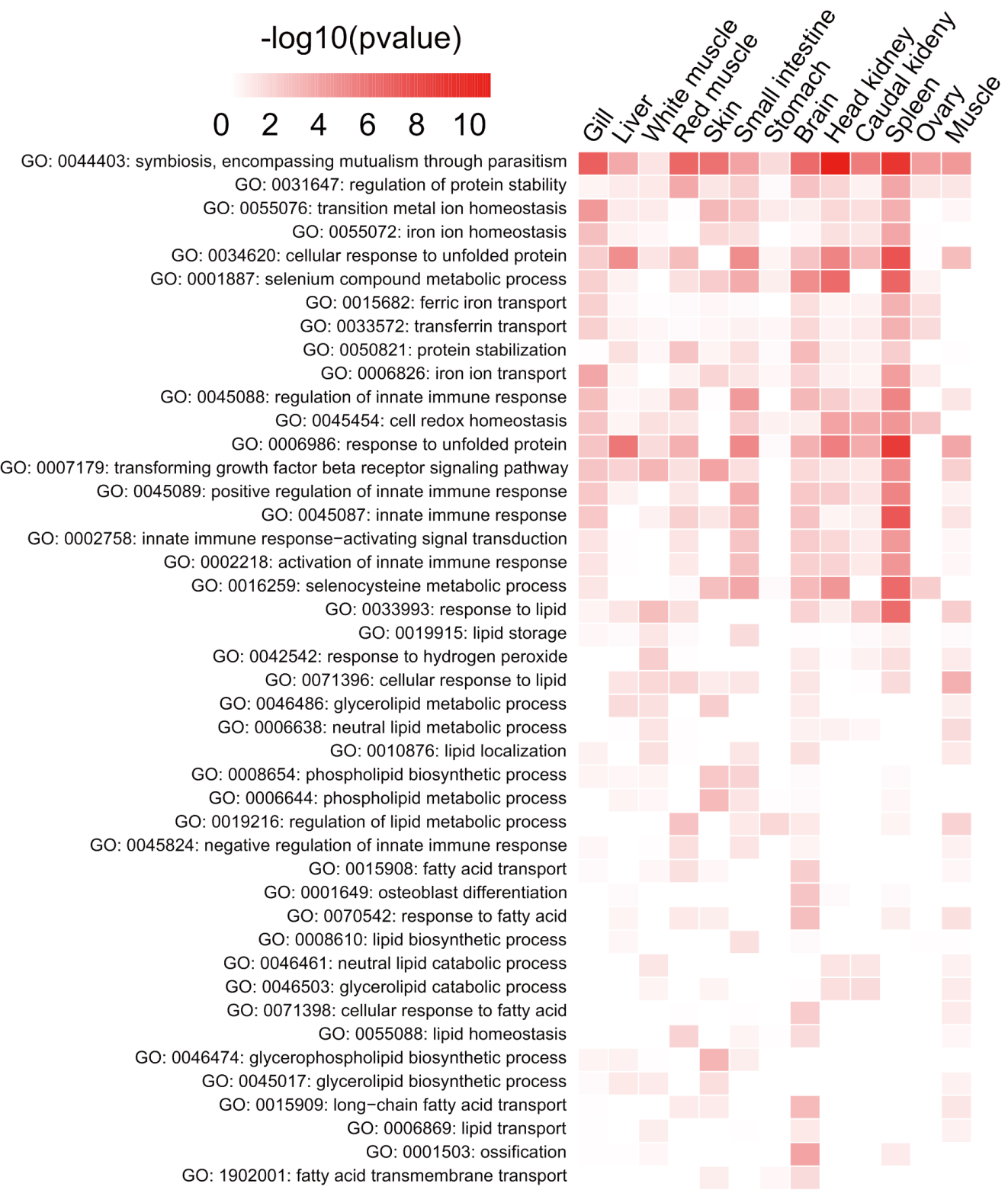


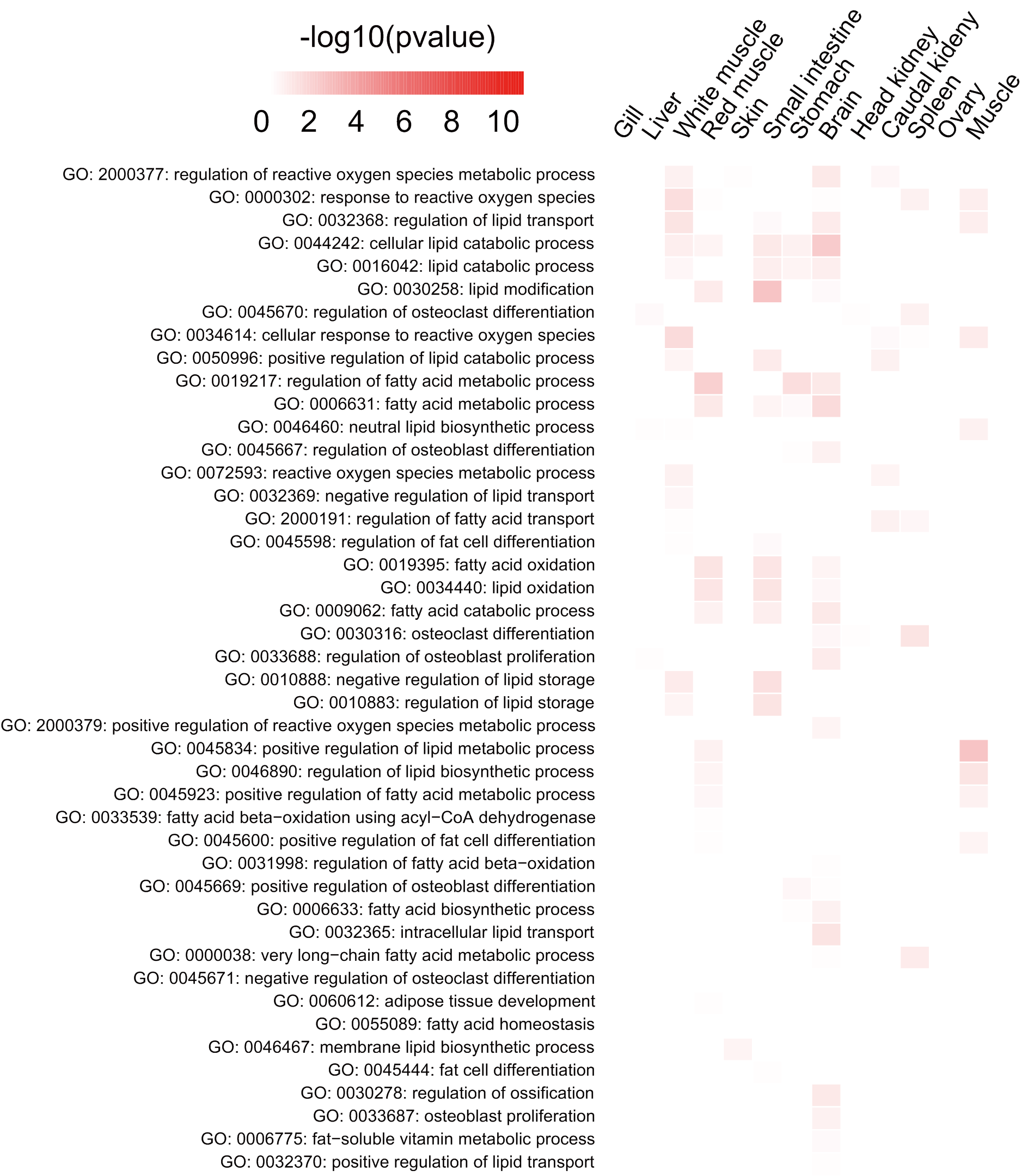


**Fig. S8. Enrichment KEGG pathways and GO terms across *D. mawsoni* tissues compared to *E. maclovinus*. (a)** Significance of enriched KEGG pathways in *D. mawsoni* genes with significantly higher expression levels than *E. maclovinus*. The *p* values of the enrichment are scaled to the intensity of the red color. **(b)** GO term enrichment (*p* value) of the *D. mawsoni* DEGs involved in biological process.


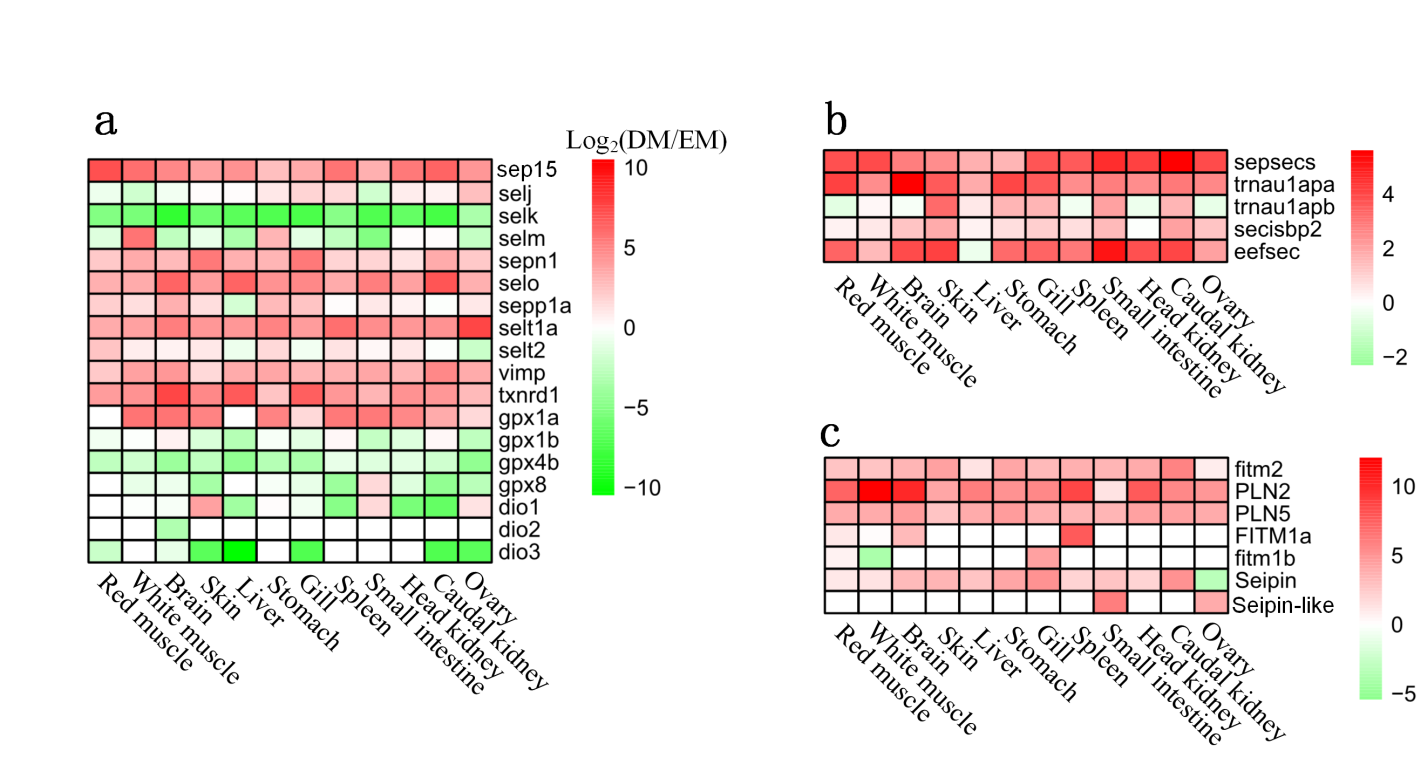


**Fig. S9. Comparison of gene expression between *D. mawsoni* and *E. maclovinus* tissues.** Gene expression was measured by quantified transcription levels (FPKM, fragments per kilobase of transcript per million mapped reads) derived from transcriptome analyses. Differential gene expressions in the selected 12 tissues are indicated by the fold change (Log_2_(*D. mawsoni* / *E. maclovinus*)) shown by the color scale. (**a**) The genes involved in selenium compound metabolic process and selenocysteine metabolic process. (**b**) The genes involved in translation of selenocysteine-containing proteins. (**c**) The genes involved in lipid storage.

(The detailed FPKM values are listed in the suppelementary Dataset S1).


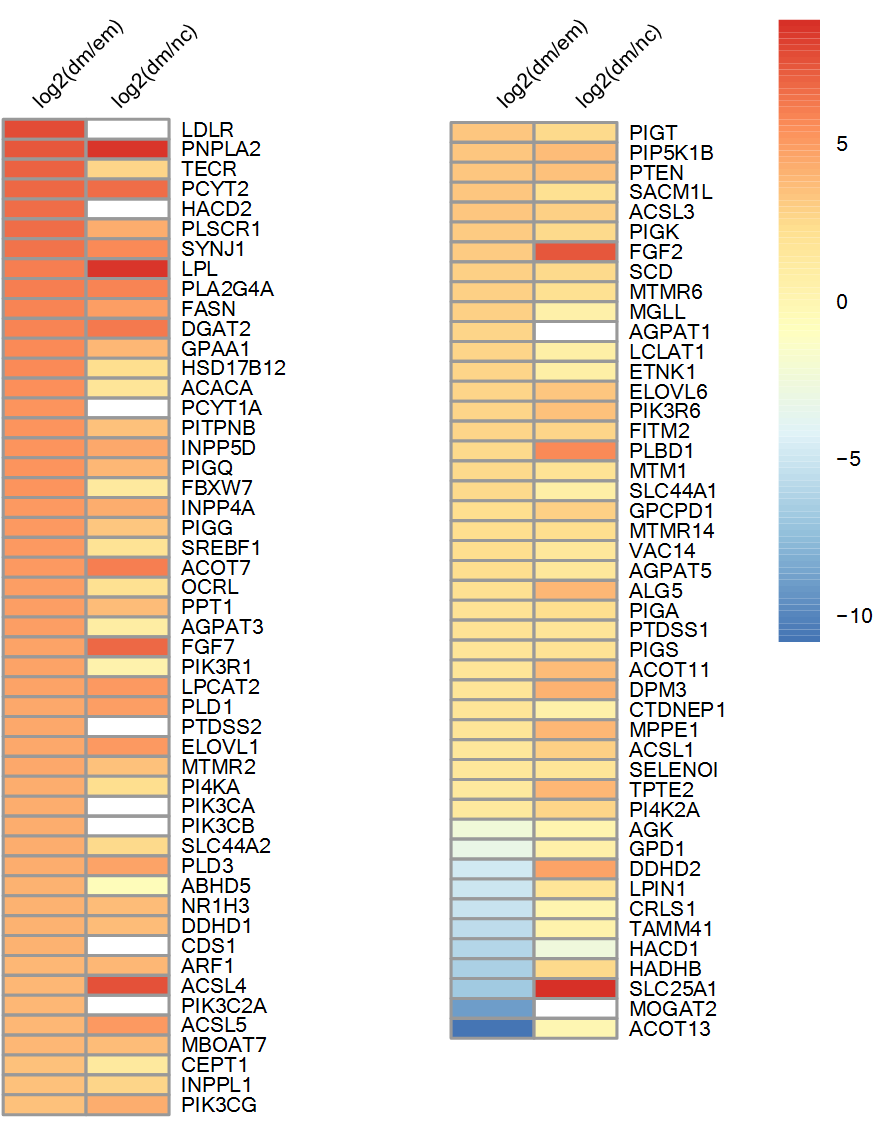


**Fig. S10. Comparison of DEGs (Differentially Expressed Genes) involved in glycerolipid biosynthesis in *D. mawsoni* versus two other notothenioids.** Gene expression levels (FPKM) were compared between *D. mawsoni* and *E. maclovinus* (log2(dm/em)), and between *D. mawsoni* and *N. coriiceps* (log2(dm/nc)), according to the color scale

Fig. S11


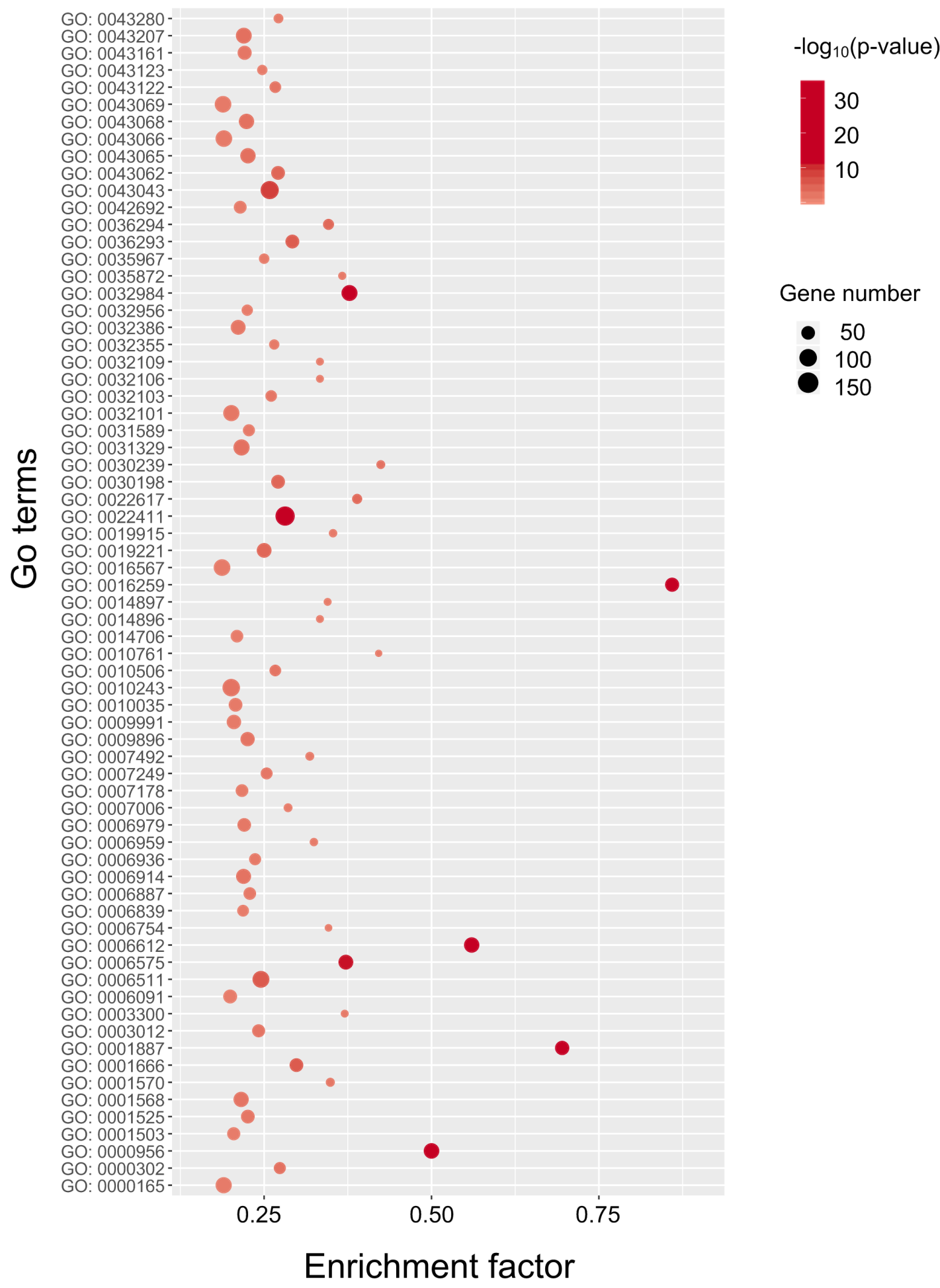


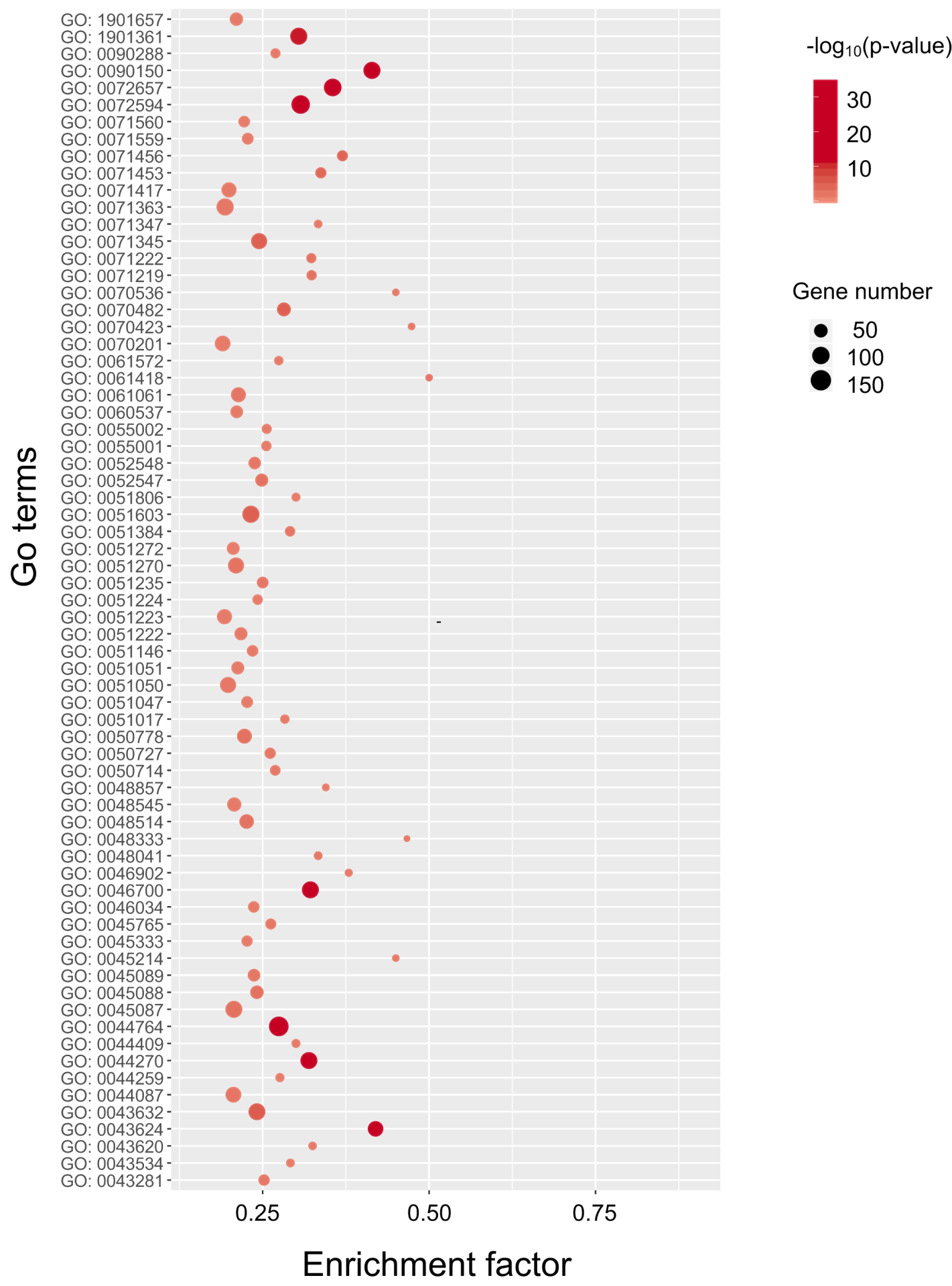


**Fig. S11. GO term enrichment of the *D. mawsoni* bone DEGs.** P values and gene numbers are scaled to the color intensity and size of the filled circles, respectively. The description of each GO terms are listed as follows:

| **GO** | **Description** |
| --- | --- |
| GO: 1901657 | glycosyl compound metabolic process |
| GO: 1901361 | organic cyclic compound catabolic process |
| GO: 0090288 | negative regulation of cellular response to growth factor stimulus |
| GO: 0090150 | establishment of protein localization to membrane |
| GO: 0072657 | protein localization to membrane |
| GO: 0072594 | establishment of protein localization to organelle |
| GO: 0071560 | cellular response to transforming growth factor beta stimulus |
| GO: 0071559 | response to transforming growth factor beta |
| GO: 0071456 | cellular response to hypoxia |
| GO: 0071453 | cellular response to oxygen levels |
| GO: 0071417 | cellular response to organonitrogen compound |
| GO: 0071363 | cellular response to growth factor stimulus |
| GO: 0071347 | cellular response to interleukin-1 |
| GO: 0071345 | cellular response to cytokine stimulus |
| GO: 0071222 | cellular response to lipopolysaccharide |
| GO: 0071219 | cellular response to molecule of bacterial origin |
| GO: 0070536 | protein K63-linked deubiquitination |
| GO: 0070482 | response to oxygen levels |
| GO: 0070423 | nucleotide-binding oligomerization domain containing signaling pathway |
| GO: 0070201 | regulation of establishment of protein localization |
| GO: 0061572 | actin filament bundle organization |
| GO: 0061418 | regulation of transcription from RNA polymerase II promoter in response to hypoxia |
| GO: 0061061 | muscle structure development |
| GO: 0060537 | muscle tissue development |
| GO: 0055002 | striated muscle cell development |
| GO: 0055001 | muscle cell development |
| GO: 0052548 | regulation of endopeptidase activity |
| GO: 0052547 | regulation of peptidase activity |
| GO: 0051806 | entry into cell of other organism involved in symbiotic interaction |
| GO: 0051603 | proteolysis involved in cellular protein catabolic process |
| GO: 0051384 | response to glucocorticoid |
| GO: 0051272 | positive regulation of cellular component movement |
| GO: 0051270 | regulation of cellular component movement |
| GO: 0051235 | maintenance of location |
| GO: 0051224 | negative regulation of protein transport |
| GO: 0051223 | regulation of protein transport |
| GO: 0051222 | positive regulation of protein transport |
| GO: 0051146 | striated muscle cell differentiation |
| GO: 0051051 | negative regulation of transport |
| GO: 0051050 | positive regulation of transport |
| GO: 0051047 | positive regulation of secretion |
| GO: 0051017 | actin filament bundle assembly |
| GO: 0050778 | positive regulation of immune response |
| GO: 0050727 | regulation of inflammatory response |
| GO: 0050714 | positive regulation of protein secretion |
| GO: 0048857 | neural nucleus development |
| GO: 0048545 | response to steroid hormone |
| GO: 0048514 | blood vessel morphogenesis |
| GO: 0048333 | mesodermal cell differentiation |
| GO: 0048041 | focal adhesion assembly |
| GO: 0046902 | regulation of mitochondrial membrane permeability |
| GO: 0046700 | heterocycle catabolic process |
| GO: 0046034 | ATP metabolic process |
| GO: 0045765 | regulation of angiogenesis |
| GO: 0045333 | cellular respiration |
| GO: 0045214 | sarcomere organization |
| GO: 0045089 | positive regulation of innate immune response |
| GO: 0045088 | regulation of innate immune response |
| GO: 0045087 | innate immune response |
| GO: 0044764 | multi-organism cellular process |
| GO: 0044409 | entry into host |
| GO: 0044270 | cellular nitrogen compound catabolic process |
| GO: 0044259 | multicellular organismal macromolecule metabolic process |
| GO: 0044087 | regulation of cellular component biogenesis |
| GO: 0043632 | modification-dependent macromolecule catabolic process |
| GO: 0043624 | cellular protein complex disassembly |
| GO: 0043620 | regulation of DNA-templated transcription in response to stress |
| GO: 0043534 | blood vessel endothelial cell migration |
| GO: 0043281 | regulation of cysteine-type endopeptidase activity involved in apoptotic process |
| GO: 0043280 | positive regulation of cysteine-type endopeptidase activity involved in apoptotic process |
| GO: 0043207 | response to external biotic stimulus |
| GO: 0043161 | proteasome-mediated ubiquitin-dependent protein catabolic process |
| GO: 0043123 | positive regulation of I-kappaB kinase/NF-kappaB signaling |
| GO: 0043122 | regulation of I-kappaB kinase/NF-kappaB signaling |
| GO: 0043069 | negative regulation of programmed cell death |
| GO: 0043068 | positive regulation of programmed cell death |
| GO: 0043066 | negative regulation of apoptotic process |
| GO: 0043065 | positive regulation of apoptotic process |
| GO: 0043062 | extracellular structure organization |
| GO: 0043043 | peptide biosynthetic process |
| GO: 0042692 | muscle cell differentiation |
| GO: 0036294 | cellular response to decreased oxygen levels |
| GO: 0036293 | response to decreased oxygen levels |
| GO: 0035967 | cellular response to topologically incorrect protein |
| GO: 0035872 | nucleotide-binding domain, leucine rich repeat containing receptor signaling pathway |
| GO: 0032984 | macromolecular complex disassembly |
| GO: 0032956 | regulation of actin cytoskeleton organization |
| GO: 0032386 | regulation of intracellular transport |
| GO: 0032355 | response to estradiol |
| GO: 0032109 | positive regulation of response to nutrient levels |
| GO: 0032106 | positive regulation of response to extracellular stimulus |
| GO: 0032103 | positive regulation of response to external stimulus |
| GO: 0032101 | regulation of response to external stimulus |
| GO: 0031589 | cell-substrate adhesion |
| GO: 0031329 | regulation of cellular catabolic process |
| GO: 0030239 | myofibril assembly |
| GO: 0030198 | extracellular matrix organization |
| GO: 0022617 | extracellular matrix disassembly |
| GO: 0022411 | cellular component disassembly |
| GO: 0019915 | lipid storage |
| GO: 0019221 | cytokine-mediated signaling pathway |
| GO: 0016567 | protein ubiquitination |
| GO: 0016259 | selenocysteine metabolic process |
| GO: 0014897 | striated muscle hypertrophy |
| GO: 0014896 | muscle hypertrophy |
| GO: 0014706 | striated muscle tissue development |
| GO: 0010761 | fibroblast migration |
| GO: 0010506 | regulation of autophagy |
| GO: 0010243 | response to organonitrogen compound |
| GO: 0010035 | response to inorganic substance |
| GO: 0009991 | response to extracellular stimulus |
| GO: 0009896 | positive regulation of catabolic process |
| GO: 0007492 | endoderm development |
| GO: 0007249 | I-kappaB kinase/NF-kappaB signaling |
| GO: 0007178 | transmembrane receptor protein serine/threonine kinase signaling pathway |
| GO: 0007006 | mitochondrial membrane organization |
| GO: 0006979 | response to oxidative stress |
| GO: 0006959 | humoral immune response |
| GO: 0006936 | muscle contraction |
| GO: 0006914 | autophagy |
| GO: 0006887 | exocytosis |
| GO: 0006839 | mitochondrial transport |
| GO: 0006754 | ATP biosynthetic process |
| GO: 0006612 | protein targeting to membrane |
| GO: 0006575 | cellular modified amino acid metabolic process |
| GO: 0006511 | ubiquitin-dependent protein catabolic process |
| GO: 0006091 | generation of precursor metabolites and energy |
| GO: 0003300 | cardiac muscle hypertrophy |
| GO: 0003012 | muscle system process |
| GO: 0001887 | selenium compound metabolic process |
| GO: 0001666 | response to hypoxia |
| GO: 0001570 | vasculogenesis |
| GO: 0001568 | blood vessel development |
| GO: 0001525 | angiogenesis |
| GO: 0001503 | ossification |
| GO: 0000956 | nuclear-transcribed mRNA catabolic process |
| GO: 0000302 | response to reactive oxygen species |
| GO: 0000165 | MAPK cascade |

**Table S1. Determination of the genome size and whole genome shotgun sequencing.**

**Table S1a. Genome size of *E. maclovinus* and *D. mawsoni* determined by flow cytometry.**

|  | 2C (pg) | 1C (pg) | Mbp |
| --- | --- | --- | --- |
| *E. maclovinus* 2 | 1.531 | 0.766 | 748.7 |
| *E. maclovinus* 4 | 1.685 | 0.842 | 823.8 |
| *E. maclovinus* 5 | 1.582 | 0.791 | 773.4 |
| *E. maclovinus* 6 | 1.585 | 0.792 | 775.0 |
|  |  | Mean | **780.23** |
|  |  | sd | **31.45** |

|  | 2C (pg) | 1C (pg) | Mbp |
| --- | --- | --- | --- |
| *D. mawsoni* 4 | 1.736887 | 0.868444 | 849.34 |
| *D. mawsoni* 5 | 1.683257 | 0.841629 | 823.11 |
| *D. mawsoni* 6 | 1.837766 | 0.918883 | 898.67 |
| *D. mawsoni* 7 | 1.731708 | 0.865854 | 846.81 |
| *D. mawsoni* 8 | 1.624532 | 0.812266 | 794.40 |
|  |  | Mean | **842.46** |
|  |  | sd | **38.44** |

**Table S1b. Sequencing libraries and sequencing depth of *D. mawsoni* genome**

| **Library** | **Insert Size (bp)** | **Clean Reads (M)** | **Total sequence length (Gb)** | **Sequence Depth (X)** |
| --- | --- | --- | --- | --- |
| **Paired-end** | ﻿170 | 295.10 | 29.51 | 38.37 |
|  | 250 | 122.50 | 12.25 | 15.93 |
|  | 500 | 290.50 | 29.05 | 37.78 |
| **Mate-pair** | 800 | 88.30 | 8.83 | 11.48 |
|  | 2K | 257.96 | 12.64 | 16.44 |
|  | 5K | 90.20 | 4.42 | 5.75 |
|  | 10K | 173.27 | 8.49 | 11.04 |
|  | 20K | 28.37 | 1.39 | 1.81 |
|  | 40K | 12.04 | 0.59 | 0.77 |
| **Sum** |  | **1,358.24** | **107.17** | **139.36** |

**Table S1c. Sequencing libraries and sequencing depth of *E. maclovinus* genome**

| **Library** | **Insert Size (bp)** | **Clean Reads (M)** | **Total sequence length (Gb)** | **Sequence Depth (X)** |
| --- | --- | --- | --- | --- |
| **Paired-end** | ﻿170 | 349.36 | 84.51 | 121.25 |
|  | 250 | 925.65 | 220.03 | 315.68 |
|  | 500 | 113.18 | 28.30 | 40.60 |
| **Mate-pair** | 3K | 61.48 | 13.88 | 19.91 |
|  | 6K | 109.93 | 24.73 | 35.48 |
|  | 10K | 173.57 | 35.06 | 50.30 |
|  | 15K | 23.53 | 4.59 | 6.59 |
|  | 20K | 0.56 | 0.13 | 0.19 |
| **Sum** |  | **1,757.26** | **411.23** | **590.00** |

**Table S2. Statistics of the genome assemblies**

**Table S2a. Genome of *D. mawsoni* assembled with SOAPdenovo and SSPACE.**

|  | **Contig** | | **Scaffold** | |
| --- | --- | --- | --- | --- |
|  | **Size (bp)** | **Number** | **Size (bp)** | **Number** |
| N90 | 4,826 | 32,862 | 202,697 | 536 |
| N80 | 8,702 | 22,391 | 526,640 | 310 |
| N70 | 12,851 | 15,893 | 910,655 | 199 |
| N60 | 17,551 | 11,294 | 1,462,269 | 133 |
| N50 | 23,122 | 7,857 | 2,216,199 | 89 |
| Largest | 246,642 | ---- | 13,813,452 | ---- |
| Total Size | 690,442,831 | ---- | 756,811,804 | ---- |
| Total Number(>=100bp) | ---- | 105,636 | ---- | 49,238 |

**Table S2b Genome of *E. maclovinus* assembled with SOAPdenovo, Platanus and SSPACE.**

|  | **Contig** | | **Scaffold** | |
| --- | --- | --- | --- | --- |
|  | **Size (bp)** | **Number** | **Size (bp)** | **Number** |
| N90 | 2,761 | 60,928 | 167,196 | 1,185 |
| N80 | 4,599 | 43,215 | 261,285 | 828 |
| N70 | 6,452 | 31,537 | 373,583 | 588 |
| N60 | 8,504 | 22,927 | 509,483 | 419 |
| N50 | 10,872 | 16,296 | 694,727 | 291 |
| Largest | 140,349 | - | 4,859,855 | - |
| Total Size | 637,281,501 | - | 744,411,660 | - |
| Total Number (>=100b) | - | 109,232 | - | 1,852 |

**Table S2c Assessment of the genome completeness.** BUSCO version is: 3.0.2. The lineage dataset is actinopterygii_odb9 (Creation date: 2016-02-13, number of species: 20, number of BUSCOs: 4584). Zebrafish is the species for the referencing orthologs.

|  | ***D. mawsoni*** | | | ***E. maclovinus*** | | |
| --- | --- | --- | --- | --- | --- | --- |
|  | Quantity | **%** | Quantity | | **%** |  |
| Complete BUSCOs | 4,453 | 97.2 | 4,352 | | 95.0 |  |
| Complete and single-copy BUSCOs | 4,345 | 94.8 | 4,202 | | 91.7 |  |
| Complete and duplicated BUSCOs | 108 | 2.4 | 150 | | 3.3 |  |
| Fragmented BUSCOs | 41 | 0.9 | 59 | | 1.3 |  |
| Missing BUSCOs | 90 | 1.9 | 173 | | 3.7 |  |
| Total BUSCO groups searched | 4,584 | 100.0 | 4,584 | | 100.0 |  |

**Table S3. Comparison of the assembled scaffolds and available genomic sequences in Genbank.**

| **Genome** | **Aligned GenBank sequences** | | | **Coverage of all matches** | **Coverage of single best match** |
| --- | --- | --- | --- | --- | --- |
|  | **Quantity** | **Total length (bp)** | **Average length (bp)** |  |  |
| ***D. mawsoni*** | 440 | 403,646 | 917 | 0.995 | 0.988 |
| ***E.maclovinus*** | 25 | 38,807 | 1,552 | 0.999 | 0.991 |

**Table S4. Statistics of heterozygous polymorphisms.** The potential loci of the SNPs were detected by uniquely aligned reads.

| ***D. mawsoni*** | **Quantity** | **Size of analyzed sequences (bp)** | **Heterozygous SNP rate (SNPs per kb)** |
| --- | --- | --- | --- |
| **Exon** | 57,556 | 37,749,387 | 1.52 |
| **Intron** | 715,856 | 252,740,314 | 2.83 |
| **Intergenic region** | 1,176,339 | 466,301,400 | 2.52 |
| **Total** | 1,949,751 | 756,791,101 | 2.58 |
|  |  |  |  |
|  |  |  |  |
| ***E. maclovinus*** | **Quantity** | **Size of analyzed sequences (bp)** | **Heterozygous SNP rate (bp per kb)** |
| **Exon** | 60,565 | 37,462,815 | 1.62 |
| **Intron** | 748,311 | 285,402,491 | 2.62 |
| **Intergenic region** | 977,956 | 421,538,929 | 2.32 |
| **Total** | 1,786,832 | 744,404,235 | 2.40 |

**Table S5. Prediction of protein-coding genes.**

| ***D. mawsoni*** | **﻿Gene set** | **Gene number** | **Average transcript length (bp)** | **Average CDS length (bp)** | **Average exon number per gene** | **Average exon length (bp)** | **Averageintron length (bp)** |
| --- | --- | --- | --- | --- | --- | --- | --- |
| ﻿De novo | AUGUSTUS | 24,166 | 12,079 | 1,578 | 8.9 | 178 | 1,335 |
|  | GENSCAN | 23,076 | 15,769 | 1,897 | 10,1 | 186 | 1,509 |
|  | D.rerio | 22,382 | 11,556 | 1,484 | 9,2 | 172 | 1,320 |
| Homolog | G.morhua | 21,891 | 11,181 | 1,382 | 8.4 | 164 | 1,320 |
|  | ﻿G.aculeatus | 22,467 | 11,625 | 1,448 | 8.9 | 164 | 1,297 |
|  | H.sapiens | 18,859 | 12,028 | 1,470 | 8.9 | 166 | 1,340 |
|  | O.latipes | 21,914 | 10,977 | 1,425 | 8.6 | 166 | 1,259 |
|  | T.rubripes | 20,364 | 12,668 | 1,555 | 9.4 | 165 | 1,317 |
| **Final GLEAN gene models** | | **﻿****22,516** | **12,903** | **1,676** | **9.3** | **180.7** | **1,355** |

| ***E. maclovinus*** | **Gene set** | **Gene number** | **Average transcript length (bp)** | **Average CDS length (bp)** | **Average exon number per gene** | **Average exon length** | **Average intron length (bp)** |
| --- | --- | --- | --- | --- | --- | --- | --- |
| Denovo | AUGUSTUS | 23,134 | 15009.8 | 1,539 | 9.1 | 168.6 | 1,657 |
|  | Genscan | 27,075 | 18150.49 | 1,593 | 8.9 | 179.2 | 2,099 |
| Homolog | D. rerio | 21,019 | 11150.24 | 1,423 | 8.4 | 168.9 | 1,309 |
|  | G. morhua | 20,568 | 10737.68 | 1,329 | 8.2 | 162.6 | 1,310 |
|  | G.aculeatus | 21,077 | 11247.46 | 1,394 | 8.6 | 162.4 | 1,300 |
|  | H.sapiens | 20,580 | 10451.87 | 1,289 | 7.9 | 164.1 | 1,336 |
|  | O. latipes | 20,472 | 10712.42 | 1,376 | 8.4 | 163.9 | 1,262 |
|  | T. rubripes | 19,256 | 12235.38 | 1,492 | 9.1 | 163.5 | 1,323 |
| **Final GLEAN gene models** | | **22,959** | **15388.43** | **1,546** | **9.2** | **168.6** | **1,694** |

**Table S6. Prediction of conserved non-coding RNAs.**

**Table S6a. *D. mawsoni*.**

| **Type** | | **Copy nomber** | **Average length(bp)** | **Total length(bp)** | **% of genome** |
| --- | --- | --- | --- | --- | --- |
| miRNA | | 295 | 82.33 | 24287 | 0.003263 |
| tRNA | | 2010 | 77.63 | 156,036 | 0.020619 |
| rRNA | rRNA | 110 | ﻿155.06 | 17,057 | 0.002254 |
|  | 18S | ﻿26 | 206.19 | 5,361 | 0.000708 |
|  | 28S | ﻿65 | 158.48 | 10,301 | 0.001361 |
|  | ﻿5.8S | 5 | 97.8 | 489 | 0.000065 |
|  | 5S | 14 | 64.71 | 906 | 0.00012 |
| snRNA | snRNA | ﻿422 | 114.94 | 48,506 | 0.006409 |
|  | CD-box | ﻿219 | 90.37 | 19,790 | 0.002615 |
|  | HACA-boc | ﻿119 | 142.13 | 16,913 | 0.002235 |
|  | splicing | ﻿77 | 146.18 | 11,256 | 0.001487 |

**Table S6b. *E. maclovinus*.**

| **Type** | | **Copy number** | **Average length(bp)** | **Total length(bp)** | **% of genome** |
| --- | --- | --- | --- | --- | --- |
| miRNA | | 286 | 82.33 | 23546 | 0.003164 |
| tRNA | | 1133 | 76.48 | 86651 | 0.011639 |
| rRNA | rRNA | 19 | 61.42 | 1167 | 0.000157 |
|  | 18S | 12 | 48.67 | 584 | 0.000078 |
|  | 28S | 7 | 83.29 | 583 | 0.000078 |
| snRNA | snRNA | 364 | 116.63 | 42455 | 0.005703 |
|  | CD-box | 202 | 96.51 | 19495 | 0.002619 |
|  | HACA-boc | 92 | 145.61 | 13396 | 0.0018 |
|  | Unknown | 4 | 57 | 228 | 0.000031 |
|  | scaRNA | 7 | 187.29 | 1311 | 0.000176 |
|  | splicing | 59 | 136.02 | 8025 | 0.001078 |

**Table S7. Comparison of copy numbers for the predicted miRNAs.**

| miRNA | Dma | Ema |  | miRNA | Dma | Ema |  | miRNA | Dma | Ema |
| --- | --- | --- | --- | --- | --- | --- | --- | --- | --- | --- |
| mir-1 | 3 | 3 |  | mir-203 | 1 | 1 |  | mir-50 | 1 | 0 |
| mir-10 | 8 | 10 |  | mir-204 | 3 | 2 |  | mir-548 | 3 | 2 |
| mir-101 | 2 | 2 |  | mir-205 | 2 | 2 |  | mir-551 | 1 | 1 |
| mir-103 | 3 | 3 |  | mir-208 | 1 | 1 |  | mir-552 | 1 | 2 |
| mir-122 | 1 | 1 |  | mir-21 | 2 | 2 |  | mir-556 | 0 | 1 |
| mir-124 | 5 | 6 |  | mir-210 | 1 | 1 |  | mir-576 | 0 | 1 |
| mir-126 | 1 | 1 |  | mir-214 | 2 | 3 |  | mir-577 | 0 | 1 |
| mir-1265 | 1 | 0 |  | mir-216 | 2 | 3 |  | mir-578 | 1 | 0 |
| mir-128 | 2 | 2 |  | mir-217 | 2 | 3 |  | mir-583 | 1 | 1 |
| mir-129 | 4 | 4 |  | mir-218 | 1 | 1 |  | mir-590 | 0 | 1 |
| mir-130 | 1 | 1 |  | mir-219 | 4 | 4 |  | mir-592 | 2 | 0 |
| mir-1306 | 1 | 1 |  | mir-22 | 3 | 3 |  | mir-598 | 4 | 5 |
| mir-132 | 4 | 4 |  | mir-221 | 4 | 4 |  | mir-609 | 2 | 0 |
| mir-133 | 3 | 3 |  | mir-223 | 2 | 2 |  | mir-651 | 1 | 0 |
| mir-135 | 5 | 5 |  | mir-23 | 5 | 5 |  | mir-653 | 1 | 0 |
| mir-137 | 3 | 3 |  | mir-24 | 7 | 5 |  | mir-7 | 4 | 4 |
| mir-138 | 3 | 3 |  | mir-25 | 1 | 1 |  | mir-70 | 1 | 0 |
| mir-1388 | 1 | 1 |  | mir-26 | 3 | 4 |  | mir-75 | 0 | 2 |
| mir-139 | 1 | 1 |  | mir-268 | 1 | 4 |  | mir-785 | 0 | 6 |
| mir-140 | 1 | 1 |  | mir-27 | 7 | 6 |  | mir-8 | 2 | 2 |
| mir-142 | 2 | 2 |  | mir-29 | 9 | 11 |  | mir-879 | 1 | 0 |
| mir-143 | 1 | 1 |  | mir-296 | 0 | 1 |  | mir-883 | 1 | 0 |
| mir-144 | 2 | 1 |  | mir-2973 | 1 | 0 |  | mir-9 | 7 | 8 |
| mir-145 | 1 | 1 |  | mir-2985-2 | 4 | 3 |  | mir-92 | 2 | 2 |
| mir-146 | 1 | 1 |  | mir-30 | 4 | 1 |  | mir-942 | 1 | 2 |
| mir-147 | 1 | 1 |  | mir-301 | 3 | 3 |  | mir-944 | 4 | 0 |
| mir-148 | 2 | 2 |  | mir-31 | 1 | 1 |  | mir-96 | 2 | 3 |
| mir-15 | 1 | 2 |  | mir-316 | 0 | 1 |  | mir-983 | 1 | 0 |
| mir-153 | 4 | 4 |  | mir-328 | 1 | 2 |  | MIR1023 | 8 | 2 |
| mir-155 | 1 | 1 |  | mir-33 | 3 | 2 |  | MIR1027 | 1 | 3 |
| mir-16 | 3 | 2 |  | mir-338 | 3 | 4 |  | MIR1122 | 4 | 9 |
| mir-17 | 6 | 5 |  | mir-34 | 1 | 1 |  | MIR1446 | 1 | 0 |
| mir-181 | 4 | 4 |  | mir-340 | 1 | 0 |  | MIR158 | 1 | 0 |
| mir-182 | 2 | 3 |  | mir-365 | 1 | 1 |  | MIR162_2 | 1 | 0 |
| mir-183 | 2 | 3 |  | mir-375 | 1 | 1 |  | MIR2118 | 6 | 9 |
| mir-184 | 2 | 2 |  | miR-430 | 3 | 0 |  | MIR405 | 1 | 1 |
| mir-187 | 1 | 1 |  | mir-434 | 1 | 0 |  | MIR475 | 1 | 0 |
| mir-19 | 4 | 4 |  | mir-449 | 1 | 0 |  | MIR476 | 1 | 0 |
| mir-190 | 3 | 3 |  | mir-451 | 2 | 1 |  | MIR477 | 1 | 0 |
| mir-192 | 1 | 1 |  | mir-454 | 1 | 1 |  | MIR530 | 6 | 6 |
| mir-193 | 1 | 1 |  | mir-455 | 2 | 2 |  | MIR535 | 1 | 0 |
| mir-194 | 2 | 2 |  | mir-456 | 1 | 1 |  | MIR807 | 1 | 0 |
| mir-196 | 3 | 3 |  | mir-458 | 1 | 0 |  | MIR811 | 2 | 1 |
| mir-197 | 1 | 2 |  | mir-460 | 1 | 1 |  | MIR815 | 1 | 0 |
| mir-199 | 4 | 4 |  | mir-489 | 1 | 1 |  | MIR820 | 2 | 0 |
| mir-202 | 1 | 1 |  | mir-499 | 1 | 1 |  | MIR821 | 1 | 2 |

**Total 109 112**

**Table S8. Summary of predicted tRNA genes in *D. mawsoni*, *E. maclovinus* and stickleback.**

| **Type** | ***D. mawsoni*** | ***E. maclovinus*** | **Stickleback** |
| --- | --- | --- | --- |
| tRNAs decoding Standard 20 AA | 1,097 | 1,037 | 2,767 |
| Selenocysteine tRNAs (TCA) | 84 | 1 | 4 |
| Possible suppressor tRNAs (CTA,TTA) | 2 | 3 | 0 |
| tRNAs with undetermined/unknown isotypes | 7 | 5 | 39 |
| Predicted pseudogenes | 820 | 87 | 1,242 |
| **Total tRNAs** | **2,010** | **1,133** | **4,052** |

**Table S9. Statistics of annotated transposable elements.**

***D. mawsoni E. maclovinus N. coriiceps***

|  | **Length occupied (bp)** | **Percentage of seuqences** | **Length occupied (bp)** | **Percentage of seuqences** | **Length occupied (bp)** | **Percentage of seuqences** |
| --- | --- | --- | --- | --- | --- | --- |
| **Class I elements (Retroelements)** | **40,817,518** | **5.39** | **18,093,350** | **2.43** | **26,110,271** | **4.10** |
| **LTR Retrotransposon** | **14,327,191** | **1.89** | **2,527,650** | **0.34** | **4143346** | **0.65** |
| LTR/Copia | 79,713 | 0.01 | 108,339 | 0.01 | 0 | 0.00 |
| LTR/DIRS | 10,053,220 | 1.33 | 70,952 | 0.01 | 1696988 | 0.27 |
| LTR/ERV1 | 641,728 | 0.08 | 138,705 | 0.02 | 135263 | 0.02 |
| LTR/ERVK | 114,909 | 0.02 | 542 | 0 | 0 | 0.00 |
| LTR/ERVL | 780 | 0 | 1,214 | 0 | 0 | 0.00 |
| LTR/ERVL-MaLR | 167 | 0 | 239 | 0 | 0 | 0.00 |
| LTR/Gypsy | 2,962,617 | 0.39 | 1,372,112 | 0.18 | 1853083 | 0.29 |
| LTR/Pao | 473,722 | 0.06 | 835,406 | 0.11 | 426660 | 0.07 |
| unclassified LTR | 335 | 0 | 141 | 0 | 31352 | 0.00 |
| **non-LTR Retrotransposon** | **26,490,327** | **3.5** | **15,565,700** | **2.09** | **21966925** | **3.45** |
| **LINE** | **23,580,424** | **3.12** | **12,525,125** | **1.68** | **19195850** | **3.02** |
| LINE/CR1 | 66,546 | 0.01 | 60,201 | 0.01 | 0 | 0.00 |
| LINE/Dong-R4 | 52 | 0 | 40 | 0 | 0 | 0.00 |
| LINE/I | 1,549,848 | 0.2 | 0 | 0 | 1522311 | 0.24 |
| LINE/I-Jockey | 1,358 | 0 | 1,678 | 0 | 335166 | 0.05 |
| LINE/I-Nimb | 263,819 | 0.03 | 0 | 0 | 63630 | 0.01 |
| LINE/Jockey | 37,606 | 0 | 0 | 0 | 36070 | 0.01 |
| LINE/L1 | 488,689 | 0.06 | 250,592 | 0.03 | 239480 | 0.04 |
| LINE/L1-Tx1 | 92,263 | 0.01 | 342,676 | 0.05 | 46398 | 0.01 |
| LINE/L2 | 12,658,578 | 1.67 | 4,518,039 | 0.61 | 11877569 | 1.87 |
| LINE/Penelope | 78,340 | 0.01 | 11,786 | 0 | 0 | 0.00 |
| LINE/R1 | 0 | 0 | 202,089 | 0.03 | 0 | 0.00 |
| LINE/Proto2 | 42,012 | 0.01 | 0 | 0 | 80366 | 0.01 |
| LINE/RTE-BovB | 1,473,943 | 0.19 | 2,328,209 | 0.31 | 1174468 | 0.18 |
| LINE/RTE-X | 69,164 | 0.01 | 61,584 | 0.01 | 109342 | 0.02 |
| LINE/Rex-Babar | 6,700,578 | 0.89 | 4,748,231 | 0.64 | 3442883 | 0.54 |
| unclassified LINE | 57,628 | 0.01 | 0 | 0 | 268167 | 0.04 |
| **SINE** | **2,909,903** | **0.38** | **3,040,575** | **0.41** | **2771075** | **0.44** |
| SINE/5S-Deu-L2 | 127,057 | 0.02 | 34,496 | 0 | 0 | 0.00 |
| SINE/Alu | 96 | 0 | 53 | 0 | 0 | 0.00 |
| SINE/ID | 364 | 0 | 3,658 | 0 | 4487 | 0.00 |
| SINE/MIR | 1,504,488 | 0.2 | 429,953 | 0.06 | 1331185 | 0.21 |
| SINE/tRNA | 34,095 | 0 | 179,533 | 0.02 | 0 | 0.00 |
| SINE/tRNA-Core | 262,078 | 0.03 | 414,731 | 0.06 | 179381 | 0.03 |
| SINE/tRNA-Deu | 6,988 | 0 | 1,641 | 0 | 0 | 0.00 |
| SINE/tRNA-Core-L2 | 0 | 0 | 270,657 | 0.04 | 417755 | 0.07 |
| SINE/tRNA-L1 | 81,243 | 0.01 | 181,536 | 0.02 | 93116 | 0.01 |
| SINE/tRNA-Meta | 39,870 | 0.01 | 0 | 0 | 0 | 0.00 |
| SINE/tRNA-RTE | 3,011 | 0 | 1,811 | 0 | 18596 | 0.00 |
| SINE/tRNA-V | 25,789 | 0 | 23,119 | 0 | 8918 | 0.00 |
| unclassified SINE | 824,824 | 0.11 | 1,499,387 | 0.2 | 717637 | 0.11 |
| **Class II elements**  **(DNA Transposons)** | **70,348,175** | **9.3** | **32,755,031** | **4.4** | **65,319,149** | **10.26** |
| **DNA Transposon** | **67,948,424** | **8.98** | **32,323,176** | **4.34** | **63,740,109** | **10.01** |
| DNA/Academ | 133,982 | 0.02 | 50,491 | 0.01 | 175,888 | 0.03 |
| DNA/CMC-EnSpm | 1,742,985 | 0.23 | 661,983 | 0.09 | 2,564,874 | 0.40 |
| DNA/Crypton | 648 | 0 | 0 | 0 | 611,201 | 0.10 |
| DNA/Crypton-A | 464,106 | 0.06 | 57,293 | 0.01 | 69,622 | 0.01 |
| DNA/Crypton-V | 108,507 | 0.01 | 1,970,401 | 0.26 | 1,008,716 | 0.16 |
| DNA/IS3EU | 952,363 | 0.13 | 52,658 | 0.01 | 562,168 | 0.09 |
| DNA/Kolobok | 0 | 0 | 477,558 | 0.06 | 227,078 | 0.04 |
| DNA/Kolobok-T2 | 8,446,473 | 1.12 | 2,017,996 | 0.27 | 6,568,064 | 1.03 |
| DNA/MULE-MuDR | 83 | 0 | 67,309 | 0.01 | 27,352 | 0.00 |
| DNA/Merlin | 61,904 | 0.01 | 55,188 | 0.01 | 0 | 0.00 |
| DNA/Novosib | 178,402 | 0.02 | 0 | 0 | 4,316,481 | 0.68 |
| DNA/P | 890,265 | 0.12 | 1,086,603 | 0.15 | 616,349 | 0.10 |
| DNA/PIF-Harbinger | 3,425,729 | 0.45 | 642,849 | 0.09 | 3,113,471 | 0.49 |
| DNA/PIF-ISL2EU | 0 | 0 | 95,757 | 0.01 | 78,638 | 0.01 |
| DNA/PiggyBac | 1,698,132 | 0.22 | 140,879 | 0.02 | 986,728 | 0.15 |
| DNA/Sola-1 | 674,922 | 0.09 | 0 | 0 | 3,296,333 | 0.52 |
| DNA/TcMar-Fot1 | 131,748 | 0.02 | 138,649 | 0.02 | 458,263 | 0.07 |
| DNA/TcMar-ISRm11 | 230,922 | 0.03 | 227,721 | 0.03 | 254,353 | 0.04 |
| DNA/TcMar-Mariner | 202 | 0 | 270 | 0 | 0 | 0.00 |
| DNA/TcMar-Stowaway | 48,379 | 0.01 | 0 | 0 | 154,190 | 0.02 |
| DNA/TcMar-Tc1 | 1,597,002 | 0.21 | 5,873,578 | 0.79 | 4,202,312 | 0.66 |
| DNA/TcMar-Tc2 | 65,735 | 0.01 | 852 | 0 | 662,703 | 0.10 |
| DNA/TcMar-Tigger | 20,443 | 0 | 22,370 | 0 | 0 | 0.00 |
| DNA/Zisupton | 2,380,123 | 0.31 | 16,543 | 0 | 667,073 | 0.10 |
| DNA/Zisupton-  hAT-hybrid | 46,370 | 0.01 | 0 | 0 | 0 | 0.00 |
| DNA/hAT | 805,382 | 0.11 | 98,693 | 0.01 | 1,338,936 | 0.21 |
| DNA/hAT-Ac | 20,011,330 | 2.64 | 10,339,116 | 1.39 | 17,437,166 | 2.74 |
| DNA/hAT-Blackjack | 631,434 | 0.08 | 179,641 | 0.02 | 797,445 | 0.13 |
| DNA/hAT-Charlie | 8,831,272 | 1.17 | 2,988,961 | 0.4 | 6,401,806 | 1.01 |
| DNA/hAT-Tag1 | 129,278 | 0.02 | 2,376 | 0 | 243,639 | 0.04 |
| DNA/hAT-Tip100 | 4,067,714 | 0.54 | 1,559,670 | 0.21 | 5,071,981 | 0.80 |
| DNA/hAT-Tol2 | 0 | 0 | 143,569 | 0.02 | 0 | 0.00 |
| DNA/hAT-hAT5 | 911,646 | 0.12 | 7,114 | 0 | 1,117,811 | 0.18 |
| DNA/hAT-hAT6 | 65,626 | 0.01 | 0 | 0 | 10,624 | 0.00 |
| DNA/hAT-hobo | 74,483 | 0.01 | 92,409 | 0.01 | 151,899 | 0.02 |
| Unclassified DNA  Transposon | 9,120,834 | 1.21 | 3,254,679 | 0.44 | 546,945 | 0.09 |
| **RC/Helitron** | **2,399,751** | **0.32** | **431,855** | **0.06** | **1,579,040** | **0.25** |
| **Unknown repeats** | **50,661,137** | **6.69** | **23,746,117** | **3.19** | **53,001,935** | **8.33** |
| **Total transposable elements** | **161,826,830** | **21.38** | **74,594,498** | **10.02** | **144,431,355** | **22.69** |

**Table S10. Simple sequence repeats types in two notothenioids**

**S10a Summary of simple sequence repeat (SSR) types and numbers in *D. mawsoni***

| ***D. mawsoni*** | **Repeat unit number (bp)** | **Total Loci** | **Total length (bp)** | **Average unit number** | **Average length (bp)** |
| --- | --- | --- | --- | --- | --- |
| **Mononucleotide** | 40 or less | 1,865 | 55,130 | 29.6 | 29.6 |
|  | 41 to 80 | 628 | 34,931 | 55.6 | 55.6 |
|  | 81 or more | 46 | 6,264 | 136.2 | 136.2 |
|  | total | 2,539 | 96,325 | 37.9 | 37.9 |
|  |  |  |  |  |  |
| **Dinucleotide** | 30 or less | 51,693 | 2,195,328 | 21.2 | 42.5 |
|  | 31 to 60 | 35,953 | 2,939,473 | 40.9 | 81.8 |
|  | 61 or more | 5,061 | 1,031,123 | 101.9 | 203.7 |
|  | total | 92,707 | 6,165,924 | 33.3 | 66.5 |
|  |  |  |  |  |  |
| **Trinucleotide** | 20 or less | 6,950 | 253,710 | 12.2 | 36.5 |
|  | 21 to 40 | 1,311 | 108,800 | 27.7 | 83.0 |
|  | 41 or more | 594 | 217,468 | 122.0 | 366.1 |
|  | total | 8,855 | 579,978 | 21.8 | 65.5 |
|  |  |  |  |  |  |
| **Tetranucleotide** | 16 or less | 6,096 | 263,686 | 10.8 | 43.3 |
|  | 17 to 32 | 4,170 | 381,831 | 22.9 | 91.6 |
|  | 33 or more | 2,572 | 871,239 | 84.7 | 338.7 |
|  | total | 12,838 | 1,516,756 | 29.5 | 118.1 |
|  |  |  |  |  |  |
| **Pentanucleotide** | 12 or less | 1,485 | 54,411 | 7.3 | 36.6 |
|  | 13 to 24 | 375 | 31,573 | 16.8 | 84.2 |
|  | 25 or more | 357 | 183,178 | 102.6 | 513.1 |
|  | total | 2,217 | 269,162 | 24.3 | 121.4 |
|  |  |  |  |  |  |
| **Hexanucleotide** | 10 or less | 2,257 | 93,314 | 6.9 | 41.3 |
|  | 11 to 20 | 1,537 | 134,273 | 14.6 | 87.4 |
|  | 21 or more | 825 | 215,114 | 43.5 | 260.7 |
|  | total | 4,619 | 442,701 | 16.0 | 95.8 |
| **Total/Average** |  | 123,775 | 9,070,846 | **31.4** | **73.3** |

**Table S10b** **Summary of simple sequence repeat (SSR) types and numbers in *E. maclovinus***

| ***E. maclovinus*** | **Repeat unit number (bp)** | **Total Loci** | **Total length (bp)** | **Average unit number** | **Average length (bp)** |
| --- | --- | --- | --- | --- | --- |
| **Mononucleotide** | 40 or less | 1,409 | 45,307 | 32.2 | 32.2 |
|  | 41 to 80 | 463 | 22,353 | 48.3 | 48.3 |
|  | 81 or more | 20 | 2,084 | 104.2 | 104.2 |
|  | total | 1,892 | 69,744 | 36.9 | 36.9 |
|  |  |  |  |  |  |
| **Dinucleotide** | 30 or less | 50,395 | 2,135,472 | 21.2 | 42.4 |
|  | 31 to 60 | 40,410 | 3,302,529 | 40.9 | 81.7 |
|  | 61 or more | 3,598 | 533,338 | 74.1 | 148.2 |
|  | total | 94,403 | 5,971,339 | 31.6 | 63.3 |
|  |  |  |  |  |  |
| **Trinucleotide** | 20 or less | 7,242 | 277,979 | 12.8 | 38.4 |
|  | 21 to 40 | 2,194 | 180,319 | 27.4 | 82.2 |
|  | 41 or more | 836 | 223,422 | 89.1 | 267.3 |
|  | total | 10,272 | 681,720 | 22.1 | 66.4 |
|  |  |  |  |  |  |
| **Tetranucleotide** | 16 or less | 4,166 | 169,891 | 10.2 | 40.8 |
|  | 17 to 32 | 2,413 | 222,649 | 23.1 | 92.3 |
|  | 33 or more | 933 | 420,514 | 112.7 | 450.7 |
|  | total | 7,512 | 813,054 | 27.1 | 108.2 |
|  |  |  |  |  |  |
| **Pentanucleotide** | 12 or less | 769 | 29,278 | 7.6 | 38.1 |
|  | 13 to 24 | 232 | 20,013 | 17.3 | 86.3 |
|  | 25 or more | 193 | 74,470 | 77.2 | 385.9 |
|  | total | 1,194 | 123,761 | 20.7 | 103.7 |
|  |  |  |  |  |  |
| **Hexanucleotide** | 10 or less | 1,360 | 56,519 | 6.9 | 41.6 |
|  | 11 to 20 | 1,189 | 106,615 | 14.9 | 89.7 |
|  | 21 or more | 467 | 129,225 | 46.1 | 276.7 |
|  | total | 3,016 | 292,359 | 16.2 | 96.9 |
| **Total/Average** |  | 118,289 | 7,951,977 | **30.1** | **67.2** |

**Table S11 Functional annotation of genes under positive selection.**

**Table S11a Prediction of conserved function domains in the genes under positive selection.** The typical 10 out of 526 genes are listed. The items in red are mentioned in the main text. The full list of 526 genes can be downloaded from the official website (http://202.121.66.128/).

| **COGID** | **D.ma gene ID (Dissostichus_mawsoni_GLEAN_XXXXXXXX)** | **Ref. gene ID** | **Gene description** | **Interpro Domains** |
| --- | --- | --- | --- | --- |
| COG10016 | 10005541 | ENSG00000140474 | ULK3 | IPR000719; Protein kinase, catalytic domain |
| COG10039 | 10012587 | N/A | si:zfos-80g12.1 | IPR006730; PA26 p53-induced protein (sestrin) |
| COG10080 | 10021912 | N/A | gga3 | IPR000626; Ubiquitin |
| COG10140 | 10003730 | N/A | N/A | N/A |
| COG10145 | 10006840 | ENSG00000105655 | ISYNA1 | IPR002587; Myo-inositol-1-phosphate synthase |
| COG14223 | 10021857 | N/A | Perilipin-2-like | IPR004279; Perilipin |
| COG3040 | 10021766 | ENSG00000154556 | sorbs2a | IPR001452; Src homology-3 domain |
| COG3290 | 10021869 | ENSG00000161533 | acox1 | IPR002655; Acyl-CoA oxidase, C-terminal |
| COG4685 | 10019763 | N/A | apoa1a | IPR000074; Apolipoprotein A1/A4/E |
| COG4689 | 10009682 | ENSG00000116171 | scp2a | IPR003033; SCP2 sterol-binding domain |
| COG7037 | 10014850 | ENSG00000116783 | tnni3k | IPR000719; Protein kinase, catalytic domain |

**Table S11b. KEGG pathway enrichment of the *D. mawsoni* genes under positive selection.**

| **Ko entry** | **Gene quantity** | ***p* value** | **Description** |
| --- | --- | --- | --- |
| ko04974 | 12 | 5.1E-05 | Protein digestion and absorption |
| ko04210 | 9 | 3.6E-03 | Apoptosis |
| ko04611 | 9 | 8.5E-03 | Platelet activation |
| ko02010 | 4 | 8.9E-03 | ABC transporters |
| ko04024 | 13 | 1.3E-02 | cAMP signaling pathway |
| ko00310 | 6 | 1.6E-02 | Lysine degradation |
| ko05164 | 11 | 1.6E-02 | Influenza A |
| ko00072 | 3 | 1.7E-02 | Synthesis and degradation of ketone bodies |
| ko04512 | 6 | 3.0E-02 | ECM-receptor interaction |
| ko04620 | 8 | 3.0E-02 | Toll-like receptor signaling pathway |
| ko04151 | 19 | 3.3E-02 | PI3K-Akt signaling pathway |
| ko04060 | 10 | 3.5E-02 | Cytokine-cytokine receptor interaction |
| ko00280 | 6 | 3.6E-02 | Valine, leucine and isoleucine degradation |
| ko00604 | 3 | 4.0E-02 | Glycosphingolipid biosynthesis - ganglio series |
| ko00564 | 8 | 4.6E-02 | Glycerophospholipid metabolism |

**Table S11c. GO term enrichment of the *D. mawsoni* genes under positive selection.** Three functional classes are biological processes (BP), molecular function (MF) and cellular component (CC).

| **GO term** | **Gene quantity** | ***p* value** | **Name** | **Class** |
| --- | --- | --- | --- | --- |
| GO:0005515 | 156 | 6.0E-04 | protein binding | MF |
| GO:0005581 | 3 | 7.4E-04 | collagen trimer | CC |
| GO:0005201 | 3 | 2.8E-03 | extracellular matrix structural constituent | MF |
| GO:0071426 | 2 | 8.2E-03 | ribonucleoprotein complex export from nucleus | BP |
| GO:0071166 | 2 | 8.2E-03 | ribonucleoprotein complex localization | BP |
| GO:0006308 | 2 | 8.2E-03 | DNA catabolic process | BP |
| GO:0051168 | 2 | 8.2E-03 | nuclear export | BP |
| GO:0009070 | 2 | 8.2E-03 | serine family amino acid biosynthetic process | BP |
| GO:0016049 | 5 | 8.3E-03 | cell growth | BP |
| GO:1901607 | 4 | 1.8E-02 | alpha-amino acid biosynthetic process | BP |
| GO:0001558 | 4 | 1.8E-02 | regulation of cell growth | BP |
| GO:0008652 | 4 | 1.8E-02 | cellular amino acid biosynthetic process | BP |
| GO:0000280 | 5 | 1.9E-02 | nuclear division | BP |
| GO:0016746 | 9 | 2.0E-02 | transferase activity, transferring acyl groups | MF |
| GO:0070011 | 19 | 2.2E-02 | peptidase activity, acting on L-amino acid peptides | MF |
| GO:0004198 | 2 | 2.3E-02 | calcium-dependent cysteine-type endopeptidase activity | MF |
| GO:0008536 | 2 | 2.3E-02 | Ran GTPase binding | MF |
| GO:0009067 | 2 | 2.3E-02 | aspartate family amino acid biosynthetic process | BP |
| GO:0015924 | 2 | 2.3E-02 | mannosyl-oligosaccharide mannosidase activity | MF |
| GO:0004571 | 2 | 2.3E-02 | mannosyl-oligosaccharide 1,2-alpha-mannosidase activity | MF |
| GO:0048285 | 5 | 2.4E-02 | organelle fission | BP |
| GO:0004175 | 13 | 2.4E-02 | endopeptidase activity | MF |
| GO:0040008 | 4 | 2.5E-02 | regulation of growth | BP |
| GO:0046394 | 5 | 3.0E-02 | carboxylic acid biosynthetic process | BP |
| GO:0016053 | 5 | 3.0E-02 | organic acid biosynthetic process | BP |
| GO:0044265 | 9 | 3.0E-02 | cellular macromolecule catabolic process | BP |
| GO:0051082 | 6 | 3.1E-02 | unfolded protein binding | MF |
| GO:0019899 | 5 | 3.6E-02 | enzyme binding | MF |
| GO:0005488 | 270 | 4.0E-02 | binding | MF |
| GO:0008233 | 19 | 4.1E-02 | peptidase activity | MF |
| GO:0050661 | 2 | 4.3E-02 | NADP binding | MF |
| GO:1901700 | 2 | 4.3E-02 | response to oxygen-containing compound | BP |
| GO:0000775 | 2 | 4.3E-02 | chromosome, centromeric region | CC |
| GO:0009066 | 2 | 4.3E-02 | aspartate family amino acid metabolic process | BP |

**Table S12 The *D. mawsoni* DEGs involved in the fatty acid oxidation.**

| **Human gene ID** | **Human gene** | **Human homolog description** | **Dma gene ID (Dissostichus_mawsoni_ GLEAN_XXXXXXXX)** | **log2 (DmaFPKM / EmaFPKM)** | **log2 (DmaFPKM / NcoFPKM)** | **Dma gene description** |
| --- | --- | --- | --- | --- | --- | --- |
| ENSG00000173208 | ABCD2 | ATP binding cassette subfamily D member 2 | 10020742 | 4.48 | 1.00 | ATP binding cassette subfamily D member 2 |
| ENSG00000117528 | ABCD3 | ATP binding cassette subfamily D member 3 | 10018782 | 2.26 | 1.00 | ATP-binding cassette, sub-family D (ALD), member 3b |
|  | ACACB | acetyl-CoA carboxylase beta | 10016838 | 1.00 | 2.05 | acacb |
|  | ACAD8 | acyl-CoA dehydrogenase family member 8 | 10010242 | 1.00 | 2.71 | acad8 |
| ENSG00000240303 | ACAD11 | acyl-CoA dehydrogenase family member 11 | 10012910 | 5.97 |  | acyl-CoA dehydrogenase family, member 11 |
| ENSG00000177646 | ACAD9 | acyl-CoA dehydrogenase family member 9 | 10004704 | -2.40 |  | acyl-CoA dehydrogenase family, member 9 |
|  | ACADM | acyl-CoA dehydrogenase | 10013798 | 1.00 | 6.01 | acadm |
|  | ACADS | acyl-CoA dehydrogenase | 10004750 | 1.00 | 3.63 | NA |
| ENSG00000115361 | ACADL | acyl-CoA dehydrogenase | 10011795 | -2.95 | 2.80 | acyl-CoA dehydrogenase, long chain |
| ENSG00000196177 | ACADSB | acyl-CoA dehydrogenase | 10012057 | -3.50 | 2.28 | acyl-CoA dehydrogenase, short/branched chain |
|  | ACOT8 | acyl-CoA thioesterase 8 | 10003590 | 1.00 | 3.91 | acot8 |
| ENSG00000072778 | ACADVL | acyl-CoA dehydrogenase | 10009334 | -3.49 | 5.30 | acyl-CoA dehydrogenase, very long chain |
| ENSG00000161533 | ACOX1 | acyl-CoA oxidase 1 | 10021869 | 4.27 | 2.47 | acyl-CoA oxidase 1, palmitoyl |
| ENSG00000159346 | ADIPOR1 | adiponectin receptor 1 | 10019476 | 3.34 | 6.99 | adiponectin receptor 2 |
|  | CPT1A | carnitine palmitoyltransferase 1A | 10019264 | 1.00 | 2.35 | bm |
| ENSG00000105221 | AKT2 | AKT serine/threonine kinase 2 | 10019793 | 3.71 | 3.68 | v-akt murine thymoma viral oncogene homolog 2 |
| ENSG00000095321 | CRAT | carnitine O-acetyltransferase | 10020129 | -4.10 | 1.00 | immediate early response 5-like |
| ENSG00000005469 | CROT | carnitine O-octanoyltransferase | 10009430 | -4.14 | -3.04 | carnitine O-octanoyltransferase |
| ENSG00000062282 | DGAT2 | diacylglycerol O-acyltransferase 2 | 10007476 | 5.80 | 1.00 | diacylglycerol O-acyltransferase 2 |
| ENSG00000282853 | ECH1 | enoyl-CoA hydratase 1 | 10005435 | -4.63 | 3.09 | enoyl CoA hydratase 1, peroxisomal |
| ENSG00000127884 | ECHS1 | enoyl-CoA hydratase | 10020572 | -6.59 | 1.00 | enoyl CoA hydratase, short chain, 1, mitochondrial |
| ENSG00000167969 | ECI1 | enoyl-CoA delta isomerase 1 | 10003913 | -4.48 | 2.37 | enoyl-CoA delta isomerase 1 |
| ENSG00000198721 | ECI2 | enoyl-CoA delta isomerase 2 | 10012859 | -4.93 | 7.80 | enoyl-CoA delta isomerase 2 |
| ENSG00000113790 | EHHADH | enoyl-CoA hydratase and 3-hydroxyacyl CoA dehydrogenase | 10018400 | 2.40 | 1.00 | enoyl-CoA, hydratase/3-hydroxyacyl CoA dehydrogenase |
| ENSG00000140374 | ETFA | electron transfer flavoprotein alpha subunit | 10016034 | -7.70 | 1.00 | electron-transfer-flavoprotein, alpha polypeptide |
| ENSG00000105379 | ETFB | electron transfer flavoprotein beta subunit | 10011072 | -7.60 | 1.00 | electron-transfer-flavoprotein, beta polypeptide |
| ENSG00000163586 | FABP1 | fatty acid binding protein 1 | 10020264 | 5.26 | 1.00 | fatty acid binding protein 1b, tandem duplicate 1 |
|  | HADHA | hydroxyacyl-CoA dehydrogenase/3-ketoacyl-CoA thiolase/enoyl-CoA hydratase (trifunctional protein) | 10013990 | 1.00 | 2.04 | NA |
| ENSG00000105607 | GCDH | glutaryl-CoA dehydrogenase | 10013011 | -3.67 | 1.00 | glutaryl-CoA dehydrogenase a |
| ENSG00000138029 | HADHB | hydroxyacyl-CoA dehydrogenase/3-ketoacyl-CoA thiolase/enoyl-CoA hydratase (trifunctional protein) | 10014128 | -6.31 | 2.43 | hydroxyacyl-CoA dehydrogenase/3-ketoacyl-CoA thiolase/enoyl-CoA hydratase (trifunctional protein), beta subunit |
| ENSG00000185950 | IRS2 | insulin receptor substrate 2 | 10004145 | 1.67 | 5.98 | insulin receptor substrate 2b |
|  | MLYCD | malonyl-CoA decarboxylase | 10014757 | 1.00 | 2.00 | mlycd |
| ENSG00000128928 | IVD | isovaleryl-CoA dehydrogenase | 10021395 | -2.98 | 3.16 | isovaleryl-CoA dehydrogenase |
| ENSG00000124370 | MCEE | methylmalonyl-CoA epimerase | 10009917 | -8.35 | 1.00 | methylmalonyl CoA epimerase |
|  | MUT | methylmalonyl-CoA mutase | 10013513 | 1.00 | 1.90 | mut |
| ENSG00000198793 | MTOR | mechanistic target of rapamycin | 10009502 | 5.73 | 1.00 | mechanistic target of rapamycin (serine/threonine kinase) |
| ENSG00000119508 | NR4A3 | nuclear receptor subfamily 4 group A member 3 | 10009406 | 4.45 | 1.00 | nuclear receptor subfamily 4, group A, member 3 |
| ENSG00000114054 | PCCB | propionyl-CoA carboxylase beta subunit | 10013952 | -3.68 | 1.00 | propionyl CoA carboxylase, beta polypeptide |
| ENSG00000107537 | PHYH | phytanoyl-CoA 2-hydroxylase | 10020805 | -2.48 | 1.54 | phytanoyl-CoA 2-hydroxylase |
| ENSG00000146070 | PLA2G7 | phospholipase A2 group VII | 10003120 | 2.18 | 1.00 | phospholipase A2, group VII (platelet-activating factor acetylhydrolase, plasma) |
| ENSG00000127948 | POR | cytochrome p450 oxidoreductase | 10019734 | 2.77 | 4.21 | P450 (cytochrome) oxidoreductase a |
| ENSG00000186951 | PPARA | peroxisome proliferator activated receptor alpha | 10018616 | 2.24 | 1.00 | peroxisome proliferator-activated receptor alpha |
|  | PPARD | peroxisome proliferator activated receptor delta | 10013150 | 1.00 | 3.82 | bm |
| ENSG00000132170 | PPARG | peroxisome proliferator activated receptor gamma | 10011527 | 5.67 | 6.20 | peroxisome proliferator-activated receptor gamma |
| ENSG00000100372 | SLC25A17 | solute carrier family 25 member 17 | 10011291 | 1.29 | 1.00 | solute carrier family 25 (mitochondrial carrier; peroxisomal membrane protein), member 17 |
|  | SCP2 | sterol carrier protein 2 | 10009682 | 1.00 | 4.00 | scp2a |

**Table S13 The upregulated *D. mawsoni* DEGs involved in the lipid storage pathway.** The gene expressions (FPKM) were compared between *D. mawsoni* and *E.maclovinus* (log2(*D. ma*/*E.ma*)) and between *D. mawsoni* and *N.coriiceps* (log2(*D. ma*/ *N.co*)).

| GO:0010883 | regulation of lipid storage | |  |
| --- | --- | --- | --- |
| **Humangene ID** | **Human gene name** | **log2(*D.ma*/*E.ma*)** | **log2(*D.ma*/*N.co*)** |
| ENSG00000175445 | LPL | 6.17 | 8.63 |
| ENSG00000177666 | PNPLA2 | 7.40 | 8.64 |
| ENSG00000100906 | NFKBIA | 3.47 | 4.46 |
| ENSG00000132170 | PPARG | 5.67 | 6.69 |
| ENSG00000165029 | ABCA1 | 4.19 | 4.90 |
| ENSG00000197296 | FITM2 | 2.63 | 2.66 |
| ENSG00000025434 | NR1H3 | 4.14 | 3.63 |
| ENSG00000163874 | ZC3H12A | 3.41 | 5.21 |
| ENSG00000144909 | OSBPL11 | 4.08 | NS |
| ENSG00000109670 | FBXW7 | 5.21 | NS |
| ENSG00000186951 | PPARA | 2.24 | NS |
| ENSG00000259207 | ITGB3 | 5.41 | NS |
| ENSG00000106484 | MEST | -3.26 | NS |
| ENSG00000011198 | ABHD5 | 4.19 | NS |
| ENSG00000136244 | IL6 | 4.09 | NS |
| ENSG00000084674 | APOB | 4.06 | NS |
| ENSG00000091039 | OSBPL8 | 3.97 | NS |

**Table S14. Functional annotation of the *D. mawsoni* muscle DEG genes.** The gene expressions (FPKM) were compared between *D. mawsoni* and *E.maclovinus* (log2(Dma/Ema)) and between *D. mawsoni* and *N.coriiceps* (log2(Dma/Nco)).

| **Human gene ID** | **Human gene** | **Human gene description** | **Dma gene ID (Dissostichus_mawsoni_GLEAN_)** | **log2(Dma/Ema)** | **log2(Dma/Nco)** | **Dma gene description** |
| --- | --- | --- | --- | --- | --- | --- |
| ENSG00000100219 | XBP1 | X-box binding protein 1 [Source:HGNC Symbol;Acc:HGNC:12801] | 10004979 | 3.34 | 3.83 | xbp1 |
| ENSG00000186480 | INSIG1 | insulin induced gene 1 [Source:HGNC Symbol;Acc:HGNC:6083] | 10012898 | 3.66 | 4.39 | insig2 |
| ENSG00000104332 | SFRP1 | secreted frizzled related protein 1 [Source:HGNC Symbol;Acc:HGNC:10776] | 10002906 | 8.36 | 1.00 | sfrp1a |
| ENSG00000132170 | PPARɤ | peroxisome proliferator activated receptor gamma [Source:HGNC Symbol;Acc:HGNC:9236] | 10011527 | 5.67 | 8.99 | pparg |
| ENSG00000105329 | TGFB1 | transforming growth factor beta 1 [Source:HGNC Symbol;Acc:HGNC:11766] | 10003272 | 4.82 | 5.50 | tgfb1a |
| ENSG00000073756 | PTGS2 | prostaglandin-endoperoxide synthase 2 [Source:HGNC Symbol;Acc:HGNC:9605] | 10017497 | 7.67 | 6.10 | ptgs2b |
| ENSG00000275031 | METRNL | meteorin like | 10014510 | 7.60 | 4.67 | metrnl |
| ENSG00000163874 | ZC3H12A | zinc finger CCCH-type containing 12A [Source:HGNC Symbol;Acc:HGNC:26259] | 10011114 | 3.41 | 5.20 | zc3h12a |
| ENSG00000115738 | ID2 | inhibitor of DNA binding 2 | 10021376 | 4.64 | 3.97 | id2a |
| ENSG00000143970 | ASXL2 | additional sex combs like 2 | 10013991 | -4.75 | 1.00 | asxl2 |
| ENSG00000104375 | STK3 | serine/threonine kinase 3 [Source:HGNC Symbol;Acc:HGNC:11406] | 10019895 | 2.34 | 4.37 | stk3 |
| ENSG00000142453 | CARM1 | coactivator associated arginine methyltransferase 1 [Source:HGNC Symbol;Acc:HGNC:23393] | 10000873 | 4.18 | 1.00 | carm1 |
| ENSG00000096717 | SIRT1 | sirtuin 1 [Source:HGNC Symbol;Acc:HGNC:14929] | 10007928 | 1.11 | 1.00 | NA |
| ENSG00000137413 | TAF8 | TATA-box binding protein associated factor 8 [Source:HGNC Symbol;Acc:HGNC:17300] | 10007247 | 1.17 | 1.00 | taf8 |
| ENSG00000174705 | SH3PXD2B | SH3 and PX domains 2B [Source:HGNC Symbol;Acc:HGNC:29242] | 10011900 | 5.09 | 4.15 | sh3pxd2b |
| ENSG00000198793 | MTOR | mechanistic target of rapamycin [Source:HGNC Symbol;Acc:HGNC:3942] | 10009502 | 5.73 | 1.00 | mtor |
| ENSG00000019549 | SNAI2 | snail family transcriptional repressor 2 [Source:HGNC Symbol;Acc:HGNC:11094] | 10020956 | 2.70 | 5.59 | snai2 |
| ENSG00000171456 | ASXL1 | additional sex combs like 1 | 10015148 | 7.83 | 1.00 | asxl1 |
| ENSG00000162998 | FRZB | frizzled-related protein [Source:HGNC Symbol;Acc:HGNC:3959] | 10001272 | 5.30 | 1.00 | frzb |
| ENSG00000118260 | CREB1 | cAMP responsive element binding protein 1 [Source:HGNC Symbol;Acc:HGNC:2345] | 10017611 | 2.35 | 2.67 | creb1a |
| ENSG00000114315 | HES1 | hes family bHLH transcription factor 1 [Source:HGNC Symbol;Acc:HGNC:5192] | 10012307 | 1.97 | 1.00 | her6 |
| ENSG00000166949 | SMAD3 | SMAD family member 3 [Source:HGNC Symbol;Acc:HGNC:6769] | 10017104 | 2.73 | 1.00 | NA |
| ENSG00000085276 | MECOM | MDS1 and EVI1 complex locus [Source:HGNC Symbol;Acc:HGNC:3498] | 10013104 | 4.81 | 1.00 | mecom |
| ENSG00000283100 | SIRT2 | sirtuin 2 [Source:HGNC Symbol;Acc:HGNC:10886] | 10013141 | 2.32 | 1.00 | sirt2 |
| ENSG00000176624 | MEX3C | mex-3 RNA binding family member C [Source:HGNC Symbol;Acc:HGNC:28040] | 10020222 | 2.79 | 1.00 | MEX3C |
| ENSG00000150907 | FOXO1 | forkhead box O1 [Source:HGNC Symbol;Acc:HGNC:3819] | 10022149 | 4.06 | 1.00 | foxo1b |
| ENSG00000101384 | JAG1 | jagged 1 [Source:HGNC Symbol;Acc:HGNC:6188] | 10016959 | 5.91 | 1.00 | jag1b |
| ENSG00000140044 | JDP2 | Jun dimerization protein 2 [Source:HGNC Symbol;Acc:HGNC:17546] | 10014955 | 1.54 | 1.00 | NA |
| ENSG00000149926 | FAM57B | family with sequence similarity 57 member B [Source:HGNC Symbol;Acc:HGNC:25295] | 10018294 | 2.26 | 1.00 | FAM57B |
| ENSG00000071575 | TRIB2 | tribbles pseudokinase 2 [Source:HGNC Symbol;Acc:HGNC:30809] | 10013503 | 1.27 | 1.00 | trib2 |
| ENSG00000136244 | IL6 | interleukin 6 [Source:HGNC Symbol;Acc:HGNC:6018] | 10020027 | 4.09 | 1.00 | NA |
| ENSG00000079102 | RUNX1T1 | RUNX1 translocation partner 1 [Source:HGNC Symbol;Acc:HGNC:1535] | 10019957 | 3.55 | 1.00 | cbfa2t2 |
| ENSG00000032742 | IFT88 | intraflagellar transport 88 [Source:HGNC Symbol;Acc:HGNC:20606] | 10007033 | 3.66 | 1.00 | ift88 |
| ENSG00000134243 | SORT1 | sortilin 1 [Source:HGNC Symbol;Acc:HGNC:11186] | 10013734 | 4.23 | 1.00 | sort1b |

**Table S15. Functional annotation of *D. mawsoni* DEGs involved in chondrogensis and osteogenesis.** The gene expressions (FPKM) were compared between *D. mawsoni* and *T. bernacchii* (T.be) (log2(D.am/Tbe)) and between *D. mawsoni* and *P. bochgrevinki* (P.bo) (log2(D.am/P.bo)). The genes in yellow highlight are mentioned in the main text.

| **Human gene ID** | **Human gene name** | **Human gene description** | **D.ma gene ID (**Dissostichus_ mawsoni_GLEAN_XXXXXXXX**)** | **D.ma FPKM** | **P.bo FPKM** | **T.be FPKM** | **log2 (D.ma / P.bo)** | **log2 (D.ma / T.be)** | **D.ma gene symbol** | **D.ma gene description** |
| --- | --- | --- | --- | --- | --- | --- | --- | --- | --- | --- |
| ENSG00000115268 | RPS15 | ribosomal protein S15 | 10021210 | 3,169.3 | 37,328.1 | 17,989.2 | -3.56 | -2.50 | rps15 | ribosomal protein S15 |
| ENSG00000113140 | SPARC | secreted protein acidic and cysteine rich | 10000646 | 1,689.5 | 445.1 | 413.4 | 1.92 | 2.03 | NA | NA |
| ENSG00000204628 | RACK1 | receptor for activated C kinase 1 | 10007696 | 603.2 | 11,049.3 | 3,843.8 | -4.20 | -2.67 | si:dkeyp-44a8.4 | collagen, type XXIII, alpha 1 |
| ENSG00000141232 | TOB1 | transducer of ERBB2 | 10003640 | 388.7 | 179.6 | 6.3 | 1.11 | 5.95 | tob1b | transducer of ERBB2, 1b |
| ENSG00000070404 | FSTL3 | follistatin like 3 | 10016217 | 272.7 | 0.0 | 1.8 | 9.09 | 7.27 | fstl3 | follistatin-like 3 (secreted glycoprotein) |
| ENSG00000171223 | JUNB | JunB proto-oncogene | 10010125 | 218.4 | 91.9 | 33.1 | 1.25 | 2.72 | junba | jun B proto-oncogene a |
| ENSG00000087245 | MMP2 | matrix metallopeptidase 2 | 10015293 | 177.7 | 17.5 | 18.2 | 3.35 | 3.29 | mmp2 | matrix metallopeptidase 2 |
| ENSG00000100985 | MMP9 | matrix metallopeptidase 9 | 10013749 | 152.3 | 0.0 | 4.8 | 8.25 | 4.99 | mmp9 | matrix metallopeptidase 9 |
| ENSG00000143367 | TUFT1 | tuftelin 1 | 10009453 | 99.8 | 25.2 | 9.5 | 1.99 | 3.39 | tuft1a | tuftelin 1a |
| ENSG00000067560 | RHOA | ras homolog family member A | 10010775 | 107.9 | 36.1 | 17.3 | 1.58 | 2.64 | rhoab | ras homolog gene family, member Ab |
| ENSG00000157227 | MMP14 | matrix metallopeptidase 14 | 10008761 | 83.4 | 7.6 | 9.8 | 3.45 | 3.09 | mmp14b | matrix metallopeptidase 14b |
| ENSG00000118523 | CTGF | connective tissue growth factor | 10009900 | 78.9 | 7.3 | 12.9 | 3.44 | 2.61 | ctgfa | connective tissue growth factor a |
| ENSG00000143387 | CTSK | cathepsin K | 10007295 | 91.3 | 7.1 | 30.3 | 3.68 | 1.59 | ctsk | cathepsin K |
| ENSG00000178585 | CTNNBIP1 | catenin beta interacting protein 1 | 10013698 | 62.2 | 0.0 | 0.0 | 6.96 | 6.96 | ctnnbip1 | catenin, beta interacting protein 1 |
| ENSG00000142871 | CYR61 | cysteine rich angiogenic inducer 61 | 10017451 | 64.2 | 19.0 | 15.8 | 1.76 | 2.02 | cyr61 | cysteine-rich, angiogenic inducer, 61 |
| ENSG00000073756 | PTGS2 | prostaglandin-endoperoxide synthase 2 | 10017497 | 51.1 | 1.6 | 6.4 | 5.01 | 3.00 | ptgs2b | prostaglandin-endoperoxide synthase 2b |
| ENSG00000153187 | HNRNPU | heterogeneous nuclear ribonucleoprotein U | 10009004 | 40.3 | 5.2 | 1.9 | 2.95 | 4.43 | hnrnpua | heterogeneous nuclear ribonucleoprotein Ua |
| ENSG00000104332 | SFRP1 | secreted frizzled related protein 1 | 10002906 | 51.0 | 2.6 | 15.5 | 4.27 | 1.72 | sfrp1a | secreted frizzled-related protein 1a |
| ENSG00000081189 | MEF2C | myocyte enhancer factor 2C | 10021592 | 35.0 | 9.6 | 1.6 | 1.87 | 4.43 | mef2cb | myocyte enhancer factor 2cb |
| ENSG00000100644 | HIF1A | hypoxia inducible factor 1 alpha subunit | 10021410 | 38.3 | 3.4 | 6.0 | 3.47 | 2.66 | hif-1a | hypoxia-inducible factor 1 alpha (hif-1a), mRNA |
| ENSG00000137745 | MMP13 | matrix metallopeptidase 13 | 10019803 | 27.8 | 0.0 | 0.0 | 5.80 | 5.80 | mmp13b | matrix metallopeptidase 13b |
| ENSG00000278788 | SBNO2 | strawberry notch homolog 2 | 10016234 | 29.2 | 0.0 | 0.0 | 7.69 | 7.00 | sbno2a | strawberry notch homolog 2a |
| ENSG00000078804 | TP53INP2 | tumor protein p53 inducible nuclear protein 2 | 10015884 | 26.0 | 1.7 | 2.7 | 3.93 | 3.28 | NA | NA |
| ENSG00000109906 | ZBTB16 | zinc finger and BTB domain containing 16 | 10010233 | 22.8 | 3.2 | 0.0 | 2.82 | 5.51 | zbtb16b | zinc finger and BTB domain containing 16b |
| ENSG00000115541 | HSPE1 | heat shock protein family E (Hsp10) member 1 | 10001793 | 19.2 | 342.3 | 140.9 | -4.15 | -2.87 | hspe1 | heat shock 10 protein 1 |
| ENSG00000142156 | COL6A1 | collagen type VI alpha 1 chain | 10008788 | 20.7 | 4.4 | 0.0 | 2.25 | 4.72 | col6a1 | collagen, type VI, alpha 1 |
| ENSG00000168487 | BMP1 | bone morphogenetic protein 1 | 10016732 | 19.9 | 1.4 | 0.0 | 3.79 | 5.68 | bmp1a | bone morphogenetic protein 1a |
| ENSG00000167244 | IGF2 | insulin like growth factor 2 | 10019268 | 18.2 | 0.0 | 2.1 | 5.18 | 3.08 | igf2b | insulin-like growth factor 2b |
| ENSG00000103855 | CD276 | CD276 molecule | 10015322 | 20.6 | 6.3 | 4.5 | 1.71 | 2.20 | cd276 | CD276 molecule |
| ENSG00000170365 | SMAD1 | SMAD family member 1 | 10021713 | 18.0 | 0.0 | 2.5 | 5.10 | 2.83 | smad1 | SMAD family member 1 |
| ENSG00000134352 | IL6ST | interleukin 6 signal transducer | 10005727 | 16.1 | 1.3 | 0.0 | 3.67 | 4.30 | il6st | interleukin 6 signal transducer |
| ENSG00000105894 | PTN | pleiotrophin | 10016204 | 1.9 | 50.9 | 44.3 | -4.76 | -4.56 | ptn | pleiotrophin |
| ENSG00000019186 | CYP24A1 | cytochrome P450 family 24 subfamily A member 1 | 10019375 | 14.8 | 4.3 | 0.0 | 1.79 | 4.89 | cyp24a1 | cytochrome P450, family 24, subfamily A, polypeptide 1 |
| ENSG00000164828 | SUN1 | Sad1 and UNC84 domain containing 1 | 10015602 | 16.1 | 1.0 | 2.3 | 3.98 | 2.80 | sun1 | Sad1 and UNC84 domain containing 1 |
| ENSG00000107779 | BMPR1A | bone morphogenetic protein receptor type 1A | 10010378 | 13.4 | 0.0 | 0.0 | 4.74 | 4.74 | bmpr1aa | bone morphogenetic protein receptor, type IAa |
| ENSG00000198732 | SMOC1 | SPARC related modular calcium binding 1 | 10009582 | 13.0 | 0.0 | 0.0 | 4.64 | 4.70 | smoc1 | SPARC related modular calcium binding 1 |
| ENSG00000143369 | ECM1 | extracellular matrix protein 1 | 10008908 | 11.5 | 0.0 | 0.0 | 4.52 | 4.52 | ecm1b | extracellular matrix protein 1b |
| ENSG00000138623 | SEMA7A | semaphorin 7A (John Milton Hagen blood group) | 10016006 | 10.9 | 0.0 | 0.0 | 4.45 | 4.28 | sema7a | semaphorin 7A |
| ENSG00000142798 | HSPG2 | heparan sulfate proteoglycan 2 | 10005135 | 10.6 | 1.5 | 0.0 | 2.80 | 6.10 | hspg2 | heparan sulfate proteoglycan 2 |
| ENSG00000142627 | EPHA2 | EPH receptor A2 | 10004870 | 10.8 | 1.2 | 1.6 | 3.13 | 2.76 | NA | NA |
| ENSG00000105329 | TGFB1 | transforming growth factor beta 1 | 10003272 | 9.8 | 2.0 | 1.1 | 2.30 | 3.20 | tgfb1a | transforming growth factor, beta 1a |
| ENSG00000046653 | GPM6B | glycoprotein M6B | 10015518 | 12.4 | 4.2 | 4.1 | 1.54 | 1.61 | gpm6ba | glycoprotein M6Ba |
| ENSG00000154122 | ANKH | ANKH inorganic pyrophosphate transport regulator | 10018873 | 10.2 | 3.0 | 2.4 | 1.78 | 2.10 | ankha | ANKH inorganic pyrophosphate transport regulator a |
| ENSG00000151348 | EXT2 | exostosin glycosyltransferase 2 | 10022504 | 11.0 | 3.6 | 3.2 | 1.60 | 1.79 | ext2 | exostosin glycosyltransferase 2 |
| ENSG00000156427 | FGF18 | fibroblast growth factor 18 | 10010771 | 7.6 | 0.0 | 0.0 | 3.93 | 3.93 | fgf18a | fibroblast growth factor 18a |

**Table S16. KEGG pathway enrichment of the *D. mawsoni* DEGs involved in osteoclast differentiation.**

| **ID** | **Description** | **Gene count** | **Gene Ratio** | **Bg Ratio** | ***p* value** | ***q* value** |
| --- | --- | --- | --- | --- | --- | --- |
| hsa03010 | Ribosome | 61 | 61/563 | 91/3268 | 1.0E-26 | 2.7E-24 |
| hsa05145 | Toxoplasmosis | 16 | 16/563 | 45/3268 | 2.3E-03 | 2.4E-01 |
| hsa04512 | ECM-receptor interaction | 13 | 13/563 | 34/3268 | 2.8E-03 | 2.4E-01 |
| hsa04610 | Complement and coagulation cascades | 10 | 10/563 | 24/3268 | 4.1E-03 | 2.4E-01 |
| hsa04060 | Cytokine-cytokine receptor interaction | 18 | 18/563 | 56/3268 | 4.4E-03 | 2.4E-01 |
| hsa04932 | Non-alcoholic fatty liver disease (NAFLD) | 22 | 22/563 | 76/3268 | 7.2E-03 | 2.9E-01 |
| hsa04141 | Protein processing in endoplasmic reticulum | 30 | 30/563 | 113/3268 | 7.5E-03 | 2.9E-01 |
| hsa05418 | Fluid shear stress and atherosclerosis | 21 | 21/563 | 73/3268 | 9.3E-03 | 3.1E-01 |
| hsa05152 | Tuberculosis | 20 | 20/563 | 70/3268 | 1.2E-02 | 3.5E-01 |
| hsa05010 | Alzheimer's disease | 25 | 25/563 | 94/3268 | 1.4E-02 | 3.7E-01 |
| hsa04659 | Th17 cell differentiation | 13 | 13/563 | 41/3268 | 1.7E-02 | 3.9E-01 |
| hsa04380 | Osteoclast differentiation | 17 | 17/563 | 59/3268 | 1.8E-02 | 3.9E-01 |
| hsa05134 | Legionellosis | 9 | 9/563 | 25/3268 | 1.9E-02 | 3.9E-01 |
| hsa05205 | Proteoglycans in cancer | 27 | 27/563 | 107/3268 | 2.1E-02 | 4.0E-01 |
| hsa04630 | Jak-STAT signaling pathway | 14 | 14/563 | 47/3268 | 2.3E-02 | 4.0E-01 |
| hsa03060 | Protein export | 7 | 7/563 | 18/3268 | 2.4E-02 | 4.0E-01 |
| hsa04350 | TGF-beta signaling pathway | 14 | 14/563 | 48/3268 | 2.7E-02 | 4.2E-01 |
| hsa04145 | Phagosome | 17 | 17/563 | 62/3268 | 2.9E-02 | 4.2E-01 |
| hsa04137 | Mitophagy - animal | 12 | 12/563 | 40/3268 | 3.2E-02 | 4.2E-01 |
| hsa05416 | Viral myocarditis | 8 | 8/563 | 23/3268 | 3.2E-02 | 4.2E-01 |
| hsa04657 | IL-17 signaling pathway | 11 | 11/563 | 36/3268 | 3.5E-02 | 4.2E-01 |
| hsa05412 | Arrhythmogenic right ventricular cardiomyopathy (ARVC) | 11 | 11/563 | 36/3268 | 3.5E-02 | 4.2E-01 |
| hsa05012 | Parkinson's disease | 18 | 18/563 | 69/3268 | 4.0E-02 | 4.7E-01 |
| hsa05160 | Hepatitis C | 16 | 16/563 | 61/3268 | 4.9E-02 | 5.4E-01 |

**Table S17. Functional annotation of the *D. mawsoni* DEGs involved in osteoclast differentiation.** The gene expressions (FPKM) were compared between *D. mawsoni* and *T. bernacchii* (log2(D.ma/T.be)) and between *D. mawsoni* and *P. bochgrevinki* (log2(D.ma/P.bo)).

| **Human gene ID** | **Human gene** | **Human gene Descripton** | ***D.ma* gene ID (**Dissostichus_mawsoni_GLEAN_XXXXXXXX**)** | ***D.ma* FPKM** | ***P.bo* FPKM** | ***T.be* FPKM** | **log2(D.ma/ P.bo)** | **log2(D.ma/ T.be)** | ***D.ma* gene symbol** |
| --- | --- | --- | --- | --- | --- | --- | --- | --- | --- |
| ENSG00000171223 | JUNB | JunB proto-oncogene | 10010125 | 218.36 | 91.91 | 33.06 | 1.25 | 2.72 | junba |
| ENSG00000100906 | NFKBIA | NFKB inhibitor alpha [Source:HGNC Symbol;Acc:HGNC:7797] | 10013547 | 169.91 | 22.91 | 51.46 | 2.89 | 1.72 | nfkbiaa |
| ENSG00000075426 | FOSL2 | FOS like 2 | 10022067 | 68.90 | 3.73 | 1.19 | 4.21 | 5.85 | fosl2 |
| ENSG00000143387 | CTSK | cathepsin K [Source:HGNC Symbol;Acc:HGNC:2536] | 10007295 | 91.32 | 7.12 | 30.32 | 3.68 | 1.59 | ctsk |
| ENSG00000184557 | SOCS3 | suppressor of cytokine signaling 3 [Source:HGNC Symbol;Acc:HGNC:19391] | 10015549 | 61.71 | 29.37 | 8.05 | 1.07 | 2.94 | socs3 |
| ENSG00000162434 | JAK1 | Janus kinase 1 [Source:HGNC Symbol;Acc:HGNC:6190] | 10017477 | 33.16 | 2.16 | 2.23 | 3.94 | 3.89 | jak1 |
| ENSG00000221823 | PPP3R1 | protein phosphatase 3 regulatory subunit B | 10006143 | 29.04 | 13.85 | 3.08 | 1.07 | 3.24 | ppp3r1a |
| ENSG00000161011 | SQSTM1 | sequestosome 1 [Source:HGNC Symbol;Acc:HGNC:11280] | 10000506 | 41.39 | 13.99 | 14.30 | 1.57 | 1.53 | NA |
| ENSG00000083799 | CYLD | CYLD lysine 63 deubiquitinase [Source:HGNC Symbol;Acc:HGNC:2584] | 10015273 | 26.90 | 0.00 | 0.00 | 4.79 | 5.75 | cylda |
| ENSG00000105397 | TYK2 | tyrosine kinase 2 [Source:HGNC Symbol;Acc:HGNC:12440] | 10014578 | 24.68 | 4.56 | 4.17 | 2.44 | 2.56 | tyk2 |
| ENSG00000115594 | IL1R1 | interleukin 1 receptor type 1 [Source:HGNC Symbol;Acc:HGNC:5993] | 10006571 | 21.08 | 0.00 | 6.44 | 5.57 | 1.71 | NA |
| ENSG00000077150 | NFKB2 | nuclear factor kappa B subunit 2 [Source:HGNC Symbol;Acc:HGNC:7795] | 10002381 | 14.73 | 1.13 | 0.00 | 3.70 | 6.18 | nfkb2 |
| ENSG00000055208 | TAB2 | TGF-beta activated kinase 1/MAP3K7 binding protein 2 [Source:HGNC Symbol;Acc:HGNC:17075] | 10000451 | 13.69 | 3.44 | 1.52 | 1.99 | 3.18 | tab2 |
| ENSG00000163513 | TGFBR2 | transforming growth factor beta receptor 2 [Source:HGNC Symbol;Acc:HGNC:11773] | 10011439 | 16.82 | 2.53 | 5.43 | 2.73 | 1.63 | tgfbr2 |
| ENSG00000142166 | IFNAR1 | interferon alpha and beta receptor subunit 1 [Source:HGNC Symbol;Acc:HGNC:5432] | 10001737 | 10.39 | 0.00 | 1.01 | 4.04 | 3.36 | il10rb |
| ENSG00000105329 | TGFB1 | transforming growth factor beta 1 [Source:HGNC Symbol;Acc:HGNC:11766] | 10003272 | 9.79 | 1.99 | 1.06 | 2.30 | 3.20 | tgfb1a |
| ENSG00000259207 | ITGB3 | integrin subunit beta 3 [Source:HGNC Symbol;Acc:HGNC:6156] | 10018365 | 7.72 | 0.00 | 0.00 | 3.72 | 3.95 | itgb3b |

**References for SI citations**

Li, R. *et al*. The sequence and de novo assembly of the giant panda genome. ***Nature*** 463, 311-317 (2009).

Murchison, E.P. *et al*. Genome Sequencing and Analysis of the *Tasmanian Devil* and Its Transmissible Cancer. ***Cell*** 148, 780-791 (2012).

Yang, Z. (2007) PAML 4: phylogenetic analysis by maximum likelihood. *Mol Biol Evol* 24:1586-91
