# Supplementary material for: The genomic basis for colonizing the freezing Southern Ocean revealed by Antarctic toothfish and Patagonian robalo genomes
Source: Gigascience. 2019 Jan 31;8(4):giz016. doi: 10.1093/gigascience/giz016 (PMC6457430; doi:10.1093/gigascience/giz016)
Supplement: Response_to_Reviewer_Comments_Original_Submission.pdf [file giz016_response_to_reviewer_comments_original_submission.pdf]

Author's Response To Reviewer Comments

Close

Response to reviewers' comments

Reviewer #1: Review of "Genomic bases for colonizing the freezing Southern Ocean revealed by the genomes
of Antarctic toothfish and Patagonia robalo"
This manuscript sequences the genome and transcriptome of Antarctic tootfish and its closest temperate relative, the Patagonian robalo. The authors detect >200 protein gene families that have expanded with functions in stress response and freeze resistance. The amount of work conducted in this study is impressive. I applaud the authors for estimating the genome size using both the k-mer frequency distribution and flow cytometry to compare to the final assembly lengths. I am particularly interested in the expansion of transposable elements correlate to notothenioid diversification. I find no major flaws with the study, but would like to see more detail on the assembly steps (e.g. parameters tested and used in SoapDeNovo and SSPACE).

Minor comments:
Suggest a review for spelling and English grammar throughout the paper.
Thank you for your suggestion. The manuscript has been edited by two professors teach in US universities (Prof. Chi-Hing C. Cheng, University of Illinois, and Prof. George Somero, Stanford University who are native English speakers).

L69: is the >90% catch from commercial fisheries?
No, it is from many studies based on random sampling in the Southern Ocean. We added this information

L141: do the authors know why both the scaffold and contig N50 lengths are so much lower in robalo compared to toothfish?

For both species, collected red blood cells were embedded into agarose plugs till isolation of the genomic DNA by the same protocol. However, the Robalo DNA for some unidentified reasons is easier to degrade, which resulted in lower molecular weight of isolated genomics DNA in robalo,which is approximately 25 kb compared to 40 kb in toothfish. Accordingly, sizes of the constructed sequencing libraries ranged from 170 to 40,000 bp in toothfish, while 170 to 20,000 bp in robalo. Thus spans of the Matepair reads used in scaffolding for the robalo assemblies are smaller than those of the toothfish so that the final scaffold N50 length of the robalo genome is lower. Althouth we considerably increased the sequencing depth of the paired-end libraries in robalo, size of the assembled contigs were still lower.
.
In Fig. 4 "kidney" is spelled incorrectly on the "caudal kidney" label
Thanks. This label in Fig.4 has been corrected in the revision.

Reviewer #2: In this study, Chen and colleagues did genome sequencing of two notothenioids to understand genetic basis of Antarctic notothenioids adaptation to the Southern Ocean. I do think that the methodology of the study is sound and findings here are solid. The genomic resources are valuable for further studying genetic basis of the notothenioids adaptive radiation as the authors state. Before recommendation for its final publishing in GigaScience, I have the following concerns that authors might consider. 1) The authors might consider to incorporate the earlier sequenced notothenioid genome. I see that the authors have included the Antarctic bullhead notothen genome in their comparison analyses but do make fair comparison among the three notothenioid genomes. For example, the authors speculated that TE might contribute to genome size increasing in derived notothenioids by comparison between Antarctic

toothfish and Patagonia robalo. What about TEs in the Antarctic
bullhead notothen genome? What about LINEs in Antarctic bullhead notothen genome? A through comparison among the three notothenioid genomes might give us more information about the topic the authors are trying to beat.

Thank you for suggestion. Comparison of the TEs among Antarctic toothfish, Patagonia robalo and Antarctic bullhead notothen genomes has been added to this revision. Accumulation of TEs are observed in both Antarctic toothfish and bullhead notothen genomes. We thus modified the previous statement regarding TE content and genome size as follows: "The doubling of TE content in the D. mawsoni and N. coriiceps genomes suggests higher activity of TEs in theAntarctic species in relative to the basal robalo, suggesting a likely contributing factor to the observed trend of increasing genome sizes in more derived Antarctic notothenioid lineages". As for as the timing of LINE insertion is concerned, we calculated their insertion time in the N. coriiceps genome by the same methodology as in D. mawsoni and E. maclovinus. A similar trend of expansion is observed, in D. mawsoni and N. coriiceps. The corresponding results has been added to Fig.2a in the revision.

2) As findings in their earlier works, the authors find that gene duplication plays an important role in adaptation to the freezing Southern Ocea in notothenioids. I am wondering how many gene families experienced duplication have been identified by both this study and their 2008 PNAS study.

As we stated in the manuscript, "Due to inherent inefficiency in correctly assembling highly similar DNA sequences in the shotgun sequencing strategy, there are likely many more duplicated genes that had eluded detection". From the set of duplicated genes we identified through comparative genome hybridization (Chen et al., 2008) previously, We found 23 protein coding genes are shown significantly duplicated in D. mawsoni genome in relative to E. maclovinus which was shown in Additional file 1: Fig. S4b. Among these genes included zona pellucida domain containing protein C5 (ZPC5), multiple banded antigen (previously a novel gene), serum lectin isoform 1 precursor (previously FBP32II) and hepcidin. Many types of ZPs, such as ZPAX1, ZPC1, ZPC2 failed to detect as duplicated in this study, but are known to undergone substantial duplication through array-based genome hybridization and quantitative PCR (Cao et al., 2016),indicating the limitation of the shotgun genome sequencing strategy in finding gene duplications.

References
28. Chen, Z. et al. Transcriptomic and genomic evolution under constant cold in Antarctic notothenioid fish. Proc. Natl. Acad. Sci. USA 105, 12944-12949 (2008).
29 Xu, Q. et al. Adaptive evolution of hepcidin genes in antarctic notothenioid fishes. Mol. Biol. Evol. 25, 1099-1112 (2008).
30 Cao, L. et al. Neofunctionalization of zona pellucida proteins enhances freeze-prevention in the eggs of Antarctic notothenioids. Nat. Commun. 7, 12987 (2016).

3) The authors used an RNA-seq method for their study of transcriptomic adaptation to the freezing environment. However, I do not see any details how they collected the tissues, as we all know that such analysis is very sensitive to the sampling strategy.

Thank you for your suggestion. We added the following information to the Materials and Methods section:

"To obtain tissues from the large-sized D. mawsoni, live specimen was anesthetized with MS222 (tricaine methanesulfonate) inside a ambient seawater filled floating sheet plastic tubing in the aquarium tank. The anesthetized specimen was then put on a V-shaped trough for dissection. Tissues were quickly removed and cut into small pieces on ice, and immediately immersed and shaken in ≥10 volumes of pre-chilled (-20℃) 90% ethanol (made with 100% pure ethanol and sterilized MilliQ Type 1 water). The ethanol was replaced with a fresh volume within 10 minutes, and again at 2-3 hours and 12 hours later. This preservation method serially desiccates the tissue and effectively inactivates tissue nucleases. The tissue samples were kept in -20℃ freezer throughout the serial preservation process and then stored at -20℃ until use. To obtain tissues from E. maclovinus, MS222 anesthetized specimen was quickly dissected on ice,

and preserved in -20℃ as described for D. mawsoni. The ethanol preserved tissues were shipped back to the University of Illinois on dry ice."

Reviewer #3: Reviewer report.

Title: Genomic bases for colonizing the freezing Southern Ocean revealed by the genomes of Antarctic toothfish and Patagonia robalo

General comments
The authors have sequenced and assembled the genomes of two notothenioids, and have done extensive comparisons with regards to expansions of gene families and differential expression of genes. They show that several genes in the D. mawsoni has undergone positive selection, highlighting the evolution of the genes of that species.

Specific comments

Abstract: An extant species is not necessary a proxy for an extinct species.
Thank you for your suggestion. The sentence that mentioned the proxy is in Introduction. We corrected it according to the reviewer's comment in revision.

Introduction:
Line 89-90: You specify "whole genome sequence analysis" as the criteria for mentioning the Antarctic rockcod as the only notothenioid reported so far, but Malmstrøm et al 2016 (https://www.nature.com/articles/ng.3645) did publish genomic sequences and the assembly of Chaenocephalus aceratus. However, they did not report any genomic/biological features of that particular species, so your phrasing is entirely correct.

Thank you for your comments. We have added this citation in the section of Introduction. The corresponding sentence was corrected as: "Thus far, whole genome sequence analysis has been reported for only one notothenioid species, the Antarctic rockcod Notothenia coriiceps (Shin et al., 2014). A major histocompatibility complex gene loci from Chaenocephalus aceratus was also reported (Malmstrøm M, et al (2016)."

Line 107-8: As you no doubt are aware of, size do not necessary have any bearing on buoyancy, only average density. It is not apparent to me that smaller size would mean easier to achieve neutral buoyancy.

Throughout the manuscript, we agree that neutral buoyancy is related to the average density of fish, not smaller size. Enhanced lipid storage and promotion of chondrogenesis while inhibiting osteogenesis in bone development play important roles for the D. mawsoni to achieve the neutral buoyancy.

We guess the misunderstanding of the reviewer might have resulted from our description on the evolution of smaller ZPC5 molecules in D. mawsoni, which is related to the enhanced capability of intracellular freezing-resistance in D. mawsoni, NOT related to neutral buoyancy, and nothing to do with body size of the fish.

Results:
Line 138: Why was two different genome assemblers used? Also, in the header for Table S2b it is stated that E. maclovinus was assembled with both SOAPdenovo and Platanus.

As we stated in answering reviewer #1's question, E. maclovinus DNA extracted from similarly prepared agarose plugs exhibited lower molecular weight than D. mawsoni for unknown reasons, which resulted in

lower quality of the E. maclovinus assemblies. To increase the E. maclovinus contig length and decrease algorithm bias when a single assembler was used, we parallelly built the contigs by two assemblers SOAPdenovo and Platanus. The generated contigs were merged prior to the scaffold building.

Line 140 and other places across the manuscript: "Kb", that is, kilo base pairs, should be abbreviated "kb(p)". See: https://en.wikipedia.org/wiki/Metric_prefix

Thank you for your comment. We check the abbreviation of "Kb" and "Kbp" in several journals. "Kb", as the abbreviation of kilo base pairs, is used in most of the journals. But for the abbreviation of less than 1000 base pairs, "bp" is used. So this manuscript betters to follow the universal usage, as "Kb".

Line 164: The number of common genes is a bit strange. The vast majority of genes should be common between these species. I think you have written this wrong. In the referred figure, S3, it is specified that the number 8,825 is the amount of common gene clusters, and not just genes. One cluster might contain multiple genes.

Thanks. That should be 8,825 gene clusters, not genes. We have corrected it in the revision.

Lines 182-192: You stated earlier "842 Mb for D. mawsoni and 727 Mb for E. maclovinus". You could say that quite a bit of that difference in genome size could be due to differences in repeat content, and not just percentage. 161.8 Mbp TEs in D. mawsoni and 74.6 Mbp in E. maclovinus, with a difference of 86.2 Mbp. It is not apparent that the percentages differences in repeat content actually translates to those large differences in repeats, because these repeat annotations can be quite different (many repeats are not annotated properly in different genomes).

Thank you for your suggestion. Annotation of the TEs in Antarctic toothfish, Patagonia robalo and Antarctic bullhead notothen (added in the revision) are conducted with the same pipelines and criteria. We compared TE contents (%) among the three genomes. Accumulation of TEs are observed in both Antarctic toothfish and bullhead notothen genomes, which may partially contributed the enlargement of genome size in the Antarctic notothenioids. We agree that repeat in genomes may not correctly annotated. We also correct two numbers, the TEs contents of D. mawsoni (21.38%) and E. maclovinus(10.02%), in this section, where the errors occurred in the previous version due to a mistake when citing from the results from the Additional file 1: Table S9.

Line 613: It is InterProScan, and not InterproScan.
Thanks. We have corrected it in the revision.

Close
