# Supplementary material for: The genomic basis for colonizing the freezing Southern Ocean revealed by Antarctic toothfish and Patagonian robalo genomes
Source: Gigascience. 2019 Jan 31;8(4):giz016. doi: 10.1093/gigascience/giz016 (PMC6457430; doi:10.1093/gigascience/giz016)
Supplement: Reviewer_2_Report_Original_Submission -- Baocheng Guo — 10/3/2018 Reviewed [file giz016_reviewer_2_report_original_submission.pdf]

Reviewer Report

Title: The genomic basis for colonizing the freezing Southern Ocean revealed by Antarctic toothfish and Patagonia robalo genomes

Version: Original Submission **Date:** 10/3/2018

Reviewer name: Baocheng Guo

Reviewer Comments to Author:

In this study, Chen and colleagues did genome sequencing of two notothenioids to understand genetic basis of Antarctic notothenioids adaptation to the Southern Ocean. I do think that the methodology of the study is sound and findings here are solid. The genomic resources are valuable for further studying genetic basis of the notothenioids adaptive radiation as the authors state. Before recommendation for its final publishing in GigaScience, I have the following concerns that authors might consider. 1) The authors might consider to incorporate the earlier sequenced notothenioid genome. I see that the authors have included the Antarctic bullhead notothen genome in their comparison analyses but do make fair comparison among the three notothenioid genomes. For example, the authors speculated that TE might contribute to genome size increasing in derived notothenioids by comparison between Antarctic toothfish and Patagonia robalo. What about TEs in the Antarctic bullhead notothen genome? What about LINEs in Antarctic bullhead notothen genome? A through comparison among the three notothenioid genomes might give us more information about the topic the authors are trying to beat. 2) As findings in their earlier works, the authors find that gene duplication plays an important role in adaptation to the freezing Southern Ocea in notothenioids. I am wondering how many gene families experienced duplication have been identified by both this study and their 2008 PNAS study. 3) The authors used an RNA-seq method for their study of transcriptomic adaptation to the freezing environment. However, I do not see any details how they collected the tissues, as we all know that such analysis is very sensitive to the sampling strategy.

Methods

Are the methods appropriate to the aims of the study, are they well described, and are necessary controls included? Choose an item.

Conclusions

Are the conclusions adequately supported by the data shown? Choose an item.

Reporting Standards

Does the manuscript adhere to the journal's guidelines on minimum standards of reporting? Choose an item.

Choose an item.

Statistics

Are you able to assess all statistics in the manuscript, including the appropriateness of statistical tests used? Choose an item.

Quality of Written English

Please indicate the quality of language in the manuscript: Choose an item.

Declaration of Competing Interests

Please complete a declaration of competing interests, considering the following questions:

- Have you in the past five years received reimbursements, fees, funding, or salary from an organisation that may in any way gain or lose financially from the publication of this manuscript, either now or in the future?
- Do you hold any stocks or shares in an organisation that may in any way gain or lose financially from the publication of this manuscript, either now or in the future?
- Do you hold or are you currently applying for any patents relating to the content of the manuscript?
- Have you received reimbursements, fees, funding, or salary from an organization that holds or has applied for patents relating to the content of the manuscript?
- Do you have any other financial competing interests?
- Do you have any non-financial competing interests in relation to this paper?

If you can answer no to all of the above, write 'I declare that I have no competing interests' below. If your reply is yes to any, please give details below.

I declare that I have no competing interests

I agree to the open peer review policy of the journal. I understand that my name will be included on my report to the authors and, if the manuscript is accepted for publication, my named report including any attachments I upload will be posted on the website along with the authors' responses. I agree for my report to be made available under an Open Access Creative Commons CC-BY license (http://creativecommons.org/licenses/by/4.0/). I understand that any comments which I do not wish to be included in my named report can be included as confidential comments to the editors, which will not be published.

I agree to the open peer review policy of the journal

To further support our reviewers, we have joined with Publons, where you can gain additional credit to further highlight your hard work (see: https://publons.com/journal/530/gigascience). On publication of this paper, your review will be automatically added to Publons, you can then choose whether or not to claim your Publons credit. I understand this statement.

Yes Choose an item.
