# Supplementary material for: The genomic basis for colonizing the freezing Southern Ocean revealed by Antarctic toothfish and Patagonian robalo genomes
Source: Gigascience. 2019 Jan 31;8(4):giz016. doi: 10.1093/gigascience/giz016 (PMC6457430; doi:10.1093/gigascience/giz016)
Supplement: Reviewer_3_Report_Original_Submission -- Ole K TÃ,rresen — 10/22/2018 Reviewed [file giz016_reviewer_3_report_original_submission.pdf]

Reviewer Report

Title: The genomic basis for colonizing the freezing Southern Ocean revealed by Antarctic toothfish and Patagonia robalo genomes

Version: Original Submission **Date:** 10/22/2018

Reviewer name: Ole K TÃ¸rresen

Reviewer Comments to Author:

Reviewer report.
Title: Genomic bases for colonizing the freezing Southern Ocean revealed by the genomes of Antarctic toothfish and Patagonia robalo
General comments
The authors have sequenced and assembled the genomes of two notothenioids, and have done extensive comparisons with regards to expansions of gene families and differential expression of genes. They show that several genes in the D. mawsoni has undergone positive selection, highlighting the evolution of the genes of that species.
Specific comments
Abstract: An extant species is not necessary a proxy for an extinct species.
Introduction:
Line 89-90: You specify "whole genome sequence analysis" as the criteria for mentioning the Antarctic rockcod as the only notothenioid reported so far, but MalmstrÃ¸m et al 2016 (https://www.nature.com/articles/ng.3645) did publish genomic sequences and the assembly of Chaenocephalus aceratus. However, they did not report any genomic/biological features of that particular species, so your phrasing is entirely correct.
Line 107-8: As you no doubt are aware of, size do not necessary have any bearing on buoyancy, only average density. It is not apparent to me that smaller size would mean easier to achieve neutral buoyancy.â€¨
â€¨
Results:
Line 138: Why was two different genome assemblers used? Also, in the header for Table S2b it is stated that E. maclovinus was assembled with both SOAPdenovo and Platanus.
Line 140 and other places across the manuscript: "Kb", that is, kilo base pairs, should be abbreviated "kb(p)". See: https://en.wikipedia.org/wiki/Metric_prefix
Line 164: The number of common genes is a bit strange. The vast majority of genes should be common between these species. I think you have written this wrong. In the referred figure, S3, it is specified that the number 8,825 is the amount of common gene clusters, and not just genes. One cluster might contain multiple genes.â€¨
Lines 182-192: You stated earlier "842 Mb for D. mawsoni and 727 Mb for E. maclovinus". You could say that quite a bit of that difference in genome size could be due to differences in repeat content, and not just percentage. 161.8 Mbp TEs in D. mawsoni and 74.6 Mbp in E. maclovinus, with a difference of 86.2

Mbp. It is not apparent that the percentages differences in repeat content actually translates to those large differences in repeats, because these repeat annotations can be quite different (many repeats are not annotated properly in different genomes).
Line 613: It is InterProScan, and not InterproScan.

Methods

Are the methods appropriate to the aims of the study, are they well described, and are necessary controls included? Choose an item.

Conclusions

Are the conclusions adequately supported by the data shown? Choose an item.

Reporting Standards

Does the manuscript adhere to the journal's guidelines on minimum standards of reporting? Choose an item.

Choose an item.

Statistics

Are you able to assess all statistics in the manuscript, including the appropriateness of statistical tests used? Choose an item.

Quality of Written English

Please indicate the quality of language in the manuscript: Choose an item.

Declaration of Competing Interests

Please complete a declaration of competing interests, considering the following questions:

- Have you in the past five years received reimbursements, fees, funding, or salary from an organisation that may in any way gain or lose financially from the publication of this manuscript, either now or in the future?
- Do you hold any stocks or shares in an organisation that may in any way gain or lose financially from the publication of this manuscript, either now or in the future?
- Do you hold or are you currently applying for any patents relating to the content of the manuscript?
- Have you received reimbursements, fees, funding, or salary from an organization that holds or has applied for patents relating to the content of the manuscript?
- Do you have any other financial competing interests?
- Do you have any non-financial competing interests in relation to this paper?

If you can answer no to all of the above, write 'I declare that I have no competing interests' below. If your reply is yes to any, please give details below.

I declare that I have no competing interests

I agree to the open peer review policy of the journal. I understand that my name will be included on my report to the authors and, if the manuscript is accepted for publication, my named report including any attachments I upload will be posted on the website along with the authors' responses. I agree for my report to be made available under an Open Access Creative Commons CC-BY license (http://creativecommons.org/licenses/by/4.0/). I understand that any comments which I do not wish to be included in my named report can be included as confidential comments to the editors, which will not be published.

I agree to the open peer review policy of the journal

To further support our reviewers, we have joined with Publons, where you can gain additional credit to further highlight your hard work (see: https://publons.com/journal/530/gigascience). On publication of this paper, your review will be automatically added to Publons, you can then choose whether or not to claim your Publons credit. I understand this statement.

Yes Choose an item.
